# Genome-wide transcriptome analysis reveals molecular pathways involved in leafy head formation of Chinese cabbage (*Brassica rapa*)

**DOI:** 10.1038/s41438-019-0212-9

**Published:** 2019-12-01

**Authors:** XiaoXue Sun, Ram Kumar Basnet, Zhichun Yan, Johan Bucher, Chengcheng Cai, Jianjun Zhao, Guusje Bonnema

**Affiliations:** 1 Key Laboratory of Vegetable Germplasm Innovation and Utilization of Hebei, Collaborative Innovation Center of Vegetable Industry in Hebei, Department of Horticulture, Hebei Agricultural University, Baoding, 071001 China; 2 Plant Breeding, Wageningen University and Research, Wageningen, 6708PB The Netherlands; 3 Quantitative genetics department, Rijk Zwaan Breeding B.V., Eerste Kruisweg 9, Fijnaart, 4793 RS The Netherlands

**Keywords:** Gene expression, Plant development

## Abstract

Chinese cabbage plants go through seedling and rosette stages before forming their leafy head. Chinese cabbage plants resemble pak-choi plants at their seedling stage, but in their rosette stage the leaves of Chinese cabbage differentiate, as they increase in size with shorter petioles. In order to understand the molecular pathways that play a role in leafy head formation, transcript abundance of young emerging leaves was profiled during development of two Chinese cabbage genotypes and a single pak-choi genotype. The two Chinese cabbages differed in many aspects, among others earliness, leaf size and shape, leaf numbers, and leafy head shape. Genome-wide transcriptome analysis clearly separated the seedling stages of all three genotypes together with the later stages from pak-choi, from the later developmental stages of both Chinese cabbages (rosette, folding, and heading). Weighted correlation network analysis and hierarchical clustering using Euclidean distances resulted in gene clusters with transcript abundance patterns distinguishing the two Chinese cabbages from pak-choi. Three clusters included genes with transcript abundance affected by both genotype and developmental stage, whereas two clusters showed only genotype effects. This included a genotype by developmental stage cluster highly enriched with the MapMan category photosynthesis, with high expression during rosette and folding in Chinese cabbages and low expression in the heading inner leaves that are not exposed to light. The other clusters contained many genes in the MapMan categories Cell, showing again differences between pak-choi and both Chinese cabbages. We discuss how this relates to the differences in leaf blade growth between Chinese cabbage and pak-choi, especially at the rosette stage. Overall, comparison of the transcriptome between leaves of two very different Chinese cabbages with pak-choi during plant development allowed the identification of specific gene categories associated with leafy head formation.

## Introduction

Chinese cabbage (CC; *Brassica rapa* ssp. *pekinensis*) is a widely cultivated and economically important vegetable in Asia, composed of a large number of tightly wrapped heading leaves (HLs) surrounding the shoot apexes^1^. The leafy head is the storage organ of CC and is formed after the rosette stage^2^. Head shapes of CCs vary depending on the cultivars. Developmental growth stages for CC have been described at the morphological level, including germination, seedling, rosette, folding, and heading stages^3^. At seedling stage, primary and juvenile leaves are round and have long petioles. At rosette stage, rosette leaves (RLs) become large and round with short petioles, which begin to fold upward. After the rosette stage, the incurving process of the leaves continues until HLs are arranged tightly around each other to form leafy heads as storage organs for nutrients. The HLs are wrinkled with an upward curvature with broad midveins. Both the size, shape, and degree of incurvature of HLs define the final head size and shape. The non-heading pak-choi (PC; *B. rapa* ssp. *Chinensis*) morphotype is closely related to heading CC as concluded from genetic diversity studies^4–6^. PC does not form leafy heads and the flat green leaves with fleshy petioles that grow as a rosette represent the consumed part of this leafy vegetable. In addition, the inner leaves remain green during PC development.

The morphology of leaves responds to cellular behaviors, such as cell size, cell shape, and the extent and orientation of cell division and expansion^7,8^. In addition, the size and shape of the leafy head correlates to that of the RLs^9,10^. However, the exact boundary between each developmental stage is not clear and the molecular mechanism during leafy head formation is still not well studied. In the study by Wang et al.^2^, a wide range of transcriptional events and interesting gene expression patterns were described analyzing RNA-sequencing data from RLs and folding leaves (FLs) of a typical heading CC (inbred line Fushanbaotou). Their results showed that stimuli such as carbohydrate levels, light, and hormones play important roles in leafy head development, and that the regulation of transcription factors, protein kinases, and calcium play important roles in this developmental process. In yet another study, a global analysis of microRNAs was performed in the rosette and heading stage of CC^11^. Besides identification of many conserved and novel miRNA’s, they also annotated the target genes of miRNA with differential expression patterns between rosette and heading stages, which included transcription factors, F-box proteins, protein kinases, auxin, and Ca signaling proteins, illustrating their roles in leafy head formation. One specific micro RNA, *microRNA156*, targets *BrSPL9–2*, which controls the heading time of CC^11^. In leafy heads of CC, *microRNA319* reduces the expression level of the *BrTCP4* gene controlling leaf cell proliferation and associates with a cylindrical head shape^9^.

The external leaves of CC leafy heads are green^12^. The inner head leaves are not exposed to the light and can be white, orange, or yellow^13^. These yellow inner leaves accumulate higher lutein and carotene content, but also other major metabolic processes take place, such as chlorophyll synthesis and degradation^2,12^. These processes may all be important for leafy head formation during the heading stage. By composite interval mapping analysis, three QTLs have been identified controlling the yellow color of the inner leaves in *B. rapa*^13^. In addition, other quantitative genetic studies identified QTLs for heading related traits, including the head top leaf overlap shape, head weight, head diameter, and head height, which also provides opportunities to genetically study the head formation in CC. Twenty-seven QTLs for gross weight, head weight, head length, head width, numbers of wrapper leaves, and head-forming leaves were detected in the Ge et al.^14^ study; 18 QTLs for 6 head traits were detected in the study of Yu et al.^15^; 4 QTLs for head leaf overlap shape, head height, head diameter, and head height-to-head diameter ratio were detected in the paper by Inoue et al.^16^.

In our study, we profiled transcript abundance in several developmental stages, including late seedling, rosette, folding, and heading stage. This was done for two different CC genotypes, an early and a late heading type with diverse head shapes and a non-heading PC. By comparing heading CC with non-heading PC, numerous genes that may specifically relate to leafy head formation with its inner yellow leaf development can be detected. Weighted correlation network analysis (WGCNA) resulted in identification of gene co-expression modules that differentiate between genotypes, between developmental stages, or between both. Our study provides new insights into understanding the genetic mechanisms of leafy head formation and the associating development of yellow leaves, highlighting the possibilities for studying CC leafy head formation at the cellular level.

## Results

### Comparison of morphology from heading CC and non-heading PC

In their vegetative growth phase, both CC and PC go through seedling and rosette stages. The RLs of PC have long petioles and the angle of the petioles also increase during growth till almost upright, depending on the genotype. The leaf blades however curl outwards, having an almost horizontal position. The RLs of CC become large and round with short petioles and upwards curving blades.

**Fig. 1:**
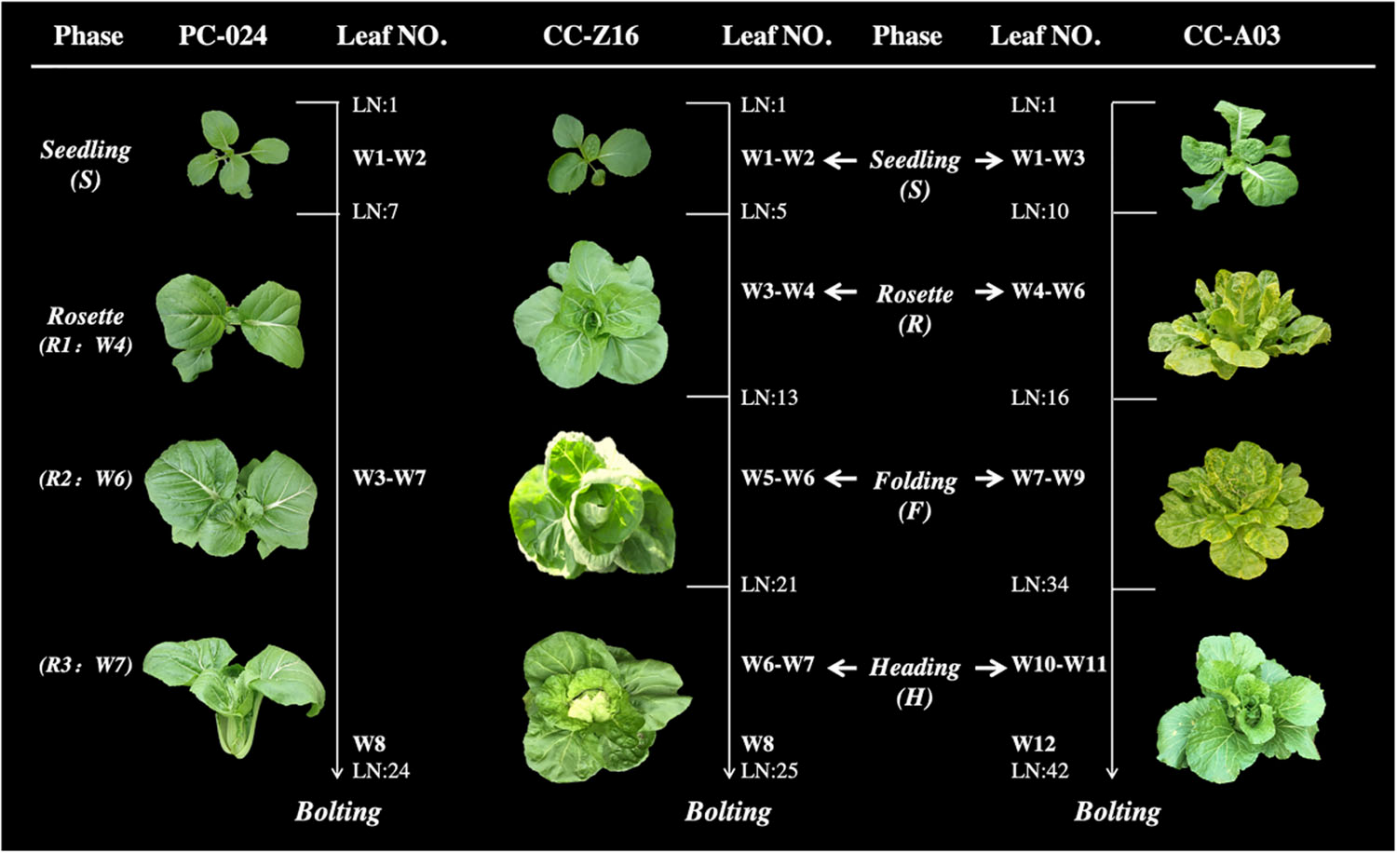
Morphological characterization of vegetative phases of the early heading Chinese cabbage CC-Z16 with round leafy head, the late heading CC-A03 with cylindrical head, and non-heading pak-choi PC-024. Seedling stage is indicated by “S,” rosette stage is indicated by “R,” folding stage is indicated by “F,” and heading stage is indicated by “H.” For PC-024, stages are indicated by “S,” “R1,” “R2,” and “R3.” In CC-A03, seedling stage is week 2, rosette stage is week 5, folding stage is week 8, and heading stage is week 11. In CC-Z16, seedling stage is week 2, rosette stage is week 4, folding stage is week 6, and heading stage is week 7. In PC-024, seedling stage is week 2, rosette stage R1 is week 4, R2 is week 6, and R3 is week 7.

In CC, this is followed by the folding stage after which the leafy head is formed (Fig. 1). In week 1 and week 2, plants were in the seedling stage (S), followed by the rosette stage (R), which is characterized by the onset of upward curving of leaves in CC-Z16 with younger emerging leaves having increased leaf angles. From week 5, the leaves of CC-Z16 become incurved with short petioles shaping a round head, which we refer to as folding stage. By contrast, CC-A03 develops slower, with a longer rosette stage, which is characterized by increasing leaf angles, around weeks 7 till 9 the folding stage, where leaves curve upwards forming a mold for the later head, and around week 10–11 the heading stage with further growth of the leafy head. At the folding stage (F), total leaf numbers of the late CC-A03 reaches 42, whereas CC-Z16 counts 25 leaves at this developmental stage, similar to the leaf number of the non-heading PC. At the heading stage (H), the two CCs have different head shapes: CC-A03 has a cylindrical head shape and CC-Z16 has a round head shape with overlapping leaves.

### Variation in transcript abundance during plant development

**Fig. 2:**
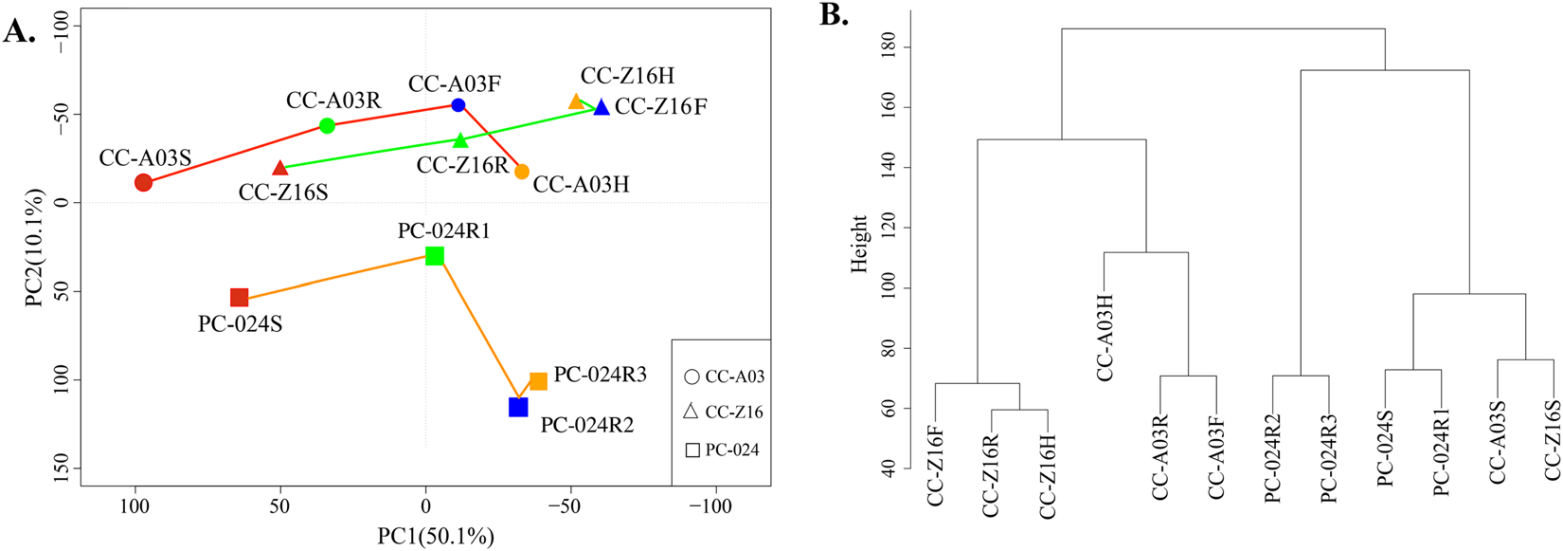
Sample analysis based on leaf transcript profiles during plant development. **a** Principal component analysis (PCA) of three genotypes (CC-A03, CC-Z16, and PC-024) based on transcript abundance in young leaves at four developmental stages: seedling (S), rosette (R), folding (F), and heading (H) stages. The red line represents heading Chinese cabbage CC-A03, the green line represents heading Chinese cabbage CC-Z16, and the orange line represents non-heading pak-choi PC-024. Sample labels were colored according to developmental stages: seedling stage (S)-red, rosette stage (R)-green, folding stage (F)-blue, and heading stage (H)-yellow. For PC-024, stages are (S)-red, rosette stage 1 to 3 (R1)-green, (R2)-blue, and (R3)-yellow. The samples were clustered based on the overall gene expression. **b** Cluster analysis by “ward” method for the three genotypes and four developmental stages (S, R/R1, F/R2, and H/R3). The average values of the two biological repeats were used.

Our aim was to study changes in transcript abundance in the four developmental stages, seedling, rosette, folding, and heading, which occur much later in CC-A03 then in the early CC-Z16. As PC-024 has no-heading stage and stays in the rosette stage after the seedling stage, but has similar developmental timing from seedling to bolting stage as CC-Z16, we isolated leaf tissue for the analysis at identical time points compared with CC-Z16. Biological repeats corresponded very well to each other as visualized by cluster analysis (Supplementary Fig. S1). Principal component analysis (PCA) of transcript abundance of the 61,654 probes on the used customer *B. rapa* array over the 3 genotypes and 4 developmental stages showed that plant developmental stages (S-seedling, R-rosette, F-folding, and H-heading stages) distribute along the first principal component (PC1) explaining 50.1% of the variation. Major changes in transcript abundance were observed between seedling (red symbol) to rosette (green symbol) and to folding stages (blue symbol) in all three genotypes. The folding (blue symbol) and heading stage (yellow symbol) for CC-Z16 and the rosette stages R2 and R3 for PC-024 form a tight group, whereas for CC-A03, the folding stage and heading stage were separated along the *x*-axis. The three genotypes (CC-Z16, CC-A03, and PC-024) separated in the second principal component (PC2), which explained 10.1% of the total variation. The two heading genotypes CC-Z16 and CC-A03 grouped together, separated from non-heading genotype PC-024 (Fig. 2a).

### Transcriptome analysis during CC and PC development

First, we looked at differences in transcript abundance between developmental stages of heading CC-Z16 and non-heading PC-024 sampled at the same time points (between seedling week 2 and rosette week 4, between rosette week 4 and folding week 6, and between folding week 6 and heading week 7). A threshold value of *p*-value ≤ 0.01 and an absolute value of log_2_Ratio ≥ 1.5 were chosen, which resulted in a selection of 4372 probes corresponding to 3727 genes with significant differences in transcript abundance. For these selected probes we plotted the numbers belonging to 34 MapMan functional categories for each comparison of developmental stages (Supplementary Fig. S2). Differentially expressed genes between heading CC-Z16 and non-heading PC-024 were highly represented by the following MapMan categories: development, transport, signaling, protein, RNA, stress, cell, hormone metabolism, cell wall, and photosynthesis. In addition, a large number of differentially expressed genes were not assigned to functional MapMan categories.

**Fig. 3:**
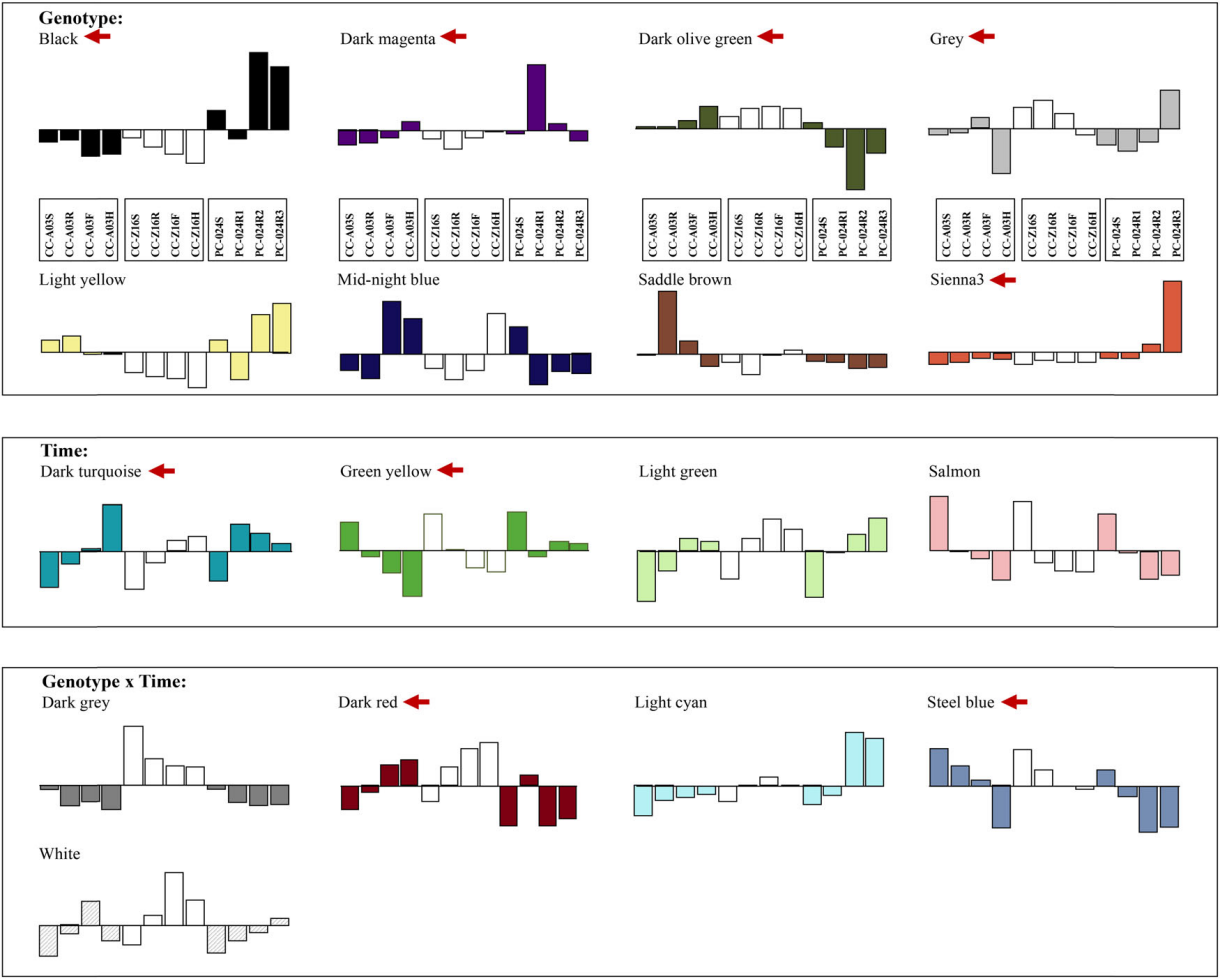
Barplots of the values of the 17 module eigengenes in CC-Z16, CC-A03, and PC-024. Left four bars represent CC-A03 seedling (week 2), rosette (week 5), folding (week 8), and heading (week 11) stages. Middle four bars represented CC-Z16 seedling (week 2), rosette (week 4), folding (week 7), and heading (week 8) stages. Right four bars represented PC-024 seedling (week 2) and rosette (R1: week 4, R2: week 5, and R3: week 7) stages. Selected modules with similar transcript abundance between CC-Z16 and CC-A03, but different from PC-024 that were selected for further analysis are marketed by red arrows.

Transcript abundance of these 4372 probes corresponding to 3727 genes was then analyzed by the WGCNA method using both the non-heading PC-024 and the two heading CC (CC-Z16 and CC-A03) transcriptome data. Seventeen clusters of highly correlated genes were defined by integration of the two biological repeats (Fig. 3), whereas cluster modules for two biological repeats analyzed separately are shown in Supplementary Fig. S3. Each module clusters genes with similar transcript abundance patterns either across genotypes (heading CC-Z16, CC-A03, and non-heading PC-024), across different developmental stages (hereafter referred to as phases), or across both genotype and phase. Analysis of variance (ANOVA) tests (*p*-value ≤ 0.05) showed that from all 17 modules, 8 (2337 probes) showed a genotype effect, 4 (736 probes) showed a phase effect, and 5 (1299 probes) showed both a genotype and phase effect (Supplementary Table S1). As our main interest was to identify genes that are involved in leafy head formation, based on the transcript abundance patterns for each module, nine modules (genotype effect: dark olive green, sienna, dark magenta, black, and gray; phase effect: dark turquoise and green yellow; genotype × phase effect: steel blue and dark red) with similar transcript abundance between CC-Z16 and CC-A03, but different from PC-024 were selected from 17 modules for further analysis.

### Selected modules with genotype, phase, or genotype-by-phase effect

The nine modules with contrasting patterns between the two CCs and PCs, consisting of two phase-affected modules (I and II), five genotype-affected modules (III, IV, V, VI, and VII), and two genotype-by-phase-affected modules (VIII, IX, and X) were analyzed by hierarchical clustering. Gene lists of nine modules are provided in Supplementary Table S2.

#### Phase-affected modules

**Fig. 4:**
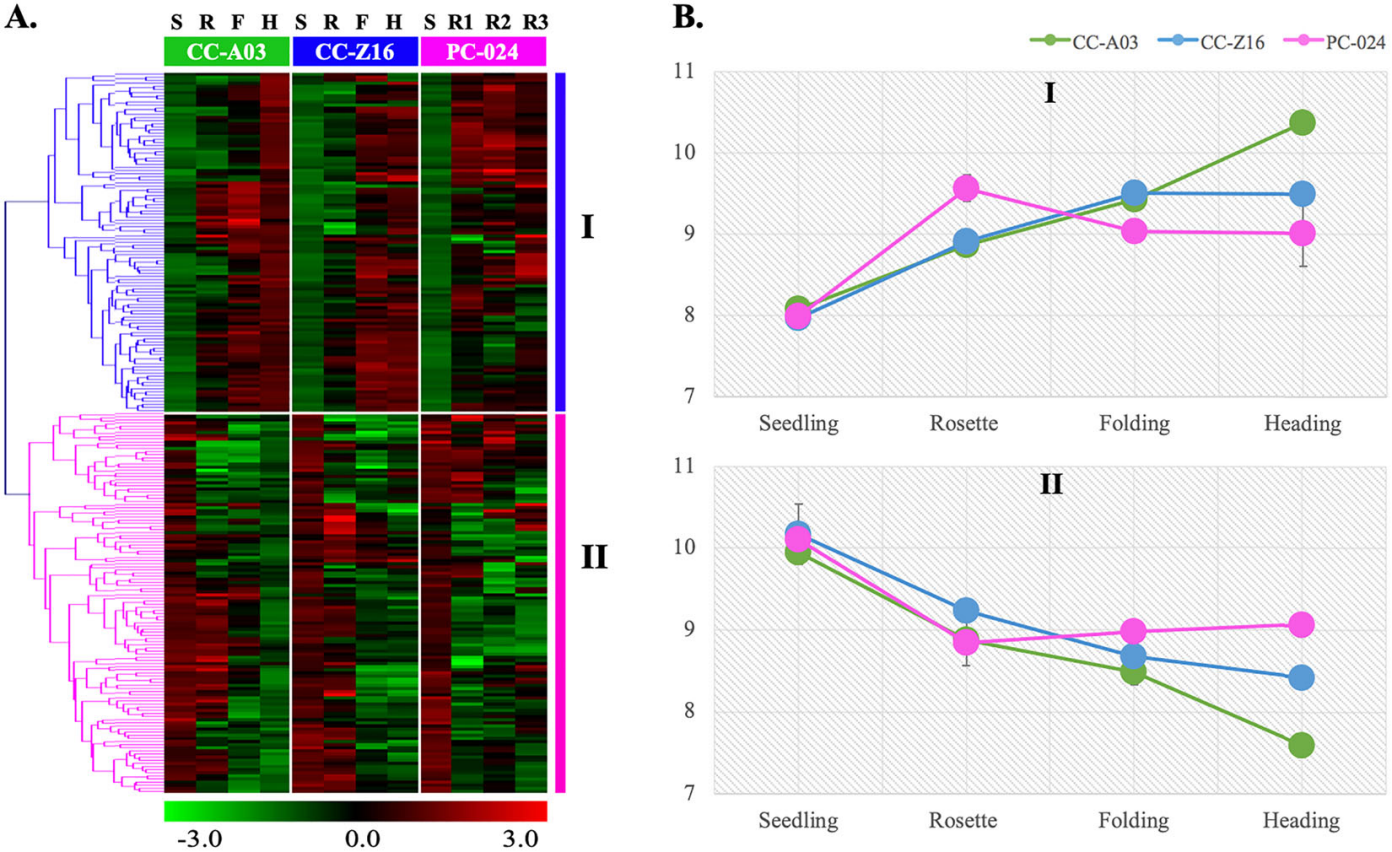
Transcript abundance clustering and profile of 231 genes across developmental stages. **a** Hierarchical cluster analysis using the probes from two WGCNA phase-affected modules (dark turquoise and green yellow) that differentiate the two Chinese cabbages (CC-Z16 and CCA-03) from PC-024. The heatmap shows transcript abundance across the developmental stages from seedling (S), rosette (R), folding (F), and heading (H) in CC-A03, CC-Z16, and similar time points for PC-024 as CC-Z16 (S, R1, R2, and R3). **b** Mean abundance of transcripts on three genotypes representing gene clusters I and II, respectively.

Hierarchical cluster analysis divided the 231 genes from the phase-affected modules into two clusters (Fig. 4a). Transcript abundance in these two clusters was significantly different between developmental stages in all three genotypes. Cluster I (109 genes) showed increased transcript abundance across the developmental stages, whereas cluster II (122 genes) showed decreased transcript abundance. The co-expression modules showed difference in patterns between heading CC and non-heading PC from rosette stage across plant development (Fig. 4b). In cluster I, transcript abundance of genes was increased from seedling to folding stage in both CC-A03 and CC-Z16, and increased further in CC-A03 till heading, but remained the same in CC-Z16. Genes in PC-024 had higher transcript abundance compared with CC at the rosette stage with an increased expression peak at week 5, and after a slight decrease expression was stable (week 6 and week 7).

Transcript abundance of genes from cluster II gradually decreased from seedling to heading stage in CC-A03 and CC-Z16. Genes had similar transcript abundance in PC compared with both CC genotypes from seedling to rosette stage, whereas transcript abundance remained constant in PC-024 after rosette stage.

#### Genotype-affected modules

**Fig. 5:**
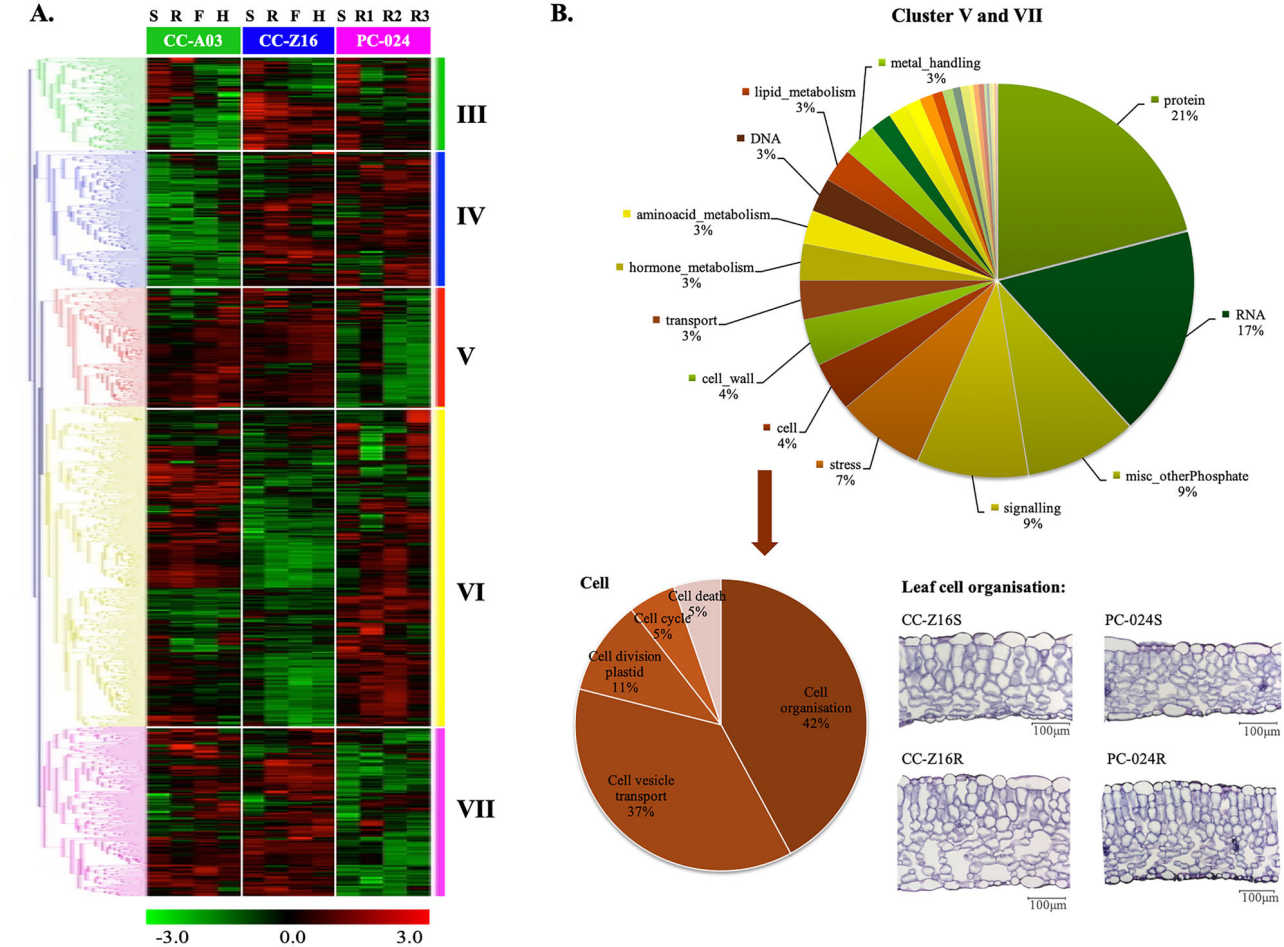
Hierarchical cluster analysis using the probes from five WGCNA genotype-affected modules that differentiate the two Chinese cabbages (CC-Z16 and CCA-03) from PC-024. **a** Probes from five genotype (CC-A03, CC-Z16, and PC-024)-affected modules. **b** Pie charts showing the percentage of genes that belong to MapMan functional categories in genotype effect clusters (V and VII) and genes belong to sub-categories in cell category. Transverse sections of the leaf of the heading Chinese cabbage (CC-Z16) and non-heading pak-choi (PC-024) at seedling (S) and rosette stage (R).

Hierarchical clustering divided the 1687 genes belonging to genotype-affected modules into five clusters (Fig. 5a). In gene clusters III (186 genes) and IV (277 genes), transcript abundance of CC-Z16 and PC-024 was more similar, but different from that of CC-A03. Whereas in gene cluster VI (644 genes) transcript abundance was more similar between CC-A03 and PC-024. The genes in clusters V (239 genes) and VII (341 genes) had similar transcript abundance patterns in the two CC genotypes compared with PC: high transcript abundance in both CC-Z16 and CC-A03 but low in PC-024. These genes may have roles in the leafy head formation typical for CCs.

Except not assigned probes, the numbers of probes belonging to MapMan functional categories are presented in Fig. 5b. We focussed on the main categories using a cutoff value of 3% of all genes in that cluster. The categories with percentages higher than 6% are indicated in the pie chart, whereas all categories are presented in Supplementary Table S3. The majority of the differentially expressed genes in the genotype effect clusters that differ between the two CCs and PCs (Cluster V and VII) were involved in protein (21%), RNA (17%), misc_other Phosphate (9%), various stimuli (9% signaling and 7% stress), cellular component (4% cell and 4% cell wall, total 8%), and some metabolism components of the hormone, lipid and amino acid metabolism categories.

As cell morphological traits (cell size, -shape, -arrangement and -number) of plants contribute largely to the final size and shape of the organs, we focused our analysis on the cell category. In the cell functional category from clusters V and VII, the majority of the differentially expressed genes were involved in cell organization (42%), cell vesicle transport (37%), and cell division (11%) sub-categories. As can be seen in both PCA analysis and the phase-affected modules (clusters I and II), most expression differences occur around the rosette stage. The cellular organization of leaves from PC and CC were compared at the seedling and rosette stage. At the seedling stage, leaves of PC and two CCs had a similar cellular organization; however, at the rosette stages cellular organization between PC and two CCs differed. Transverse sections illustrated that leaves of both morphotypes clearly displayed the typical dorsiventral structure, with palisade cells at the adaxial side and spongy cells at the abaxial side. The organization of palisade cells was more regular in PC-024 than in CC-Z16, especially at the rosette stage. Very remarkably, the intercellular spaces appeared larger in an upward curving CC-Z16 RLs, especially in the spongy parenchyma of the leaf abaxial side. Similar trends in cellular organization of leaves was found in another CC, with a cylindrical head shape, such as CC-A03 (Supplementary Fig. S4).

**Fig. 6:**
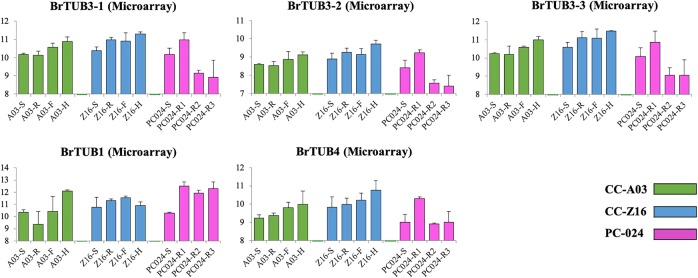
Transcript abundance of cell organization sub-category genes BrTUB3-1, BrTUB3-2, BrTUB3-3, BrTUB1, and BrTUB4 in CC-A03, CC-Z16, and PC-024 from microarray analysis. Green histogram represents CC-A03; blue histogram represents CC-Z16; pink histogram represents PC-024.

To further analyze the data, we identified genes with high degrees of connectedness within the clusters based on Pearson’s correlation coefficients. This resulted in a list ranking genes according to their numbers of connections, which in fact illustrates co-regulated networks. These top-ranking genes may function as key regulators of biological processes involved in CC or PC leaf development. For the clusters V and VII, which include genes that are higher expressed in the two CCs than in PC, the top 20 included 8 genes in “protein,” three in “cell,” and two in “RNA” categories (Pearson’s correlation coefficient > 0.9) (Supplementary Table S4). Cell organization sub-categories gene *BrTUB3–1* (*Bra018184*) was identified: transcript abundance was upregulated in the two CCs, but was downregulated in PC starting from the rosette stage. In addition, *BrTUB3-1*’s homologous gene *BrTUB3-2* (*Bra010114*) was also in the cell organization sub-category. *BrTUB3-2* had similar expression pattern as *BrTUB3-1*, but the transcript abundance was lower than that of *BrTUB3-1* (Fig. 6). The differential expression results were also confirmed by quantitative reverse transcription PCR (qRT-PCR) (Supplementary Fig. S5).

#### Genotype and phase-affected modules

**Fig. 7:**
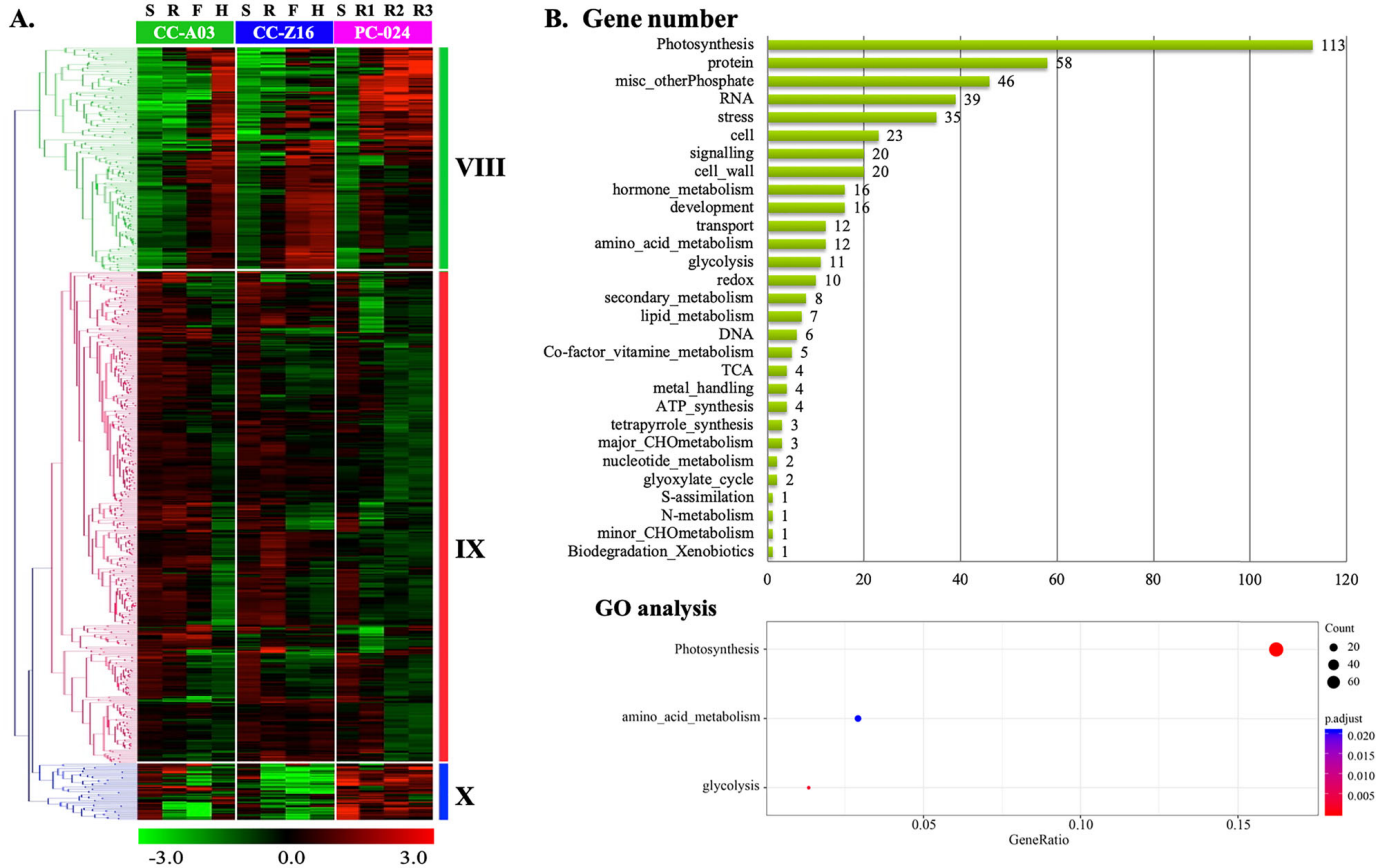
Hierarchical cluster analysis using the probes from two WGCNA genotype and phase-affected modules that differentiate the two Chinese cabbages (CC-Z16 and CC-A03) from PC-024. **a** The heatmap shows transcript abundance of genes that in WGCNA analysis showed genotype and developmental stage (phase)-affected modules. **b** Number of categorized annotated probes from clusters VIII, IX, and X, and GO enrichment analysis.

The 581 probes of the two modules displaying both genotype and phase effects were divided into three clusters (Fig. 7a). Transcript abundance of genes from cluster VIII (198 genes) gradually increased across developmental stages, with lower transcript abundance at the rosette stage in the two CC genotypes compared with PC. Genes in cluster IX (333 genes) showed a decrease of transcript abundance across developmental stages and a large number of genes showed higher transcript abundance in both CCs compared with PC at the rosette stage. Genes in cluster X (50 genes) had high transcript abundance levels in the seedling stage of all genotypes; however, at later stages the transcript abundance in PC remained high, whereas in both CCs the transcript abundance was low after the seedling stage. Cluster VIII, IX, and X were characterized by most pronounced similarity in transcript abundance in the two CCs, different from PC.

From the differently expressed genes in genotype × phase-affected clusters (VIII, IX, and X) the number of categorized annotated genes was depicted in Fig. 7b. The largest defined group of differentially regulated genes was photosynthesis category, with 113 genes (23% of 483), which contained 56 genes in light reaction (photosystem I and photosystem II), 26 genes in calvin cycle, and 22 genes in photorespiration. Besides the photosynthesis category, amino acid metabolism and glycolysis categories were identified by functional enrichment analysis.

Based on Pearson’s correlation coefficients analysis, the top-ranking 20 genes with high degrees of connectedness within the genotype × developmental stage-affected clusters IX and X included 11 “photosynthesis” genes and 6 not assigned genes (Supplementary Table S4). These top-ranking genes may function as key regulators of biological processes involved in CC or PC leaf development. Other functional categories included much less genes than the photosynthesis category: protein (12%), misc_otherPhosphate (9%), RNA (8%), stress (7%), and cell (5%). Interestingly, some of the genes in these categories can also be associated with photosynthesis-related processes such as chloroplast RNA binding in RNA category, germin-like proteins (GLP3 and GLP1) in stress category, isoprenoids carotenoids phytoene synthase (PSY), flavonoids dihydroflavonols (ATCHIL), and chloroplast lipoxygenase (LOX2) in hormone and secondary metabolism categories. Cluster VIII and IX are generated from probes in WGCNA genotype × phase-affected clusters with opposite expression patterns. Genes in cluster IX have lower expression at later stages, especially for PC samples. For cluster VIII, with both a genotype and a developmental stage effect, this top 20 also included five genes in the “protein” category, but very interestingly also six genes in the “cell” and “cell wall” classes, and also four in “glycolysis”. In cell category, several genes belong to the TUB family, *BrTUB1* (*Bra015815*), *BrTUB3-3* (*Bra019493*), and *BrTUB4* (*Bra033737*). *BrTUB3-3* and *BrTUB4* were upregulated in CC leaves, but were downregulated in PC leaves starting from rosette stage such as *BrTUB3-1* and *BrTUB3*. *BrTUB1* displayed higher transcript abundance at the rosette stage than at the seedling stage in PC, but not in CC (Fig. 6). The differential expression results were also confirmed by qRT-PCR (see Supplementary Fig. S5). In addition, the expression profiles of these genes were similar in an additional PC genotype and in a CC and a PC genotype that was grown in the open field in late summer in China (Supplementary Fig. S5).

## Discussion

In this study, we performed transcriptome analysis to get information about the processes and pathways that are important in leafy head formation of CCs. Leafy heads form through a series of complex developmental processes and are controlled by multiple internal and external signals. Others and we clearly showed that the leafy head trait is a quantitative trait, regulated by many QTLs with likely small effects^10,13–16^. In order to identify which developmental stages and pathways play major roles in governing leafy head formation in CC, we compared transcript abundance in two very different heading CCs and one non-heading PC during development.

### Comparing CC and PC

Although CC and PC are closely related, they have very different leaf shapes, surfaces, and sizes, and PCs do not form a leafy head^4,5,17^. We first compared CC-Z16 with PC-024. The commonality between the two is that they have similar growth period, from seedling (week 1) through rosette (week 4), to bolting (week 8) stage. In addition, the leaf surface of CC-Z16 is smooth, similar to PC, and not rugose, such as the leaves of most CCs. The differentially expressed genes selected in this group illustrate differences between heading and non-heading, as they likely exclude the effect of developmental age and leaf traits. The second comparison is between CC-Z16 and CC-A03. Although both are heading CCs, they differ in many aspects: their rosette and HLs have different shape and size. CC-Z16 leaves are smooth without an obvious mid-vein, whereas CC-A03 leaves have a rugose surface, as inter-vein cell expansion leads to an irregular surface, with a broad leaf mid-vein. Moreover, the rosette and heading stage in CC-A03 develop much later than in CC-Z16 with many more leaves and whereas head shape of CC-Z16 is round, head shape of CC-A03 is cylindrical. As we focus on genes with similar expression patterns between these two CCs, we expect that genes involved in leaf- and head size and shape are excluded from the analysis.

In the Wang et al.^2^ study, they compared the transcriptomes of RLs and FLs for the inbred CC line Fushanbaotou. They described the stages with leaf numbers: rosette stage with 8–10 expanded leaves and folding stage with 23–25 expanded leaves. We sampled leaves from CC-A03, which is a late heading CC-like Fushanbao, at comparable stages (rosette stage with 10–16 leaves, folding stage with16–34 leaves), but also looked at both earlier (seedling) and later (heading) stages. Based on our PCA analysis (Fig. 1), we conclude that Wang et al.^2^ only sampled a small part of the variation, compared with our study, as a large part of the variation occurs between seedling and rosette stages. As we selected genes that had different transcript abundance at more developmental stages in PC leaves on the one hand that differed from that of two CCs with similar transcript abundance on the other hand, this resulted in the identification of less differentially expressed genes compared with the Wang et al.^2^ study.

### Differentiation between CC and PC is initiated at the rosette stage

Leafy head size, shape, and timing vary according to the genetic makeup of the plant and are influenced by both internal (development, hormones, age) and external (environmental) factors. In PCA analysis, the first dimension separated the genotypes according to their developing stages; however, the second dimension (explaining 10% of the variation) separated the genotypes, the two CCs from PC. In the cluster analysis, the long rosette stage of PC (week 4 to week 7) formed a sub-cluster grouping together with seedling samples for all three genotypes in one cluster, which was clearly separated from the other cluster with rosette and heading stages of the two CCs, with sub-clusters dividing the two genotypes. These results indicate that the differences between CC and PC start from the rosette stage.

In addition, hierarchical clustering of genes revealed in WGCNA with significant genotype, developmental phase, or genotype by developmental phase effect assisted in the identification of clusters that clearly showed differences between the two CCs on the one hand and the single PC on the other hand. In the phase-affected clusters I and II, there were no obvious differences between genotypes and these most likely do not include genes that play specific roles in leafy head formation. However, clusters with clear genotype effects and especially those with genotype and phase effect are interesting and can reveal molecular pathways that are important in leafy head formation. Very interestingly, these clusters differed in the major MapMan categories to which their genes belonged.

### Functional categories of differentially expressed genes involved in leafy head formation

Interestingly, compared with earlier studies of the transcriptome of CCs^2^, we identified two additional categories: cell, cell wall functional categories (genotype affected clusters V/VII and genotype × phase-affected clusters XIII/X), and photosynthesis category in genotype × phase-affected cluster IX.

#### Photosynthesis category

Leaves, especially the RLs, serve as photosynthesizing organs and are thus important for nutrient absorption and plant growth^18^. Leafy heads increase very fast in volume and weight, by both forming many leaves from the SAM and by increase of the leaf size of the outer HLs. During this growth stage, the leafy head becomes very firm with tightly packed leaves. In this stage, the RLs are essential, as they need to generate the energy needed for the growing head, such as sugars and nitrogen for amino acids. RLs indeed increase in size and the senescence of these leaves is delayed^19^. It would be interesting to measure their photosynthesis efficiency during this growth stage. Compared with the RLs, the inner HLs of CC are storage organs that do not photosynthesize, as they are shielded from the light. These leaves have different characteristics in terms of nutritional value, taste, and color. In this study, we found significant differences in the expression of genes relating to photosynthesis in the genotype × phase-affected cluster IX. At the rosette stage, the photosynthesis genes have higher levels in the two CCs compared with PC and we hypothesize that this is to support the head formation by photosynthesis. In folding and especially heading stage, the inner leaves of CCs become covered by the outer HLs, become yellow, and transcript abundance of most photosynthesis genes decreased. The PC-024 green central leaves were exposed to the light during all developmental stages and indeed transcript levels did hardly change after the seedling stage. Interestingly, transcript abundance of photosynthesis genes was lower in PC leaves compared with CC leaves at the rosette and later rosette stages, likely because plants do not form a head that acts as a strong sink for nutrients. Recent papers showed that increased sink strength indeed can stimulate the rate of photosynthesis, which was studied in sweet potato, sugarcane, and legume species^20–22^. In addition, the photosynthesis efficiency is also affected by the light capture of leaves, which is influenced by the leaf-blade angle. It was shown in both rice and sorghum bicolor cultivars that their more erect leaves, with smaller leaf inclination angle, increased light capture, which resulted in higher grain yields^23,24^. This is consistent with the leaf phenotype in this study: the RLs of CC not only become large and round with short petioles, but also curve upwards, which likely affects photosynthetic efficiency and provides the sink strength for leafy head formation. In contrast, the PC leaf blades curve outwards, having an almost horizontal position. Further investigations of photosynthesis rates and genes are needed to fully comprehend the roles of the RLs in leafy head formation and may provide leads for breeders to improve head shape and size.

#### Cell category

Beside the differences in photosynthesis activity between the two CCs and PC, especially at the rosette stage, at this stage their leaf shapes are also different. CCs had large upward curving smooth or rugose RLs, whereas PC RLs are smooth and flat with narrow petioles that stand upward, while leaf blades are horizontal^25^. The leaf shape is defined by cell shape, size, and positioning^26^. We did observe the cell organization of both palisade and spongy parenchyma of RLs of CC-Z16 and PC-024, and an additional CC, CC-22, which similar to CC-A03 forms a cylindrical head, but the leaves are less rugose compared with CC-A03, making the comparison of leaf cellular composition feasible. This showed that CCs leaves tended to have both more and larger intercellular spaces especially in the spongy parenchyma, whereas PC-024 leaf cells were more densely packed. We hypothesize that looser organization of cells will facilitate leaf curving. Further quantification of cell numbers, size, and density across developmental stages and in several genotypes is needed to proof this hypothesis. On the molecular level, differentially expressed genes belonging to MapMan categories cell and cell wall were also represented in genotype-affected clusters V, VII, and genotype × phase-affected cluster VIII, which confirms that cell growth and division are important processes differentiating PC from CCs during their development. In the cell category, most genes belong to the cell organization sub-category and several tubulins such as *Bra018184* (*BrTUB3-1*), *Bra010144* (*BrTUB3-2*), *Bra019493* (*BrTUB3-3*), *Bra015815* (*BrTUB1*), and *Bra033737* (*BrTUB4*) were identified. In addition, *BrTUB3-1* and *BrTUB4* are also presented among the top 20 of genes with highest degrees of connectivity in clusters V, VII, and cluster VIII, respectively.

Assembly of α- and β- tubulins (TUA and TUB) regulates the form and orientation of microtubules, which are required for cell growth, cell replication, and cell division, and play an important role in the cell elongation process leading to normal plant morphology^27^. Some tubulin genes show different expression patterns during growth and development. In *Arabidopsis*, there are at least six α-, nine β-, and two γ-tubulin genes^28^. Different genes belonging to the *TUA* and *TUB* groups are highly similar to each other on the nucleotide level, but exhibit unique developmentally regulated patterns of expression in *Arabidopsis thaliana*^7,29^. For example, *AtTUB1* is expressed in the cortical cells of the roots^30^, *AtTUB9* is expressed in floral tissues^28^, and *AtTUB8* is expressed in the vegetative and reproductive organs’ vasculature^30^. In most studies, tubulin genes have non-overlapping expression patterns^28,31,32^. Interestingly, in this study we found that *BrTUB1*, *BrTUB3*, and *BrTUB4* all expressed in leaf tissue. In addition, *BrTUB1* and *BrTUB3* showed similar expression patterns. The tubulin gene families are being studied in considerable detail in *A. thaliana*^30^. In contrast, little is known about the tubulin gene families in Brassica. In this study, we defined a number of tubulin genes differentially expressed between CCs and PC, and consider that morphologcal differences between CCs and PC during development relate to leaf cell development/organization, thereby affecting the final leaf shape, size, and curvature behavior.

These results provide new insights in leafy head formation and also make evident that besides phenotyping the whole plant development, both morphological observation at cellular level and studies of photosynthetic processes are needed. The choice of two CCs with very different phenotypes narrowed down the selection of genes with differences in transcript abundance to better reveal the processes involved in leafy head development of CCs.

## Materials and methods

### Plant materials and growing conditions

In 2014, double haploid (DH) lines of heading CC (CC-A03, CC-Z16) and non-heading PC (PC-024, PC-184) were used in this study. The seeds of these four DH lines were germinated on seeding soil for one week and then transplanted into 17 cm pots. Pots were placed into three blocks in the greenhouse (Unifarm, Wageningen University & Research, 51^◦^59′11′′N latitude, 05^◦^39′52′′E longitude) and plants were grown under short day conditions from September.

In 2015, heading CC (Chiifu) and non-heading PC (PC-su) were sown in September in China, for gene transcript abundance study. Seeds were germinated in seedling soil for 1 week and then plants were transplanted to the field and grown under short day conditions at the Chinese Academy of Agricultural Sciences.

### Observations and sampling

For microarray study, three individual plants for each DH line (CC-A03, CC-Z16, and PC-024) per block were observed and numbers of leaves (leaf length > 1 cm) were counted once a week during plant growth in 2014. The developmental stage, such as rosette, folding, heading, and also bolting stages were evaluated each week. Young central leaves (around 2 cm long) surrounding the shoot apical meristem were collected at set intervals from three individual plants per block and were combined to one sample. Collection of the young emerging leaves in the center of the developing head at the heading stage of the CC lines (CC-A03 and CC-Z16) was destructive, as the outer head leaves needed to be removed in order to access the inner central leaves. Leaf samples were taken at comparable developmental stages for these three genotypes with different developmental timing. Leaf samples were harvested starting from week 2 after germination to week 12 for CC-A03 (week 2, week 5, week 8, and week 11) and week 2 to week 8 for CC-Z16 and PC-024 (week 2, week 4, week 6, and week 7). At each time point, two biological replicates (one per block) for each DH line were used for RNA isolation. In 2015, young central leaves (around 2 cm long) were collected from Chiifu and PC-su during plant development. Two biological repeats were used for RNA isolation and qRT-PCR.

### Expression analysis

The aim of this analysis was to gain insight into the transcriptome associated with CC leafy head formation by comparing heading CC with non-heading PC. Total RNA was isolated from the frozen leaf samples with “RNeasy Plant kit” (Qiagen) and treated with RNase-free DNase I (Invitrogen, Carisbad, CA, USA) to remove DNA contamination.

#### Microarray hybridization design

The microarray probes for the two-color Agilent microarray (Cy3-green/Cy5-red) platform were based on the predicted gene models of the reference *B. rapa* cv. Chiifu (a leafy vegetable inbred line) genome sequence^5^. This array assembly covers 61,654 probes and covers 40,879 (99.74%) *B. rapa* gene IDs (Bra ID) with 108 (0.26%) scaffold IDs. For detailed information, refer to Basnet et al.^33^. For microarray hybridization, we used developing leaves from three genotypes: two heading CC (CC-Z16 and CC-A03) and one non-heading PC (PC-024) at four phases (time points). The microarray design for this study is given in supplemental Table S5. Each slide contains eight arrays. Pairs of samples from two consecutive time points of the same genotype were hybridized, with two biological repeats, and eight arrays were used for one genotype.

#### Microarray data analysis

Microarray data were normalized within and between arrays using the limma package in R^34^. “Two Color Separate Channel” method was used to measure the gene transcript abundances. PCA was used to check the dominant modes of variation of all gene transcript abundance data. Then gene transcript abundance differences between three DH lines (genotype: CC-Z16, CC-A03, and PC-024) and between four developmental stages (phases) were determined with adjusted *p*-value ≤ 0.01 and fold change > 1.5 for further analysis.

WGCNA was used to find gene co-expression modules for genes with differential transcript abundance according to Pearson’s correlation^35^. Genes with similar co-expression patterns across genotypes, plant developmental stages, or their combinations were clustered into a module.

After defining gene modules, ANOVA tests were performed to determine the transcript abundance variation according to genotypes, developmental phase or both genotype and developmental phase at a 0.05 probability level. To cluster the probes at the level of transcript abundance, the hierarchical clustering using Euclidean distance was carried out separately for the probes from WGCNA genotype-affected modules, WGCNA phase-affected modules, or WGCNA genotype × phase-affected modules in the statistical software Multi-Experiment Viewer. The open source software MapMan was used for probes annotation in each category^36^.

For the co-expression network analysis, genes were selected after WGCNA and hierarchical cluster analysis. For each network analysis, gene pairs with at least Pearson’s correlation coefficient of 0.9 were considered. A parameter “degree of connection” of each node/gene was calculated in the statistical software R, indicating the number of connections (with Pearson’s correlation coefficient > 0.9) with other genes.

#### Quantitative real-time PCR

Transcript abundance of *B. rapa* genes (*BrTUB3-1*, *BrTUB3-2*, *BrTUB3-3*, *BrTUB1*, and *BrTUB4*), which were selected based on their expression patterns in the microarray study were determined by qRT-PCR. qRT-PCR reactions were performed with the Light Cycler-RNA amplification kit SYBR green I (Roche, Mannheim, Germany). Data were collected from two biological repeats. Actin was used as reference gene and primer sequences of candidate genes are listed in Supplementary Table S6.

## Supplementary information

Supplemental Figure S2

Supplementry Information
