# Supplementary material for: Genome-wide transcriptome analysis reveals molecular pathways involved in leafy head formation of Chinese cabbage (Brassica rapa)
Source: Hortic Res. 2019 Dec 1;6:130. doi: 10.1038/s41438-019-0212-9 (PMC6885048; doi:10.1038/s41438-019-0212-9)
Supplement: Supplementary file 2 — Supplementry Information [file 41438_2019_212_MOESM2_ESM.pdf]

Cluster	BraID	Bin	annotation	pathway
I	Bra002245	13	amino acid metabolism.synthesis.central amino acid metabolism.aspartate.aspartate aminotransferase	amino_acid_metabolism
I	Bra002245	13	amino acid metabolism.synthesis.central amino acid metabolism.aspartate.aspartate aminotransferase	amino_acid_metabolism
I	Bra025636	13	amino acid metabolism.synthesis.serine-glycine-cysteine group.cysteine	amino_acid_metabolism
I	Bra021412	25	C1-metabolism	C1-metabolism
I	Bra021412	25	C1-metabolism	C1-metabolism
I	Bra001062	25	C1-metabolism	C1-metabolism
I	Bra013952	31	cell.organisation	cell
I	Bra004786	31	cell.organisation	cell
I	Bra034402	31	cell.organisation	cell
I	Bra006686	31	cell.organisation	cell
I	Bra006686	31	cell.organisation	cell
I	Bra020276	31	cell.organisation	cell
I	Bra024346	31	cell.organisation	cell
I	Bra000540	10	cell wall.pectin*esterases.PME	cell_wall
I	Bra013164	10	cell wall.modification	cell_wall
I	Bra006980	33	development.unspecified	development
I	Bra001637	33	development.unspecified	development
I	Bra020519	33	development.unspecified	development
I	Bra037240	28	DNA.synthesis/chromatin structure.histone	DNA
I	Bra002942	5	fermentation.PDC	fermentation
I	Bra002942	5	fermentation.PDC	fermentation
I	Bra015693	5	fermentation.ADH	fermentation
I	Bra015693	5	fermentation.ADH	fermentation
I	Bra026452	4	glycolysis.unclear/dually targeted.phosphofructokinase (PFK)	glycolysis
I	Bra026452	4	glycolysis.unclear/dually targeted.phosphofructokinase (PFK)	glycolysis
I	Bra011387	4	glycolysis.unclear/dually targeted.phosphofructokinase (PFK)	glycolysis
I	Bra011387	4	glycolysis.unclear/dually targeted.phosphofructokinase (PFK)	glycolysis
I	Bra023756	17	hormone metabolism.ethylene.signal transduction	hormone_metabolism
I	Bra031090	17	hormone metabolism.ethylene.synthesis-degradation	hormone_metabolism
I	Bra027270	17	hormone metabolism.ethylene.signal transduction	hormone_metabolism
I	Bra002416	11	lipid metabolism.lipid degradation.beta-oxidation.acyl CoA reductase	lipid_metabolism
I	Bra002416	11	lipid metabolism.lipid degradation.beta-oxidation.acyl CoA reductase	lipid_metabolism
I	Bra031987	11	lipid metabolism.lipid degradation.lysophospholipases.phosphoinositide phospholipase C	lipid_metabolism
I	Bra022839	2	major CHO metabolism.degradation.sucrose.fructokinase	major_CHOmetabolism
I	Bra022839	2	major CHO metabolism.degradation.sucrose.fructokinase	major_CHOmetabolism
I	Bra028279	2	major CHO metabolism.degradation.sucrose.fructokinase	major_CHOmetabolism
I	Bra005720	15	metal handling.binding, chelation and storage	metal_handling
I	Bra000018	3	minor CHO metabolism.others	minor_CHOmetabolism
I	Bra014143	26	misc.cytochrome P450	misc_otherPhosphate
I	Bra012422	26	misc.glutathione S transferases	misc_otherPhosphate
I	Bra028838	26	misc.myrosinases-lectin-jacalin	misc_otherPhosphate
I	Bra013576	26	misc.peroxidases	misc_otherPhosphate
I	Bra024659	26	misc.glutathione S transferases	misc_otherPhosphate
I	Bra024659	26	misc.glutathione S transferases	misc_otherPhosphate
I	Bra013576	26	misc.peroxidases	misc_otherPhosphate
I	Bra026577	26	misc.plastocyanin-like	misc_otherPhosphate
I	Bra021291	26	misc.gluco-, galacto- and mannosidases	misc_otherPhosphate
I	Bra030386	26	misc.gluco-, galacto- and mannosidases	misc_otherPhosphate
I	Bra020696	26	misc.protease inhibitor/seed storage/lipid transfer protein (LTP) family protein	misc_otherPhosphate

I	Bra035997	26	misc.plastocyanin-like	misc_otherPhosphate
I	Bra014853	26	misc.protease inhibitor/seed storage/lipid transfer protein (LTP) family protein	misc_otherPhosphate
I	Bra014853	26	misc.protease inhibitor/seed storage/lipid transfer protein (LTP) family protein	misc_otherPhosphate
I	Bra018326	35	not assigned.unknown	not_assigned
I	Bra018326	35	not assigned.unknown	not_assigned
I	Bra000340	35	not assigned.unknown	not_assigned
I	Bra004803	35	not assigned.unknown	not_assigned
I	Bra022364	35	not assigned.unknown	not_assigned
I	Bra006805	35	not assigned.unknown	not_assigned
I	Bra031312	35	not assigned.unknown	not_assigned
I	Bra026796	35	not assigned.unknown	not_assigned
I	Bra008495	35	not assigned.unknown	not_assigned
I	Bra011484	35	not assigned.unknown	not_assigned
I	Bra020500	35	not assigned.unknown	not_assigned
I	Bra006627	35	not assigned.unknown	not_assigned
I	Bra007228	35	not assigned.unknown	not_assigned
I	Bra020576	35	not assigned.unknown	not_assigned
I	Bra037667	35	not assigned.unknown	not_assigned
I	Bra012602	1	PS.photorespiration.glycolate oxydase	Photosynthesis
I	Bra031585	29	protein.postranslational modification.kinase.receptor like cytoplasmatic kinase VII	protein
I	Bra031585	29	protein.postranslational modification.kinase.receptor like cytoplasmatic kinase VII	protein
I	Bra006663	29	protein.degradation.cysteine protease	protein
I	Bra009835	29	protein.degradation.ubiquitin.E3.SCF.FBOX	protein
I	Bra035183	29	protein.degradation.ubiquitin.E3.SCF.FBOX	protein
I	Bra015843	29	protein.degradation.ubiquitin.E2	protein
I	Bra031642	21	redox.dismutases and catalases	redox
I	Bra022667	27	RNA.regulation of transcription.General Transcription	RNA
I	Bra021433	27	RNA.regulation of transcription.AS2,Lateral Organ Boundaries Gene Family	RNA
I	Bra021433	27	RNA.regulation of transcription.AS2,Lateral Organ Boundaries Gene Family	RNA
I	Bra009915	27	RNA.regulation of transcription.unclassified	RNA
I	Bra009915	27	RNA.regulation of transcription.unclassified	RNA
I	Bra037041	27	RNA.regulation of transcription.AP2/EREBP, APETALA2/Ethylene-responsive element binding protein family	RNA
I	Bra037041	27	RNA.regulation of transcription.AP2/EREBP, APETALA2/Ethylene-responsive element binding protein family	RNA
I	Bra021200	27	RNA.regulation of transcription.AP2/EREBP, APETALA2/Ethylene-responsive element binding protein family	RNA
I	Bra021200	27	RNA.regulation of transcription.AP2/EREBP, APETALA2/Ethylene-responsive element binding protein family	RNA
I	Bra011082	27	RNA.regulation of transcription.Aux/IAA family	RNA
I	Bra026847	27	RNA.processing.ribonucleases	RNA
I	Bra014655	16	secondary metabolism.N misc.alkaloid-like	secondary_metabolism
I	Bra016865	30	signalling.light	signalling
I	Bra009235	30	signalling.receptor kinases.leucine rich repeat XI	signalling
I	Bra001460	30	signalling.receptor kinases.leucine rich repeat XI	signalling
I	Bra001460	30	signalling.receptor kinases.leucine rich repeat XI	signalling
I	Bra023876	30	signalling.phosphorelay	signalling
I	Bra023876	30	signalling.phosphorelay	signalling
I	Bra027169	30	signalling.phosphorelay	signalling
I	Bra014935	30	signalling.light	signalling
I	Bra029821	30	signalling.calcium	signalling
I	Bra009040	30	signalling.G-proteins	signalling
I	Bra003483	30	signalling.light	signalling

I	Bra031672	30	signalling.light	signalling
I	Bra012707	20	stress.abiotic.heat	stress
I	Bra012397	20	stress.abiotic.unspecified	stress
I	Bra012414	20	stress.abiotic.unspecified	stress
I	Bra012414	20	stress.abiotic.unspecified	stress
I	Bra032889	20	stress.abiotic.unspecified	stress
I	Bra000312	20	stress.biotic	stress
I	Bra021214	20	stress.abiotic.cold	stress
I	Bra016809	8	TCA / org. transformation.TCA.pyruvate DH.E2	TCA
I	Bra033181	34	transport.Major Intrinsic Proteins.TIP	transport
I	Bra003906	34	transport.sugars.sucrose	transport
II	Bra011405	13	amino acid metabolism.degradation.serine-glycine-cysteine group.glycine	amino_acid_metabolism
II	Bra011405	13	amino acid metabolism.degradation.serine-glycine-cysteine group.glycine	amino_acid_metabolism
II	Bra012218	10	cell wall.modification	cell_wall
II	Bra012218	10	cell wall.modification	cell_wall
II	Bra036610	10	cell wall.pectin*esterases.PME	cell_wall
II	Bra018372	18	Co-factor and vitamine metabolism.thiamine	Co-factor_vitamine_metabolism
II	Bra005687	18	Co-factor and vitamine metabolism	Co-factor_vitamine_metabolism
II	Bra002957	18	Co-factor and vitamine metabolism.thiamine	Co-factor_vitamine_metabolism
II	Bra016184	4	glycolysis.plastid branch.phosphoglucomutase (PGM)	glycolysis
II	Bra033396	17	hormone metabolism.ethylene.synthesis-degradation	hormone_metabolism
II	Bra011889	17	hormone metabolism.auxin.induced-regulated-responsive-activated	hormone_metabolism
II	Bra011891	17	hormone metabolism.auxin.induced-regulated-responsive-activated	hormone_metabolism
II	Bra024530	17	hormone metabolism.gibberelin.induced-regulated-responsive-activated	hormone_metabolism
II	Bra031749	2	major CHO metabolism.degradation.starch.glucan water dikinase	major_CHOmetabolism
II	Bra031749	2	major CHO metabolism.degradation.starch.glucan water dikinase	major_CHOmetabolism
II	Bra018423	2	major CHO metabolism.degradation.starch.glucan water dikinase	major_CHOmetabolism
II	Bra004590	2	major CHO metabolism.degradation.starch.D enzyme	major_CHOmetabolism
II	Bra000777	26	misc.protease inhibitor/seed storage/lipid transfer protein (LTP) family protein	misc_otherPhosphate
II	Bra000777	26	misc.protease inhibitor/seed storage/lipid transfer protein (LTP) family protein	misc_otherPhosphate
II	Bra000778	26	misc.protease inhibitor/seed storage/lipid transfer protein (LTP) family protein	misc_otherPhosphate
II	Bra000778	26	misc.protease inhibitor/seed storage/lipid transfer protein (LTP) family protein	misc_otherPhosphate
II	Bra034519	26	misc.protease inhibitor/seed storage/lipid transfer protein (LTP) family protein	misc_otherPhosphate
II	Bra030156	26	misc.GDSL-motif lipase	misc_otherPhosphate
II	Bra017304	26	misc.oxidases - copper, flavone etc.	misc_otherPhosphate
II	Bra037336	26	misc.cytochrome P450	misc_otherPhosphate
II	Bra037336	26	misc.cytochrome P450	misc_otherPhosphate
II	Bra000768	26	misc.cytochrome P450	misc_otherPhosphate
II	Bra032915	26	misc.GDSL-motif lipase	misc_otherPhosphate
II	Bra011060	26	misc.GDSL-motif lipase	misc_otherPhosphate
II	Bra001564	26	misc.cytochrome P450	misc_otherPhosphate
II	Bra018544	26	misc.protease inhibitor/seed storage/lipid transfer protein (LTP) family protein	misc_otherPhosphate
II	Bra018544	26	misc.protease inhibitor/seed storage/lipid transfer protein (LTP) family protein	misc_otherPhosphate
II	Bra040762	26	misc.cytochrome P450	misc_otherPhosphate
II	Bra009483	12	N-metabolism.ammonia metabolism.glutamate synthase	N-metabolism
II	Bra009302	35	not assigned.unknown	not_assigned
II	Bra002680	35	not assigned.unknown	not_assigned
II	Bra009881	35	not assigned.unknown	not_assigned
II	Bra017920	35	not assigned.unknown	not_assigned
II	Bra039995	35	not assigned.unknown	not_assigned
II	Bra010015	35	not assigned.unknown	not_assigned
II	Bra006086	35	not assigned.unknown	not_assigned

II	Bra036492	35	not assigned.unknown	not_assigned
II	Bra036492	35	not assigned.unknown	not_assigned
II	Bra009609	35	not assigned.unknown	not_assigned
II	Bra037942	35	not assigned.unknown	not_assigned
II	Bra006852	35	not assigned.unknown	not_assigned
II	Bra018169	35	not assigned.unknown	not_assigned
II	Bra018169	35	not assigned.unknown	not_assigned
II	Bra019494	35	not assigned.unknown	not_assigned
II	Bra028483	35	not assigned.unknown	not_assigned
II	Bra018005	35	not assigned.unknown	not_assigned
II	Bra031336	35	not assigned.unknown	not_assigned
II	Bra037462	35	not assigned.unknown	not_assigned
II	Bra038730	35	not assigned.unknown	not_assigned
II	Bra012627	35	not assigned.unknown	not_assigned
II	Bra025921	35	not assigned.unknown	not_assigned
II	Bra011918	35	not assigned.unknown	not_assigned
II	Bra024753	35	not assigned.unknown	not_assigned
II	Bra027784	35	not assigned.unknown	not_assigned
II	Bra001684	23	nucleotide metabolism.degradation.pyrimidine.dihydrouracil dehydrogenase	nucleotide_metabolism
II	Bra013183	1	PS.lightreaction.photosystem II.LHC-II	Photosynthesis
II	Bra000708	1	PS.lightreaction.photosystem II.LHC-II	Photosynthesis
II	Bra000708	1	PS.lightreaction.photosystem II.LHC-II	Photosynthesis
II	Bra016481	1	PS.lightreaction.other electron carrier (ox/red).ferredoxin reductase	Photosynthesis
II	Bra012203	1	PS.lightreaction.other electron carrier (ox/red).ferredoxin reductase	Photosynthesis
II	Bra003499	1	PS.calvin cycle	Photosynthesis
II	Bra003499	1	PS.calvin cycle	Photosynthesis
II	Bra028087	1	PS.calvin cycle.rubisco large subunit	Photosynthesis
II	Bra028087	1	PS.calvin cycle.rubisco large subunit	Photosynthesis
II	Bra016481	1	PS.lightreaction.other electron carrier (ox/red).ferredoxin reductase	Photosynthesis
II	Bra024401	29	protein.degradation	protein
II	Bra010379	29	protein.degradation.ubiquitin.E3.RING	protein
II	Bra034383	29	protein.folding	protein
II	Bra019474	29	protein.postranslational modification	protein
II	Bra019427	29	protein.postranslational modification	protein
II	Bra005637	29	protein.degradation	protein
II	Bra003055	29	protein.postranslational modification	protein
II	Bra001867	29	protein.postranslational modification	protein
II	Bra008811	29	protein.synthesis.elongation	protein
II	Bra010384	29	protein.degradation.cysteine protease	protein
II	Bra036642	21	redox.dismutases and catalases	redox
II	Bra013863	21	redox.dismutases and catalases	redox
II	Bra026398	21	redox.ascorbate and glutathione.ascorbate.GDP-L-galactose-hexose-1-phosphate guanyltransferase	redox
II	Bra034674	21	redox.dismutases and catalases	redox
II	Bra004503	27	RNA.regulation of transcription.MYB-related transcription factor family	RNA
II	Bra016156	27	RNA.regulation of transcription.bHLH,Basic Helix-Loop-Helix family	RNA
II	Bra033726	27	RNA.regulation of transcription.G2-like transcription factor family, GARP	RNA
II	Bra029311	27	RNA.regulation of transcription.MYB domain transcription factor family	RNA
II	Bra018141	27	RNA.regulation of transcription.C2C2(Zn) DOF zinc finger family	RNA
II	Bra018141	27	RNA.regulation of transcription.C2C2(Zn) DOF zinc finger family	RNA
II	Bra024750	27	RNA.regulation of transcription.C2C2(Zn) CO-like, Constans-like zinc finger family	RNA
II	Bra022516	27	RNA.regulation of transcription.unclassified	RNA

II	Bra031129	27	RNA.regulation of transcription.G2-like transcription factor family, GARP	RNA
II	Bra031129	27	RNA.regulation of transcription.G2-like transcription factor family, GARP	RNA
II	Bra004035	27	RNA.regulation of transcription.C2C2(Zn) CO-like, Constans-like zinc finger family	RNA
II	Bra004035	27	RNA.regulation of transcription.C2C2(Zn) CO-like, Constans-like zinc finger family	RNA
II	Bra015787	27	RNA.regulation of transcription.Nucleosome/chromatin assembly factor group	RNA
II	Bra033925	27	RNA.regulation of transcription.C2C2(Zn) CO-like, Constans-like zinc finger family	RNA
II	Bra012475	27	RNA.regulation of transcription.C2C2(Zn) CO-like, Constans-like zinc finger family	RNA
II	Bra018565	27	RNA.regulation of transcription.unclassified	RNA
II	Bra027196	27	RNA.regulation of transcription.Orphan family	RNA
II	Bra036865	27	RNA.processing.splicing	RNA
II	Bra004109	16	secondary metabolism.phenylpropanoids.lignin biosynthesis.4CL	secondary_metabolism
II	Bra025164	16	secondary metabolism.isoprenoids.terpenoids	secondary_metabolism
II	Bra036883	30	signalling.light	signalling
II	Bra026425	30	signalling.receptor kinases.leucine rich repeat XI	signalling
II	Bra016372	30	signalling.G-proteins	signalling
II	Bra021912	30	signalling.light	signalling
II	Bra012942	30	signalling.receptor kinases.leucine rich repeat III	signalling
II	Bra016839	30	signalling.receptor kinases.DUF 26	signalling
II	Bra020865	20	stress.abiotic.heat	stress
II	Bra013566	20	stress.abiotic.heat	stress
II	Bra016047	20	stress.abiotic.unspecified	stress
II	Bra016047	20	stress.abiotic.unspecified	stress
II	Bra018700	8	TCA / org. transformation.TCA.pyruvate DH.E3	TCA
II	Bra038646	19	tetrapyrrole synthesis.GSA	tetrapyrrole_synthesis
II	Bra024934	34	transport.unspecified cations	transport
II	Bra025740	34	transport.sugars	transport
II	Bra024802	34	transport.sugars.sucrose	transport
II	Bra039469	34	transport.unspecified cations	transport
II	Bra039469	34	transport.unspecified cations	transport
II	Bra009203	34	transport.ABC transporters and multidrug resistance systems	transport
Cluster	BraID	Bin	annotation	pathway
III	Bra015504	3	minor CHO metabolism.callose	minor_CHOmetabolism
III	Bra000294	6	gluconeogenese/ glyoxylate cycle.isocitrate lyase	glyoxylate_cycle
III	Bra005687	18	Co-factor and vitamine metabolism	Co-factor_vitamine_metabolism
III	Bra000603	18	Co-factor and vitamine metabolism	Co-factor_vitamine_metabolism
III	Bra007748	19	tetrapyrrole synthesis.ferrochelatase	tetrapyrrole_synthesis
III	Bra000234	21	redox.thioredoxin	redox
III	Bra006467	21	redox.glutaredoxins	redox
III	Bra006467	21	redox.glutaredoxins	redox
III	Bra023688	21	redox.glutaredoxins	redox
III	Bra023688	21	redox.glutaredoxins	redox
III	Bra016459	21	redox.dismutases and catalases	redox
III	Bra004810	25	C1-metabolism	C1-metabolism
III	Bra035818	26	misc.cytochrome P450	misc_otherPhosphate
III	Bra001564	26	misc.cytochrome P450	misc_otherPhosphate
III	Bra002270	26	misc.peroxidases	misc_otherPhosphate
III	Bra005002	26	misc.acid and other phosphatases	misc_otherPhosphate
III	Bra018283	26	misc.plastocyanin-like	misc_otherPhosphate
III	Bra002442	26	misc.UDP glucosyl and glucoronyl transferases	misc_otherPhosphate
III	Bra004088	26	misc.UDP glucosyl and glucoronyl transferases	misc_otherPhosphate
III	Bra005227	26	misc.UDP glucosyl and glucoronyl transferases	misc_otherPhosphate

III	Bra014833	26	misc.protease inhibitor/seed storage/lipid transfer protein (LTP) family protein	misc_otherPhosphate
III	Bra029584	26	misc.gluco-, galacto- and mannosidases	misc_otherPhosphate
III	Bra012981	26	misc.beta 1,3 glucan hydrolases	misc_otherPhosphate
III	Bra012981	26	misc.beta 1,3 glucan hydrolases	misc_otherPhosphate
III	Bra003707	26	misc.O-methyl transferases	misc_otherPhosphate
III	Bra003707	26	misc.O-methyl transferases	misc_otherPhosphate
III	Bra026988	26	misc.oxidases - copper, flavone etc.	misc_otherPhosphate
III	Bra018405	26	misc.nitrilases, *nitrile lyases, berberine bridge enzymes, reticuline oxidases, troponine reductases	misc_otherPhosphate
III	Bra031612	28	DNA.synthesis/chromatin structure	DNA
III	Bra007297	28	DNA.synthesis/chromatin structure	DNA
III	Bra016776	28	DNA.repair	DNA
III	Bra035000	29	protein.postranslational modification	protein
III	Bra006263	29	protein.postranslational modification	protein
III	Bra032950	29	protein.postranslational modification	protein
III	Bra027982	29	protein.degradation	protein
III	Bra011985	29	protein.folding	protein
III	Bra011985	29	protein.folding	protein
III	Bra029516	29	protein.assembly and cofactor ligation	protein
III	Bra016926	30	signalling.calcium	signalling
III	Bra016926	30	signalling.calcium	signalling
III	Bra036797	30	signalling.phosphinositides	signalling
III	Bra034781	30	signalling.G-proteins	signalling
III	Bra009969	31	cell.organisation	cell
III	Bra013952	31	cell.organisation	cell
III	Bra036194	31	cell.organisation	cell
III	Bra013416	31	cell.organisation	cell
III	Bra008470	31	cell.organisation	cell
III	Bra010409	33	development.storage proteins	development
III	Bra039956	33	development.late embryogenesis abundant	development
III	Bra028058	33	development.unspecified	development
III	Bra008693	33	development.unspecified	development
III	Bra040486	33	development.unspecified	development
III	Bra036522	33	development.unspecified	development
III	Bra011565	33	development.unspecified	development
III	Bra013162	33	development.unspecified	development
III	Bra004412	33	development.unspecified	development
III	Bra009789	33	development.unspecified	development
III	Bra021584	33	development.unspecified	development
III	Bra004412	33	development.unspecified	development
III	Bra002733	34	transport.p- and v-ATPases	transport
III	Bra029519	34	transport.potassium	transport
III	Bra003208	34	transport.ABC transporters and multidrug resistance systems	transport
III	Bra002664	34	transport.ABC transporters and multidrug resistance systems	transport
III	Bra031898	34	transport.ABC transporters and multidrug resistance systems	transport
III	Bra009636	34	transport.amino acids	transport
III	Bra019688	34	transport.phosphate	transport
III	Bra019690	34	transport.phosphate	transport
III	Bra002068	34	transport.misc	transport
III	Bra018368	35	not assigned.unknown	not_assigned
III	Bra020332	35	not assigned.unknown	not_assigned
III	Bra005994	35	not assigned.unknown	not_assigned

III	Bra028171	35	not assigned.unknown	not_assigned
III	Bra016484	35	not assigned.unknown	not_assigned
III	Bra037850	35	not assigned.unknown	not_assigned
III	Bra038884	35	not assigned.unknown	not_assigned
III	Bra020118	35	not assigned.unknown	not_assigned
III	Bra024218	35	not assigned.unknown	not_assigned
III	Bra033150	35	not assigned.unknown	not_assigned
III	Bra004750	35	not assigned.unknown	not_assigned
III	Bra004750	35	not assigned.unknown	not_assigned
III	Bra025412	35	not assigned.unknown	not_assigned
III	Bra006986	35	not assigned.unknown	not_assigned
III	Bra019739	35	not assigned.unknown	not_assigned
III	Bra007246	35	not assigned.unknown	not_assigned
III	Bra038118	35	not assigned.unknown	not_assigned
III	Bra004865	35	not assigned.unknown	not_assigned
III	Bra035253	35	not assigned.unknown	not_assigned
III	Bra024855	35	not assigned.unknown	not_assigned
III	Bra024302	35	not assigned.unknown	not_assigned
III	Bra033973	35	not assigned.unknown	not_assigned
III	Bra010230	35	not assigned.unknown	not_assigned
III	Bra006928	35	not assigned.unknown	not_assigned
III	Bra035582	35	not assigned.unknown	not_assigned
III	Bra011361	35	not assigned.unknown	not_assigned
III	Bra006367	35	not assigned.unknown	not_assigned
III	Bra002782	35	not assigned.unknown	not_assigned
III	Bra006737	35	not assigned.unknown	not_assigned
III	Bra018696	35	not assigned.unknown	not_assigned
III	Bra041115	35	not assigned.unknown	not_assigned
III	Bra026738	35	not assigned.unknown	not_assigned
III	Bra035837	35	not assigned.unknown	not_assigned
III	Bra035837	35	not assigned.unknown	not_assigned
III	Bra014790	35	not assigned.unknown	not_assigned
III	Bra031439	35	not assigned.unknown	not_assigned
III	Bra021057	35	not assigned.unknown	not_assigned
III	Bra036634	35	not assigned.unknown	not_assigned
III	Bra022719	35	not assigned.unknown	not_assigned
III	Bra041142	35	not assigned.unknown	not_assigned
III	Bra039512	10	cell wall.precursor synthesis.MUR4	cell_wall
III	Bra003934	11	lipid metabolism.FA synthesis and FA elongation.beta ketoacyl CoA synthase	lipid_metabolism
III	Bra026522	11	lipid metabolism."exotics" (steroids, squalene etc).sphingolipids	lipid_metabolism
III	Bra015656	12	N-metabolism.nitrate metabolism.NR	N-metabolism
III	Bra016810	13	amino acid metabolism.degradation.serine-glycine-cysteine group.glycine	amino_acid_metabolism
III	Bra015739	16	secondary metabolism.isoprenoids.mevalonate pathway.HMG-CoA reductase	secondary_metabolism
III	Bra039167	17	hormone metabolism.auxin.signal transduction	hormone_metabolism
III	Bra015447	17	hormone metabolism.salicylic acid.synthesis-degradation	hormone_metabolism
III	Bra016091	2	major CHO metabolism.degradation.sucrose.invertases.neutral	major_CHOmetabolism
III	Bra013872	20	stress.abiotic.heat	stress
III	Bra026084	20	stress.abiotic.heat	stress
III	Bra026084	20	stress.abiotic.heat	stress
III	Bra022083	20	stress.abiotic.heat	stress
III	Bra022084	20	stress.abiotic.heat	stress
III	Bra019231	20	stress.abiotic.heat	stress

III	Bra001103	20	stress.abiotic.cold	stress
III	Bra005018	20	stress.abiotic.drought/salt	stress
III	Bra005018	20	stress.abiotic.drought/salt	stress
III	Bra016362	20	stress.abiotic.unspecified	stress
III	Bra016362	20	stress.abiotic.unspecified	stress
III	Bra016178	20	stress.abiotic.unspecified	stress
III	Bra003809	23	nucleotide metabolism.synthesis.purine.FGAR amidotransferase	nucleotide_metabolism
III	Bra007342	23	nucleotide metabolism.synthesis.purine.adenylosuccinate synthase	nucleotide_metabolism
III	Bra003597	23	nucleotide metabolism.salvage.NUDIX hydrolases	nucleotide_metabolism
III	Bra019456	27	RNA.processing.degradation dicer	RNA
III	Bra013748	27	RNA.regulation of transcription.ARF, Auxin Response Factor family	RNA
III	Bra037592	27	RNA.regulation of transcription.C2H2 zinc finger family	RNA
III	Bra004660	27	RNA.regulation of transcription.C2H2 zinc finger family	RNA
III	Bra004660	27	RNA.regulation of transcription.C2H2 zinc finger family	RNA
III	Bra010348	27	RNA.regulation of transcription.CPP(Zn),CPP1-related transcription factor family	RNA
III	Bra039340	27	RNA.regulation of transcription.G2-like transcription factor family, GARP	RNA
III	Bra012076	27	RNA.regulation of transcription.MYB domain transcription factor family	RNA
III	Bra006853	27	RNA.regulation of transcription.MYB-related transcription factor family	RNA
III	Bra040010	27	RNA.regulation of transcription.Trihelix, Triple-Helix transcription factor family	RNA
III	Bra040010	27	RNA.regulation of transcription.Trihelix, Triple-Helix transcription factor family	RNA
III	Bra036731	27	RNA.regulation of transcription.Trihelix, Triple-Helix transcription factor family	RNA
III	Bra000719	27	RNA.regulation of transcription.Global transcription factor group	RNA
III	Bra035943	27	RNA.regulation of transcription.HDA	RNA
III	Bra035943	27	RNA.regulation of transcription.HDA	RNA
III	Bra015091	27	RNA.regulation of transcription.putative transcription regulator	RNA
III	Bra030860	27	RNA.regulation of transcription.unclassified	RNA
III	Bra017269	27	RNA.regulation of transcription.unclassified	RNA
III	Bra011081	29	protein.aa activation.aspartate-tRNA ligase	protein
III	Bra024759	29	protein.aa activation.glutamine-tRNA ligase	protein
III	Bra026449	29	protein.aa activation.arginine-tRNA ligase	protein
III	Bra014242	29	protein.aa activation.alanine-tRNA ligase	protein
III	Bra018836	29	protein.aa activation.alanine-tRNA ligase	protein
III	Bra040982	29	protein.synthesis.ribosomal protein.prokaryotic.chloroplast.50S subunit.L2	protein
III	Bra040982	29	protein.synthesis.ribosomal protein.prokaryotic.chloroplast.50S subunit.L2	protein
III	Bra025989	29	protein.synthesis.initiation	protein
III	Bra020136	29	protein.synthesis.initiation	protein
III	Bra020136	29	protein.synthesis.initiation	protein
III	Bra027021	29	protein.synthesis.elongation	protein
III	Bra027021	29	protein.synthesis.elongation	protein
III	Bra038335	29	protein.targeting.nucleus	protein
III	Bra026183	29	protein.targeting.nucleus	protein
III	Bra006519	29	protein.targeting.nucleus	protein
III	Bra032818	29	protein.targeting.nucleus	protein
III	Bra039151	29	protein.targeting.mitochondria	protein
III	Bra015538	29	protein.targeting.chloroplast	protein
III	Bra019112	29	protein.degradation.subtilases	protein
III	Bra028274	29	protein.degradation.subtilases	protein
III	Bra038463	29	protein.degradation.ubiquitin.E3.RING	protein
III	Bra009367	29	protein.degradation.ubiquitin.E3.RING	protein
III	Bra028247	29	protein.degradation.ubiquitin.E3.SCF.FBOX	protein
III	Bra015193	29	protein.degradation.ubiquitin.E3.SCF.FBOX	protein
III	Bra025046	29	protein.degradation.cysteine protease	protein

III	Bra024230	29	protein.degradation.cysteine protease	protein
III	Bra035651	29	protein.degradation.metalloprotease	protein
III	Bra012952	30	signalling.receptor kinases.leucine rich repeat XI	signalling
III	Bra038911	30	signalling.receptor kinases.leucine rich repeat XI	signalling
III	Bra038911	30	signalling.receptor kinases.leucine rich repeat XI	signalling
III	Bra018492	30	signalling.receptor kinases.DUF 26	signalling
III	Bra032332	30	signalling.receptor kinases.DUF 26	signalling
III	Bra040804	30	signalling.receptor kinases.wall associated kinase	signalling
III	Bra014140	30	signalling.receptor kinases.leucine rich repeat III	signalling
III	Bra020790	30	signalling.receptor kinases.misc	signalling
III	Bra006858	34	transport.hormones.auxin	transport
IV	Bra018298	3	minor CHO metabolism.callose	minor_CHOmetabolism
IV	Bra036638	10	cell wall.modification	cell_wall
IV	Bra036225	10	cell wall.modification	cell_wall
IV	Bra009441	14	S-assimilation.sulfite redox	S-assimilation
IV	Bra014981	15	metal handling	metal_handling
IV	Bra007008	15	metal handling.binding, chelation and storage	metal_handling
IV	Bra004996	16	secondary metabolism.phenylpropanoids	secondary_metabolism
IV	Bra029999	16	secondary metabolism.phenylpropanoids	secondary_metabolism
IV	Bra020412	16	secondary metabolism.wax	secondary_metabolism
IV	Bra026469	20	stress.biotic	stress
IV	Bra026058	26	misc.cytochrome P450	misc_otherPhosphate
IV	Bra026058	26	misc.cytochrome P450	misc_otherPhosphate
IV	Bra015209	26	misc.oxygenases	misc_otherPhosphate
IV	Bra003636	26	misc.myrosinases-lectin-jacalin	misc_otherPhosphate
IV	Bra018449	26	misc.dynamin	misc_otherPhosphate
IV	Bra014453	26	misc.UDP glucosyl and glucoronyl transferases	misc_otherPhosphate
IV	Bra007573	26	misc.UDP glucosyl and glucoronyl transferases	misc_otherPhosphate
IV	Bra003318	26	misc.beta 1,3 glucan hydrolases	misc_otherPhosphate
IV	Bra013452	27	RNA.transcription	RNA
IV	Bra009485	28	DNA.synthesis/chromatin structure	DNA
IV	Bra011260	28	DNA.repair	DNA
IV	Bra016689	28	DNA.unspecified	DNA
IV	Bra018709	29	protein.postranslational modification	protein
IV	Bra010996	29	protein.postranslational modification	protein
IV	Bra009045	29	protein.postranslational modification	protein
IV	Bra025165	29	protein.postranslational modification	protein
IV	Bra014141	29	protein.postranslational modification	protein
IV	Bra021685	29	protein.postranslational modification	protein
IV	Bra029095	29	protein.postranslational modification	protein
IV	Bra011411	29	protein.postranslational modification	protein
IV	Bra020994	29	protein.postranslational modification	protein
IV	Bra021498	29	protein.postranslational modification	protein
IV	Bra027409	29	protein.postranslational modification	protein
IV	Bra003844	29	protein.postranslational modification	protein
IV	Bra033411	29	protein.postranslational modification	protein
IV	Bra019550	29	protein.postranslational modification	protein
IV	Bra040171	29	protein.postranslational modification	protein
IV	Bra009331	29	protein.degradation	protein
IV	Bra037730	29	protein.degradation	protein
IV	Bra037661	29	protein.degradation	protein
IV	Bra039955	29	protein.folding	protein

IV	Bra005514	29	protein.folding	protein
IV	Bra019081	29	protein.glycosylation	protein
IV	Bra001866	30	signalling.light	signalling
IV	Bra011001	30	signalling.light	signalling
IV	Bra039947	30	signalling.light	signalling
IV	Bra018236	30	signalling.calcium	signalling
IV	Bra038129	30	signalling.calcium	signalling
IV	Bra038129	30	signalling.calcium	signalling
IV	Bra003287	30	signalling.calcium	signalling
IV	Bra003287	30	signalling.calcium	signalling
IV	Bra011605	30	signalling.calcium	signalling
IV	Bra007216	30	signalling.G-proteins	signalling
IV	Bra007216	30	signalling.G-proteins	signalling
IV	Bra003046	30	signalling.G-proteins	signalling
IV	Bra025243	31	cell.organisation	cell
IV	Bra005575	31	cell.organisation	cell
IV	Bra023686	31	cell.organisation	cell
IV	Bra021066	31	cell.organisation	cell
IV	Bra002114	31	cell.organisation	cell
IV	Bra023018	31	cell.organisation	cell
IV	Bra006504	31	cell.division	cell
IV	Bra023606	31	cell.cycle	cell
IV	Bra017009	31	cell.vesicle transport	cell
IV	Bra027335	31	cell.vesicle transport	cell
IV	Bra011325	31	cell.vesicle transport	cell
IV	Bra001286	31	cell.vesicle transport	cell
IV	Bra018644	31	cell.vesicle transport	cell
IV	Bra011849	31	cell.vesicle transport	cell
IV	Bra021150	31	cell.vesicle transport	cell
IV	Bra038070	31	cell.vesicle transport	cell
IV	Bra024005	31	cell.vesicle transport	cell
IV	Bra004363	33	development.squamosa promoter binding like (SPL)	development
IV	Bra015732	33	development.squamosa promoter binding like (SPL)	development
IV	Bra015732	33	development.squamosa promoter binding like (SPL)	development
IV	Bra023522	33	development.unspecified	development
IV	Bra015179	33	development.unspecified	development
IV	Bra031088	33	development.unspecified	development
IV	Bra031085	33	development.unspecified	development
IV	Bra031085	33	development.unspecified	development
IV	Bra013936	34	transport.ABC transporters and multidrug resistance systems	transport
IV	Bra039457	34	transport.sugars	transport
IV	Bra003534	34	transport.sugars	transport
IV	Bra039441	34	transport.sugars	transport
IV	Bra019686	34	transport.phosphate	transport
IV	Bra019686	34	transport.phosphate	transport
IV	Bra030999	34	transport.misc	transport
IV	Bra008012	34	transport.misc	transport
IV	Bra013489	35	not assigned.unknown	not_assigned
IV	Bra017845	35	not assigned.unknown	not_assigned
IV	Bra014441	35	not assigned.unknown	not_assigned
IV	Bra027091	35	not assigned.unknown	not_assigned
IV	Bra013605	35	not assigned.unknown	not_assigned

IV	Bra038296	35	not assigned.unknown	not_assigned
IV	Bra025137	35	not assigned.unknown	not_assigned
IV	Bra003296	35	not assigned.unknown	not_assigned
IV	Bra015238	35	not assigned.unknown	not_assigned
IV	Bra030480	35	not assigned.unknown	not_assigned
IV	Bra007942	35	not assigned.unknown	not_assigned
IV	Bra014810	35	not assigned.unknown	not_assigned
IV	Bra029796	35	not assigned.unknown	not_assigned
IV	Bra026901	35	not assigned.unknown	not_assigned
IV	Bra025396	35	not assigned.unknown	not_assigned
IV	Bra001263	35	not assigned.unknown	not_assigned
IV	Bra003285	35	not assigned.unknown	not_assigned
IV	Bra020186	35	not assigned.unknown	not_assigned
IV	Bra039410	35	not assigned.unknown	not_assigned
IV	Bra006159	35	not assigned.unknown	not_assigned
IV	Bra036132	35	not assigned.unknown	not_assigned
IV	Bra007808	35	not assigned.unknown	not_assigned
IV	Bra022916	35	not assigned.unknown	not_assigned
IV	Bra006821	35	not assigned.unknown	not_assigned
IV	Bra022501	35	not assigned.unknown	not_assigned
IV	Bra034457	35	not assigned.unknown	not_assigned
IV	Bra037496	35	not assigned.unknown	not_assigned
IV	Bra004749	35	not assigned.unknown	not_assigned
IV	Bra032518	35	not assigned.unknown	not_assigned
IV	Bra009799	35	not assigned.unknown	not_assigned
IV	Bra016792	35	not assigned.unknown	not_assigned
IV	Bra016100	35	not assigned.unknown	not_assigned
IV	Bra024715	35	not assigned.unknown	not_assigned
IV	Bra033544	35	not assigned.unknown	not_assigned
IV	Bra018720	35	not assigned.unknown	not_assigned
IV	Bra003464	35	not assigned.unknown	not_assigned
IV	Bra036092	35	not assigned.unknown	not_assigned
IV	Bra028676	35	not assigned.unknown	not_assigned
IV	Bra009065	35	not assigned.unknown	not_assigned
IV	Bra019778	35	not assigned.unknown	not_assigned
IV	Bra006293	35	not assigned.unknown	not_assigned
IV	Bra012734	35	not assigned.unknown	not_assigned
IV	Bra040764	35	not assigned.unknown	not_assigned
IV	Bra026104	35	not assigned.unknown	not_assigned
IV	Bra004313	35	not assigned.unknown	not_assigned
IV	Bra037058	35	not assigned.unknown	not_assigned
IV	Bra004645	35	not assigned.unknown	not_assigned
IV	Bra018568	35	not assigned.unknown	not_assigned
IV	Bra018568	35	not assigned.unknown	not_assigned
IV	Bra036779	35	not assigned.unknown	not_assigned
IV	Bra002288	35	not assigned.unknown	not_assigned
IV	Bra023724	35	not assigned.unknown	not_assigned
IV	Bra024703	35	not assigned.unknown	not_assigned
IV	Bra006551	35	not assigned.unknown	not_assigned
IV	Bra018664	35	not assigned.unknown	not_assigned
IV	Bra017050	35	not assigned.unknown	not_assigned
IV	Bra037058	35	not assigned.unknown	not_assigned

IV	Bra001409	35	not assigned.unknown	not_assigned
IV	Bra032537	35	not assigned.unknown	not_assigned
IV	Bra002013	35	not assigned.unknown	not_assigned
IV	Bra027884	35	not assigned.unknown	not_assigned
IV	Bra033425	35	not assigned.unknown	not_assigned
IV	Bra032054	35	not assigned.unknown	not_assigned
IV	Bra041152	35	not assigned.unknown	not_assigned
IV	Bra009363	1	PS.lightreaction.ATP synthase.beta subunit	Photosynthesis
IV	Bra032110	1	PS.calvin cycle.GAP	Photosynthesis
IV	Bra005845	10	cell wall.cellulose synthesis.cellulose synthase	cell_wall
IV	Bra028768	10	cell wall.cellulose synthesis.cellulose synthase	cell_wall
IV	Bra034817	10	cell wall.cell wall proteins.AGPs.AGP	cell_wall
IV	Bra024571	10	cell wall.degradation.cellulases and beta -1,4-glucanases	cell_wall
IV	Bra018559	10	cell wall.degradation.cellulases and beta -1,4-glucanases	cell_wall
IV	Bra040790	10	cell wall.degradation.pectate lyases and polygalacturonases	cell_wall
IV	Bra006031	10	cell wall.pectin*esterases.PME	cell_wall
IV	Bra022648	10	cell wall.pectin*esterases.PME	cell_wall
IV	Bra009401	10	cell wall.pectin*esterases.PME	cell_wall
IV	Bra004560	11	lipid metabolism."exotics" (steroids, squalene etc).sphingolipids	lipid_metabolism
IV	Bra011076	11	lipid metabolism.lipid degradation.beta-oxidation.multifunctional	lipid_metabolism
IV	Bra011076	11	lipid metabolism.lipid degradation.beta-oxidation.multifunctional	lipid_metabolism
IV	Bra001020	13	amino acid metabolism.synthesis.aspartate family.methionine.cystathionine gamma-synthase	amino_acid_metabolism
IV	Bra013009	13	amino acid metabolism.synthesis.branched chain group.leucine specific.2-isopropylmalate synthase	amino_acid_metabolism
IV	Bra013009	13	amino acid metabolism.synthesis.branched chain group.leucine specific.2-isopropylmalate synthase	amino_acid_metabolism
IV	Bra025421	13	amino acid metabolism.degradation.glutamate family.proline	amino_acid_metabolism
IV	Bra025421	13	amino acid metabolism.degradation.glutamate family.proline	amino_acid_metabolism
IV	Bra016810	13	amino acid metabolism.degradation.serine-glycine-cysteine group.glycine	amino_acid_metabolism
IV	Bra012513	16	secondary metabolism.isoprenoids.terpenoids	secondary_metabolism
IV	Bra032359	17	hormone metabolism.abscisic acid.synthesis-degradation.synthesis.9-cis-epoxycarotenoid dioxygenase	hormone_metabolism
IV	Bra005925	17	hormone metabolism.auxin.signal transduction	hormone_metabolism
IV	Bra026586	17	hormone metabolism.auxin.signal transduction	hormone_metabolism
IV	Bra022984	17	hormone metabolism.auxin.induced-regulated-responsive-activated	hormone_metabolism
IV	Bra038233	17	hormone metabolism.brassinosteroid.synthesis-degradation.sterols.DWF1	hormone_metabolism
IV	Bra032779	17	hormone metabolism.brassinosteroid.synthesis-degradation.sterols.other	hormone_metabolism
IV	Bra006559	17	hormone metabolism.ethylene.synthesis-degradation	hormone_metabolism
IV	Bra009771	17	hormone metabolism.ethylene.synthesis-degradation	hormone_metabolism
IV	Bra009771	17	hormone metabolism.ethylene.synthesis-degradation	hormone_metabolism
IV	Bra026757	17	hormone metabolism.ethylene.synthesis-degradation	hormone_metabolism
IV	Bra000848	17	hormone metabolism.gibberelin.synthesis-degradation	hormone_metabolism
IV	Bra013691	20	stress.biotic.PR-proteins	stress
IV	Bra019305	20	stress.biotic.PR-proteins	stress
IV	Bra022069	20	stress.biotic.PR-proteins	stress
IV	Bra022071	20	stress.biotic.PR-proteins	stress
IV	Bra008143	20	stress.abiotic.heat	stress
IV	Bra010498	20	stress.abiotic.heat	stress
IV	Bra022080	20	stress.abiotic.heat	stress
IV	Bra009548	20	stress.abiotic.heat	stress
IV	Bra040740	20	stress.abiotic.heat	stress
IV	Bra013282	20	stress.abiotic.drought/salt	stress

IV	Bra030140	20	stress.abiotic.drought/salt	stress
IV	Bra010830	20	stress.abiotic.drought/salt	stress
IV	Bra030140	20	stress.abiotic.drought/salt	stress
IV	Bra032319	20	stress.abiotic.drought/salt	stress
IV	Bra012363	20	stress.abiotic.unspecified	stress
IV	Bra009669	23	nucleotide metabolism.synthesis.pyrimidine.dihydroorotate dehydrogenase	nucleotide_metabolism
IV	Bra003204	23	nucleotide metabolism.synthesis.purine.AIR synthase	nucleotide_metabolism
IV	Bra018407	27	RNA.processing.splicing	RNA
IV	Bra021701	27	RNA.processing.splicing	RNA
IV	Bra012034	27	RNA.regulation of transcription.C2H2 zinc finger family	RNA
IV	Bra032328	27	RNA.regulation of transcription.C2H2 zinc finger family	RNA
IV	Bra020491	27	RNA.regulation of transcription.HB,Homeobox transcription factor family	RNA
IV	Bra032908	27	RNA.regulation of transcription.HB,Homeobox transcription factor family	RNA
IV	Bra026543	27	RNA.regulation of transcription.MADS box transcription factor family	RNA
IV	Bra029762	27	RNA.regulation of transcription.MYB domain transcription factor family	RNA
IV	Bra035547	27	RNA.regulation of transcription.MYB domain transcription factor family	RNA
IV	Bra041096	27	RNA.regulation of transcription.MYB domain transcription factor family	RNA
IV	Bra035547	27	RNA.regulation of transcription.MYB domain transcription factor family	RNA
IV	Bra019993	27	RNA.regulation of transcription.MYB-related transcription factor family	RNA
IV	Bra034866	27	RNA.regulation of transcription.Trihelix, Triple-Helix transcription factor family	RNA
IV	Bra010231	27	RNA.regulation of transcription.WRKY domain transcription factor family	RNA
IV	Bra025021	27	RNA.regulation of transcription.WRKY domain transcription factor family	RNA
IV	Bra028713	27	RNA.regulation of transcription.bZIP transcription factor family	RNA
IV	Bra007380	27	RNA.regulation of transcription.bZIP transcription factor family	RNA
IV	Bra007380	27	RNA.regulation of transcription.bZIP transcription factor family	RNA
IV	Bra004908	27	RNA.regulation of transcription.AS2,Lateral Organ Boundaries Gene Family	RNA
IV	Bra000114	27	RNA.regulation of transcription.Chromatin Remodeling Factors	RNA
IV	Bra040204	27	RNA.regulation of transcription.ARR	RNA
IV	Bra004076	27	RNA.regulation of transcription.ARR	RNA
IV	Bra022183	27	RNA.regulation of transcription.ARR	RNA
IV	Bra004114	27	RNA.regulation of transcription.Global transcription factor group	RNA
IV	Bra007723	27	RNA.regulation of transcription.Methyl binding domain proteins	RNA
IV	Bra011540	27	RNA.regulation of transcription.bHLH,Basic Helix-Loop-Helix family	RNA
IV	Bra027290	27	RNA.regulation of transcription.PHD finger transcription factor	RNA
IV	Bra018043	27	RNA.regulation of transcription.putative transcription regulator	RNA
IV	Bra041065	27	RNA.regulation of transcription.putative transcription regulator	RNA
IV	Bra033142	27	RNA.regulation of transcription.putative transcription regulator	RNA
IV	Bra012929	27	RNA.regulation of transcription.putative transcription regulator	RNA
IV	Bra003521	27	RNA.regulation of transcription.PWWP domain protein	RNA
IV	Bra003521	27	RNA.regulation of transcription.PWWP domain protein	RNA
IV	Bra039562	27	RNA.regulation of transcription.SET-domain transcriptional regulator family	RNA
IV	Bra028701	27	RNA.regulation of transcription.Zn-finger(CCHC)	RNA
IV	Bra010136	27	RNA.regulation of transcription.C2C2(Zn) DOF zinc finger family	RNA
IV	Bra019118	27	RNA.regulation of transcription.C2C2(Zn) GATA transcription factor family	RNA
IV	Bra007542	27	RNA.regulation of transcription.C2C2(Zn) GATA transcription factor family	RNA
IV	Bra030860	27	RNA.regulation of transcription.unclassified	RNA
IV	Bra033660	27	RNA.regulation of transcription.unclassified	RNA
IV	Bra018059	27	RNA.regulation of transcription.unclassified	RNA
IV	Bra029197	29	protein.aa activation.bifunctional aminoacyl-tRNA synthetase	protein
IV	Bra022488	29	protein.synthesis.initiation	protein
IV	Bra026941	29	protein.synthesis.initiation	protein
IV	Bra003054	29	protein.targeting.nucleus	protein

IV	Bra013228	29	protein.targeting.secretory pathway.ER	protein
IV	Bra039126	29	protein.targeting.secretory pathway.plasma membrane	protein
IV	Bra000962	29	protein.postranslational modification.kinase.receptor like cytoplasmatic kinase VII	protein
IV	Bra035164	29	protein.postranslational modification.kinase.receptor like cytoplasmatic kinase VII	protein
IV	Bra037362	29	protein.postranslational modification.kinase.receptor like cytoplasmatic kinase VII	protein
IV	Bra008680	29	protein.postranslational modification.kinase.receptor like cytoplasmatic kinase VII	protein
IV	Bra037197	29	protein.postranslational modification.kinase.receptor like cytoplasmatic kinase VII	protein
IV	Bra038768	29	protein.degradation.subtilases	protein
IV	Bra027915	29	protein.degradation.aspartate protease	protein
IV	Bra013838	29	protein.degradation.ubiquitin.proteasom	protein
IV	Bra014777	29	protein.degradation.ubiquitin.E3.RING	protein
IV	Bra003450	29	protein.degradation.ubiquitin.E3.RING	protein
IV	Bra027538	29	protein.degradation.ubiquitin.E3.RING	protein
IV	Bra017732	29	protein.degradation.ubiquitin.E3.RING	protein
IV	Bra040564	29	protein.degradation.ubiquitin.E3.RING	protein
IV	Bra027309	29	protein.degradation.ubiquitin.E3.SCF.FBOX	protein
IV	Bra029718	29	protein.degradation.ubiquitin.E3.SCF.FBOX	protein
IV	Bra029718	29	protein.degradation.ubiquitin.E3.SCF.FBOX	protein
IV	Bra007843	29	protein.degradation.ubiquitin.E3.SCF.FBOX	protein
IV	Bra003561	29	protein.degradation.AAA type	protein
IV	Bra007906	3	minor CHO metabolism.trehalose.potential TPS/TPP	minor_CHOmetabolism
IV	Bra019322	30	signalling.receptor kinases.DUF 26	signalling
IV	Bra019855	30	signalling.receptor kinases.DUF 26	signalling
IV	Bra032332	30	signalling.receptor kinases.DUF 26	signalling
IV	Bra019866	30	signalling.receptor kinases.DUF 26	signalling
IV	Bra040315	30	signalling.receptor kinases.DUF 26	signalling
IV	Bra033940	30	signalling.receptor kinases.leucine rich repeat III	signalling
IV	Bra024897	30	signalling.receptor kinases.leucine rich repeat III	signalling
IV	Bra001255	30	signalling.receptor kinases.leucine rich repeat III	signalling
IV	Bra010508	8	TCA / org. transformation.TCA.aconitase	TCA
IV	Bra010508	8	TCA / org. transformation.TCA.aconitase	TCA
V	Bra018090	5	fermentation.aldehyde dehydrogenase	fermentation
V	Bra019846	10	cell wall.modification	cell_wall
V	Bra029719	11	lipid metabolism.lipid transfer proteins etc	lipid_metabolism
V	Bra038908	11	lipid metabolism.lipid transfer proteins etc	lipid_metabolism
V	Bra038908	11	lipid metabolism.lipid transfer proteins etc	lipid_metabolism
V	Bra003693	13	amino acid metabolism.misc	amino_acid_metabolism
V	Bra002630	15	metal handling	metal_handling
V	Bra032756	15	metal handling	metal_handling
V	Bra038965	15	metal handling	metal_handling
V	Bra038965	15	metal handling	metal_handling
V	Bra035839	15	metal handling.binding, chelation and storage	metal_handling
V	Bra029388	16	secondary metabolism.phenylpropanoids	secondary_metabolism
V	Bra029388	16	secondary metabolism.phenylpropanoids	secondary_metabolism
V	Bra009334	19	tetrapyrrole synthesis.porphobilinogen deaminase	tetrapyrrole_synthesis
V	Bra010539	20	stress.biotic	stress
V	Bra012293	21	redox.thioredoxin	redox
V	Bra004455	21	redox.thioredoxin	redox
V	Bra019071	21	redox.thioredoxin	redox
V	Bra012293	21	redox.thioredoxin	redox
V	Bra019571	21	redox.ascorbate and glutathione	redox

V	Bra034763	25	C1-metabolism.Methylenetetrahydrofolate dehydrogenase & Methenyltetrahydrofolate cyclohydrolase	C1-metabolism
V	Bra034763	25	C1-metabolism.Methylenetetrahydrofolate dehydrogenase & Methenyltetrahydrofolate cyclohydrolase	C1-metabolism
V	Bra016828	26	misc.cytochrome P450	misc_otherPhosphate
V	Bra016828	26	misc.cytochrome P450	misc_otherPhosphate
V	Bra007831	26	misc.peroxidases	misc_otherPhosphate
V	Bra016682	26	misc.oxygenases	misc_otherPhosphate
V	Bra022161	26	misc.myrosinases-lectin-jacalin	misc_otherPhosphate
V	Bra008558	26	misc.UDP glucosyl and glucoronyl transferases	misc_otherPhosphate
V	Bra032951	26	misc.short chain dehydrogenase/reductase (SDR)	misc_otherPhosphate
V	Bra010820	26	misc.GDSL-motif lipase	misc_otherPhosphate
V	Bra010820	26	misc.GDSL-motif lipase	misc_otherPhosphate
V	Bra004834	26	misc.gluco-, galacto- and mannosidases	misc_otherPhosphate
V	Bra015719	26	misc.O-methyl transferases	misc_otherPhosphate
V	Bra013582	26	misc.oxidases - copper, flavone etc.	misc_otherPhosphate
V	Bra000476	26	misc.nitrilases, *nitrile lyases, berberine bridge enzymes, reticuline oxidases, troponine reductases	misc_otherPhosphate
V	Bra000476	26	misc.nitrilases, *nitrile lyases, berberine bridge enzymes, reticuline oxidases, troponine reductases	misc_otherPhosphate
V	Bra003645	26	misc.glutathione S transferases	misc_otherPhosphate
V	Bra003644	26	misc.glutathione S transferases	misc_otherPhosphate
V	Bra016686	27	RNA.regulation of transcription	RNA
V	Bra001111	27	RNA.RNA binding	RNA
V	Bra010757	27	RNA.RNA binding	RNA
V	Bra030436	27	RNA.RNA binding	RNA
V	Bra011869	27	RNA.RNA binding	RNA
V	Bra012275	27	RNA.RNA binding	RNA
V	Bra021218	29	protein.postranslational modification	protein
V	Bra033332	29	protein.degradation	protein
V	Bra006338	29	protein.folding	protein
V	Bra026581	30	signalling.calcium	signalling
V	Bra017707	30	signalling.calcium	signalling
V	Bra014671	30	signalling.calcium	signalling
V	Bra004620	30	signalling.calcium	signalling
V	Bra027043	30	signalling.G-proteins	signalling
V	Bra036819	30	signalling.G-proteins	signalling
V	Bra022360	30	signalling.G-proteins	signalling
V	Bra022360	30	signalling.G-proteins	signalling
V	Bra016570	30	signalling.G-proteins	signalling
V	Bra030627	30	signalling.14-3-3 proteins	signalling
V	Bra000268	30	signalling.14-3-3 proteins	signalling
V	Bra008771	31	cell.organisation	cell
V	Bra006517	31	cell.organisation	cell
V	Bra018184	31	cell.organisation	cell
V	Bra010114	31	cell.organisation	cell
V	Bra018184	31	cell.organisation	cell
V	Bra014946	31	cell.organisation	cell
V	Bra004888	31	cell.cycle	cell
V	Bra002054	31	cell.vesicle transport	cell
V	Bra002054	31	cell.vesicle transport	cell
V	Bra034085	31	cell.vesicle transport	cell
V	Bra006100	31	cell.vesicle transport	cell

V	Bra036702	31	cell.vesicle transport	cell
V	Bra035723	33	development.unspecified	development
V	Bra031808	33	development.unspecified	development
V	Bra003751	33	development.unspecified	development
V	Bra010499	33	development.unspecified	development
V	Bra008723	34	transport.sugars	transport
V	Bra027468	35	not assigned.unknown	not_assigned
V	Bra032559	35	not assigned.unknown	not_assigned
V	Bra026992	35	not assigned.unknown	not_assigned
V	Bra039829	35	not assigned.unknown	not_assigned
V	Bra038480	35	not assigned.unknown	not_assigned
V	Bra011697	35	not assigned.unknown	not_assigned
V	Bra011697	35	not assigned.unknown	not_assigned
V	Bra039935	35	not assigned.unknown	not_assigned
V	Bra016462	35	not assigned.unknown	not_assigned
V	Bra031139	35	not assigned.unknown	not_assigned
V	Bra003304	35	not assigned.unknown	not_assigned
V	Bra018217	35	not assigned.unknown	not_assigned
V	Bra028172	35	not assigned.unknown	not_assigned
V	Bra039595	35	not assigned.unknown	not_assigned
V	Bra019704	35	not assigned.unknown	not_assigned
V	Bra024305	35	not assigned.unknown	not_assigned
V	Bra007807	35	not assigned.unknown	not_assigned
V	Bra001930	35	not assigned.unknown	not_assigned
V	Bra015007	35	not assigned.unknown	not_assigned
V	Bra034717	35	not assigned.unknown	not_assigned
V	Bra013502	35	not assigned.unknown	not_assigned
V	Bra008770	35	not assigned.unknown	not_assigned
V	Bra007788	35	not assigned.unknown	not_assigned
V	Bra037889	35	not assigned.unknown	not_assigned
V	Bra004133	35	not assigned.unknown	not_assigned
V	Bra039375	35	not assigned.unknown	not_assigned
V	Bra038063	35	not assigned.unknown	not_assigned
V	Bra038630	35	not assigned.unknown	not_assigned
V	Bra011328	35	not assigned.unknown	not_assigned
V	Bra032569	35	not assigned.unknown	not_assigned
V	Bra012716	35	not assigned.unknown	not_assigned
V	Bra013156	35	not assigned.unknown	not_assigned
V	Bra029687	35	not assigned.unknown	not_assigned
V	Bra028864	35	not assigned.unknown	not_assigned
V	Bra013794	35	not assigned.unknown	not_assigned
V	Bra023161	35	not assigned.unknown	not_assigned
V	Bra010769	35	not assigned.unknown	not_assigned
V	Bra032749	1	PS.photorespiration.serine hydroxymethyltransferase	Photosynthesis
V	Bra022129	1	PS.photorespiration.serine hydroxymethyltransferase	Photosynthesis
V	Bra022129	1	PS.photorespiration.serine hydroxymethyltransferase	Photosynthesis
V	Bra033439	10	cell wall.cell wall proteins.AGPs.AGP	cell_wall
V	Bra009854	10	cell wall.degradation.pectate lyases and polygalacturonases	cell_wall
V	Bra019912	10	cell wall.degradation.pectate lyases and polygalacturonases	cell_wall
V	Bra019912	10	cell wall.degradation.pectate lyases and polygalacturonases	cell_wall
V	Bra013734	10	cell wall.degradation.pectate lyases and polygalacturonases	cell_wall
V	Bra006976	10	cell wall.degradation.pectate lyases and polygalacturonases	cell_wall

V	Bra006976	10	cell wall.degradation.pectate lyases and polygalacturonases	cell_wall
V	Bra007609	10	cell wall.degradation.pectate lyases and polygalacturonases	cell_wall
V	Bra035852	10	cell wall.degradation.pectate lyases and polygalacturonases	cell_wall
V	Bra019264	10	cell wall.degradation.pectate lyases and polygalacturonases	cell_wall
V	Bra028786	11	lipid metabolism.FA synthesis and FA elongation.beta ketoacyl CoA synthase	lipid_metabolism
V	Bra011235	11	lipid metabolism.FA desaturation.omega 6 desaturase	lipid_metabolism
V	Bra007154	11	lipid metabolism."exotics" (steroids, squalene etc).trans-2-enoyl-CoA reductase (NADPH)	lipid_metabolism
V	Bra026522	11	lipid metabolism."exotics" (steroids, squalene etc).sphingolipids	lipid_metabolism
V	Bra028132	12	N-metabolism.ammonia metabolism.glutamine synthase	N-metabolism
V	Bra028132	12	N-metabolism.ammonia metabolism.glutamine synthase	N-metabolism
V	Bra008612	12	N-metabolism.ammonia metabolism.glutamine synthase	N-metabolism
V	Bra003758	13	amino acid metabolism.synthesis.glutamate family.arginine.ornithine carbamoyltransferase	amino_acid_metabolism
V	Bra003758	13	amino acid metabolism.synthesis.glutamate family.arginine.ornithine carbamoyltransferase	amino_acid_metabolism
V	Bra021241	13	amino acid metabolism.synthesis.aspartate family.methionine	amino_acid_metabolism
V	Bra021164	13	amino acid metabolism.degradation.aspartate family.asparagine.L-asparaginase	amino_acid_metabolism
V	Bra021164	13	amino acid metabolism.degradation.aspartate family.asparagine.L-asparaginase	amino_acid_metabolism
V	Bra001921	13	amino acid metabolism.degradation.aspartate family.methionine	amino_acid_metabolism
V	Bra020673	13	amino acid metabolism.degradation.branched-chain group.shared	amino_acid_metabolism
V	Bra010219	13	amino acid metabolism.degradation.aromatic aa.tryptophan	amino_acid_metabolism
V	Bra031666	16	secondary metabolism.flavonoids.dihydroflavonols	secondary_metabolism
V	Bra025748	16	secondary metabolism.flavonoids.isoflavones.isoflavone reductase	secondary_metabolism
V	Bra026044	17	hormone metabolism.auxin.induced-regulated-responsive-activated	hormone_metabolism
V	Bra022459	17	hormone metabolism.brassinosteroid.synthesis-degradation.sterols.DWF1	hormone_metabolism
V	Bra008271	17	hormone metabolism.jasmonate.synthesis-degradation.12-Oxo-PDA-reductase	hormone_metabolism
V	Bra008271	17	hormone metabolism.jasmonate.synthesis-degradation.12-Oxo-PDA-reductase	hormone_metabolism
V	Bra033152	20	stress.biotic.PR-proteins	stress
V	Bra037908	20	stress.abiotic.heat	stress
V	Bra005438	20	stress.abiotic.drought/salt	stress
V	Bra021032	20	stress.abiotic.unspecified	stress
V	Bra000654	20	stress.abiotic.unspecified	stress
V	Bra030083	20	stress.abiotic.unspecified	stress
V	Bra024995	20	stress.abiotic.unspecified	stress
V	Bra003186	23	nucleotide metabolism.synthesis.pyrimidine.orotate phosphoribosyltransferase	nucleotide_metabolism
V	Bra015166	26	misc.beta 1,3 glucan hydrolases.glucan endo-1,3-beta-glucosidase	misc_otherPhosphate
V	Bra028476	27	RNA.processing.splicing	RNA
V	Bra032082	27	RNA.processing.splicing	RNA
V	Bra019595	27	RNA.processing.splicing	RNA
V	Bra012044	27	RNA.processing.ribonucleases	RNA
V	Bra001788	27	RNA.processing.ribonucleases	RNA
V	Bra027620	27	RNA.regulation of transcription.GRAS transcription factor family	RNA
V	Bra032394	27	RNA.regulation of transcription.HB,Homeobox transcription factor family	RNA
V	Bra013406	27	RNA.regulation of transcription.HSF,Heat-shock transcription factor family	RNA
V	Bra028599	27	RNA.regulation of transcription.MADS box transcription factor family	RNA
V	Bra039650	27	RNA.regulation of transcription.MYB-related transcription factor family	RNA
V	Bra022164	27	RNA.regulation of transcription.Aux/IAA family	RNA
V	Bra024192	27	RNA.regulation of transcription.Chromatin Remodeling Factors	RNA
V	Bra036165	27	RNA.regulation of transcription.FHA transcription factor	RNA
V	Bra013731	27	RNA.regulation of transcription.High mobility group (HMG) family	RNA
V	Bra011751	27	RNA.regulation of transcription.Histone acetyltransferases	RNA
V	Bra006286	27	RNA.regulation of transcription.bHLH,Basic Helix-Loop-Helix family	RNA

V	Bra027131	27	RNA.regulation of transcription.putative transcription regulator	RNA
V	Bra002986	27	RNA.regulation of transcription.C2C2(Zn) CO-like, Constans-like zinc finger family	RNA
V	Bra019058	27	RNA.regulation of transcription.C2C2(Zn) CO-like, Constans-like zinc finger family	RNA
V	Bra019058	27	RNA.regulation of transcription.C2C2(Zn) CO-like, Constans-like zinc finger family	RNA
V	Bra026361	27	RNA.regulation of transcription.C2C2(Zn) CO-like, Constans-like zinc finger family	RNA
V	Bra012882	27	RNA.regulation of transcription.zf-HD	RNA
V	Bra012882	27	RNA.regulation of transcription.zf-HD	RNA
V	Bra036459	27	RNA.regulation of transcription.unclassified	RNA
V	Bra021882	27	RNA.regulation of transcription.unclassified	RNA
V	Bra038267	28	DNA.synthesis/chromatin structure.histone	DNA
V	Bra037813	28	DNA.synthesis/chromatin structure.histone	DNA
V	Bra006688	28	DNA.synthesis/chromatin structure.histone	DNA
V	Bra011929	28	DNA.synthesis/chromatin structure.histone	DNA
V	Bra009949	28	DNA.synthesis/chromatin structure.histone	DNA
V	Bra006699	28	DNA.synthesis/chromatin structure.histone	DNA
V	Bra038268	28	DNA.synthesis/chromatin structure.histone	DNA
V	Bra011664	29	protein.synthesis.ribosomal protein.prokaryotic.chloroplast.50S subunit.L2	protein
V	Bra024481	29	protein.synthesis.ribosomal protein.prokaryotic.chloroplast.50S subunit.L2	protein
V	Bra029112	29	protein.synthesis.ribosomal protein.eukaryotic.40S subunit.S10	protein
V	Bra022851	29	protein.synthesis.ribosomal protein.eukaryotic.40S subunit.S3	protein
V	Bra001127	29	protein.synthesis.ribosomal protein.eukaryotic.40S subunit.S3A	protein
V	Bra001127	29	protein.synthesis.ribosomal protein.eukaryotic.40S subunit.S3A	protein
V	Bra011551	29	protein.synthesis.ribosomal protein.eukaryotic.40S subunit.S3A	protein
V	Bra040119	29	protein.synthesis.ribosomal protein.eukaryotic.40S subunit.S3A	protein
V	Bra023511	29	protein.synthesis.ribosomal protein.eukaryotic.40S subunit.S9	protein
V	Bra039782	29	protein.synthesis.ribosomal protein.eukaryotic.60S subunit.L12	protein
V	Bra039787	29	protein.synthesis.ribosomal protein.eukaryotic.60S subunit.L12	protein
V	Bra001156	29	protein.synthesis.ribosomal protein.eukaryotic.60S subunit.L18	protein
V	Bra001156	29	protein.synthesis.ribosomal protein.eukaryotic.60S subunit.L18	protein
V	Bra031094	29	protein.synthesis.ribosomal protein.eukaryotic.60S subunit.L31	protein
V	Bra037493	29	protein.synthesis.ribosomal protein.eukaryotic.60S subunit.L5	protein
V	Bra013239	29	protein.synthesis.ribosomal protein.eukaryotic.60S subunit.L13A	protein
V	Bra005038	29	protein.synthesis.ribosomal protein.eukaryotic.60S subunit.L23A	protein
V	Bra003583	29	protein.synthesis.ribosomal protein.eukaryotic.60S subunit.L7	protein
V	Bra014334	29	protein.synthesis.ribosomal protein.eukaryotic.60S subunit.L7	protein
V	Bra014334	29	protein.synthesis.ribosomal protein.eukaryotic.60S subunit.L7	protein
V	Bra017461	29	protein.synthesis.ribosomal protein.eukaryotic.60S subunit.L7	protein
V	Bra001324	29	protein.synthesis.ribosomal protein.eukaryotic.60S subunit.P0	protein
V	Bra001324	29	protein.synthesis.ribosomal protein.eukaryotic.60S subunit.P0	protein
V	Bra029752	29	protein.synthesis.ribosomal protein.eukaryotic.60S subunit.P0	protein
V	Bra002741	29	protein.synthesis.ribosomal protein.eukaryotic.60S subunit.P3	protein
V	Bra004970	29	protein.synthesis.initiation	protein
V	Bra034554	29	protein.synthesis.initiation	protein
V	Bra034554	29	protein.synthesis.initiation	protein
V	Bra015153	29	protein.synthesis.initiation	protein
V	Bra015153	29	protein.synthesis.initiation	protein
V	Bra018764	29	protein.synthesis.ribosomal RNA	protein
V	Bra008429	29	protein.synthesis.misc	protein
V	Bra008429	29	protein.synthesis.misc	protein
V	Bra007224	29	protein.targeting.secretory pathway.unspecified	protein
V	Bra003231	29	protein.targeting.secretory pathway.unspecified	protein
V	Bra024650	29	protein.postranslational modification.kinase.receptor like cytoplasmatic kinase VII	protein

V	Bra026677	29	protein.degradation.ubiquitin	protein
V	Bra013848	29	protein.degradation.ubiquitin.E1	protein
V	Bra013838	29	protein.degradation.ubiquitin.proteasom	protein
V	Bra006775	29	protein.degradation.ubiquitin.proteasom	protein
V	Bra007565	29	protein.degradation.ubiquitin.proteasom	protein
V	Bra033393	29	protein.degradation.ubiquitin.E3.RING	protein
V	Bra040515	29	protein.degradation.ubiquitin.E3.SCF.FBOX	protein
V	Bra016447	29	protein.degradation.cysteine protease	protein
V	Bra004719	30	signalling.receptor kinases.leucine rich repeat XI	signalling
V	Bra023925	30	signalling.receptor kinases.leucine rich repeat XI	signalling
V	Bra004378	31	cell.division.plastid	cell
V	Bra017632	34	transport.p- and v-ATPases.H+-transporting two-sector ATPase	transport
V	Bra006650	34	transport.Major Intrinsic Proteins.PIP	transport
V	Bra000974	34	transport.Major Intrinsic Proteins.PIP	transport
V	Bra037210	8	TCA / org. transformation.other organic acid transformaitons.malic	TCA
V	Bra007701	9	mitochondrial electron transport / ATP synthesis.NADH-DH.localisation not clear	ATP_synthesis
VI	Bra038649	5	fermentation.ADH	fermentation
VI	Bra030204	6	gluconeogenesis.Malate DH	glyoxylate_cycle
VI	Bra018137	6	gluconeogenesis.Malate DH	glyoxylate_cycle
VI	Bra006198	9	mitochondrial electron transport / ATP synthesis.cytochrome c reductase	ATP_synthesis
VI	Bra000109	9	mitochondrial electron transport / ATP synthesis.F1-ATPase	ATP_synthesis
VI	Bra013231	9	mitochondrial electron transport / ATP synthesis.cytochrome c oxidase	ATP_synthesis
VI	Bra019679	10	cell wall.hemicellulose synthesis	cell_wall
VI	Bra001434	10	cell wall.modification	cell_wall
VI	Bra014981	15	metal handling	metal_handling
VI	Bra017139	16	secondary metabolism.simple phenols	secondary_metabolism
VI	Bra009290	16	secondary metabolism.simple phenols	secondary_metabolism
VI	Bra018328	16	secondary metabolism.simple phenols	secondary_metabolism
VI	Bra009716	16	secondary metabolism.phenylpropanoids	secondary_metabolism
VI	Bra003682	16	secondary metabolism.phenylpropanoids	secondary_metabolism
VI	Bra004996	16	secondary metabolism.phenylpropanoids	secondary_metabolism
VI	Bra019268	16	secondary metabolism.wax	secondary_metabolism
VI	Bra019268	16	secondary metabolism.wax	secondary_metabolism
VI	Bra017133	18	Co-factor and vitamine metabolism	Co-factor_vitamine_metabolism
VI	Bra008772	19	tetrapyrrole synthesis.protoporphyrin IX oxidase	tetrapyrrole_synthesis
VI	Bra000312	20	stress.biotic	stress
VI	Bra013561	20	stress.biotic	stress
VI	Bra013124	20	stress.biotic	stress
VI	Bra013124	20	stress.biotic	stress
VI	Bra014241	20	stress.biotic	stress
VI	Bra029601	21	redox.thioredoxin	redox
VI	Bra002171	21	redox.glutaredoxins	redox
VI	Bra002171	21	redox.glutaredoxins	redox
VI	Bra005786	21	redox.glutaredoxins	redox
VI	Bra004426	21	redox.glutaredoxins	redox
VI	Bra017382	23	nucleotide metabolism.salvage	nucleotide_metabolism
VI	Bra018807	24	Biodegradation of Xenobiotics	Biodegradation_Xenobiotics
VI	Bra004925	24	Biodegradation of Xenobiotics	Biodegradation_Xenobiotics
VI	Bra009808	26	misc.misc2	misc_otherPhosphate
VI	Bra013505	26	misc.cytochrome P450	misc_otherPhosphate
VI	Bra016499	26	misc.cytochrome P450	misc_otherPhosphate
VI	Bra017817	26	misc.cytochrome P450	misc_otherPhosphate

VI	Bra009312	26	misc.cytochrome P450	misc_otherPhosphate
VI	Bra024268	26	misc.peroxidases	misc_otherPhosphate
VI	Bra008057	26	misc.acid and other phosphatases	misc_otherPhosphate
VI	Bra001632	26	misc.myrosinases-lectin-jacalin	misc_otherPhosphate
VI	Bra018406	26	misc.myrosinases-lectin-jacalin	misc_otherPhosphate
VI	Bra001632	26	misc.myrosinases-lectin-jacalin	misc_otherPhosphate
VI	Bra035897	26	misc.invertase/pectin methylesterase inhibitor family protein	misc_otherPhosphate
VI	Bra010076	26	misc.invertase/pectin methylesterase inhibitor family protein	misc_otherPhosphate
VI	Bra013878	26	misc.invertase/pectin methylesterase inhibitor family protein	misc_otherPhosphate
VI	Bra013878	26	misc.invertase/pectin methylesterase inhibitor family protein	misc_otherPhosphate
VI	Bra035191	26	misc.UDP glucosyl and glucoronyl transferases	misc_otherPhosphate
VI	Bra006378	26	misc.UDP glucosyl and glucoronyl transferases	misc_otherPhosphate
VI	Bra006971	26	misc.UDP glucosyl and glucoronyl transferases	misc_otherPhosphate
VI	Bra029010	26	misc.UDP glucosyl and glucoronyl transferases	misc_otherPhosphate
VI	Bra029456	26	misc.protease inhibitor/seed storage/lipid transfer protein (LTP) family protein	misc_otherPhosphate
VI	Bra020845	26	misc.protease inhibitor/seed storage/lipid transfer protein (LTP) family protein	misc_otherPhosphate
VI	Bra013620	26	misc.protease inhibitor/seed storage/lipid transfer protein (LTP) family protein	misc_otherPhosphate
VI	Bra010306	26	misc.GDSL-motif lipase	misc_otherPhosphate
VI	Bra023591	26	misc.oxidases - copper, flavone etc.	misc_otherPhosphate
VI	Bra028255	26	misc.oxidases - copper, flavone etc.	misc_otherPhosphate
VI	Bra036907	27	RNA.processing	RNA
VI	Bra004458	27	RNA.processing	RNA
VI	Bra031522	27	RNA.processing	RNA
VI	Bra025975	27	RNA.transcription	RNA
VI	Bra019952	27	RNA.regulation of transcription	RNA
VI	Bra030308	27	RNA.regulation of transcription	RNA
VI	Bra028966	27	RNA.RNA binding	RNA
VI	Bra022145	27	RNA.RNA binding	RNA
VI	Bra010724	27	RNA.RNA binding	RNA
VI	Bra025568	27	RNA.RNA binding	RNA
VI	Bra013169	28	DNA.synthesis/chromatin structure	DNA
VI	Bra005086	28	DNA.synthesis/chromatin structure	DNA
VI	Bra003916	28	DNA.synthesis/chromatin structure	DNA
VI	Bra003916	28	DNA.synthesis/chromatin structure	DNA
VI	Bra037574	28	DNA.synthesis/chromatin structure	DNA
VI	Bra037574	28	DNA.synthesis/chromatin structure	DNA
VI	Bra040743	28	DNA.synthesis/chromatin structure	DNA
VI	Bra017208	28	DNA.synthesis/chromatin structure	DNA
VI	Bra003760	28	DNA.repair	DNA
VI	Bra018181	28	DNA.unspecified	DNA
VI	Bra040132	29	protein.aa activation	protein
VI	Bra020215	29	protein.aa activation	protein
VI	Bra021278	29	protein.postranslational modification	protein
VI	Bra031918	29	protein.postranslational modification	protein
VI	Bra040944	29	protein.postranslational modification	protein
VI	Bra028832	29	protein.postranslational modification	protein
VI	Bra021794	29	protein.postranslational modification	protein
VI	Bra014673	29	protein.postranslational modification	protein
VI	Bra032510	29	protein.postranslational modification	protein
VI	Bra002254	29	protein.postranslational modification	protein
VI	Bra002254	29	protein.postranslational modification	protein
VI	Bra018397	29	protein.postranslational modification	protein

VI	Bra000077	29	protein.postranslational modification	protein
VI	Bra000077	29	protein.postranslational modification	protein
VI	Bra035360	29	protein.postranslational modification	protein
VI	Bra022388	29	protein.postranslational modification	protein
VI	Bra040230	29	protein.postranslational modification	protein
VI	Bra011330	29	protein.postranslational modification	protein
VI	Bra017346	29	protein.postranslational modification	protein
VI	Bra001518	29	protein.postranslational modification	protein
VI	Bra021900	29	protein.postranslational modification	protein
VI	Bra025051	29	protein.postranslational modification	protein
VI	Bra032051	29	protein.postranslational modification	protein
VI	Bra032051	29	protein.postranslational modification	protein
VI	Bra014243	29	protein.postranslational modification	protein
VI	Bra003201	29	protein.postranslational modification	protein
VI	Bra014215	29	protein.postranslational modification	protein
VI	Bra013340	29	protein.postranslational modification	protein
VI	Bra032188	29	protein.postranslational modification	protein
VI	Bra021687	29	protein.postranslational modification	protein
VI	Bra025596	29	protein.degradation	protein
VI	Bra030665	29	protein.degradation	protein
VI	Bra000254	29	protein.folding	protein
VI	Bra000254	29	protein.folding	protein
VI	Bra031818	29	protein.glycosylation	protein
VI	Bra014244	30	signalling.light	signalling
VI	Bra015896	30	signalling.calcium	signalling
VI	Bra037030	30	signalling.calcium	signalling
VI	Bra032107	30	signalling.calcium	signalling
VI	Bra038811	30	signalling.phosphinositides	signalling
VI	Bra038811	30	signalling.phosphinositides	signalling
VI	Bra037246	30	signalling.phosphinositides	signalling
VI	Bra013450	30	signalling.phosphinositides	signalling
VI	Bra040037	30	signalling.G-proteins	signalling
VI	Bra007771	30	signalling.G-proteins	signalling
VI	Bra017400	30	signalling.G-proteins	signalling
VI	Bra034924	30	signalling.G-proteins	signalling
VI	Bra031555	30	signalling.G-proteins	signalling
VI	Bra008227	30	signalling.G-proteins	signalling
VI	Bra033865	30	signalling.G-proteins	signalling
VI	Bra023880	30	signalling.G-proteins	signalling
VI	Bra020048	30	signalling.G-proteins	signalling
VI	Bra024323	30	signalling.G-proteins	signalling
VI	Bra001232	30	signalling.G-proteins	signalling
VI	Bra027189	30	signalling.G-proteins	signalling
VI	Bra012866	31	cell.organisation	cell
VI	Bra037528	31	cell.organisation	cell
VI	Bra019766	31	cell.organisation	cell
VI	Bra031838	31	cell.organisation	cell
VI	Bra006304	31	cell.organisation	cell
VI	Bra031101	31	cell.organisation	cell
VI	Bra001304	31	cell.organisation	cell
VI	Bra018107	31	cell.division	cell
VI	Bra013232	31	cell.division	cell

VI	Bra026807	31	cell.cycle	cell
VI	Bra004677	31	cell.cycle	cell
VI	Bra036287	31	cell.vesicle transport	cell
VI	Bra032266	31	cell.vesicle transport	cell
VI	Bra016149	31	cell.vesicle transport	cell
VI	Bra019814	31	cell.vesicle transport	cell
VI	Bra021040	33	development.storage proteins	development
VI	Bra010409	33	development.storage proteins	development
VI	Bra038210	33	development.late embryogenesis abundant	development
VI	Bra026289	33	development.unspecified	development
VI	Bra026289	33	development.unspecified	development
VI	Bra023327	33	development.unspecified	development
VI	Bra039878	33	development.unspecified	development
VI	Bra035127	33	development.unspecified	development
VI	Bra031950	33	development.unspecified	development
VI	Bra002921	33	development.unspecified	development
VI	Bra030807	33	development.unspecified	development
VI	Bra001578	33	development.unspecified	development
VI	Bra023207	33	development.unspecified	development
VI	Bra033034	33	development.unspecified	development
VI	Bra039878	33	development.unspecified	development
VI	Bra019197	33	development.unspecified	development
VI	Bra030807	33	development.unspecified	development
VI	Bra002921	33	development.unspecified	development
VI	Bra035723	33	development.unspecified	development
VI	Bra021835	33	development.unspecified	development
VI	Bra003773	33	development.unspecified	development
VI	Bra003387	33	development.unspecified	development
VI	Bra031950	33	development.unspecified	development
VI	Bra005769	34	transport.nucleotides	transport
VI	Bra010298	34	transport.p- and v-ATPases	transport
VI	Bra010299	34	transport.p- and v-ATPases	transport
VI	Bra006695	34	transport.NDP-sugars at the ER	transport
VI	Bra015415	34	transport.metal	transport
VI	Bra010111	34	transport.peptides and oligopeptides	transport
VI	Bra011472	34	transport.unspecified cations	transport
VI	Bra006776	34	transport.ABC transporters and multidrug resistance systems	transport
VI	Bra036575	34	transport.sugars	transport
VI	Bra020794	34	transport.sugars	transport
VI	Bra029645	34	transport.calcium	transport
VI	Bra035661	34	transport.calcium	transport
VI	Bra035661	34	transport.calcium	transport
VI	Bra001676	34	transport.cyclic nucleotide or calcium regulated channels	transport
VI	Bra017005	34	transport.amino acids	transport
VI	Bra021316	34	transport.amino acids	transport
VI	Bra018656	34	transport.nitrate	transport
VI	Bra018656	34	transport.nitrate	transport
VI	Bra017395	34	transport.phosphate	transport
VI	Bra009654	34	transport.metabolite transporters at the mitochondrial membrane	transport
VI	Bra003503	34	transport.metabolite transporters at the mitochondrial membrane	transport
VI	Bra020342	34	transport.metabolite transporters at the mitochondrial membrane	transport
VI	Bra007793	34	transport.metabolite transporters at the mitochondrial membrane	transport

VI	Bra003369	34	transport.misc	transport
VI	Bra014265	34	transport.misc	transport
VI	Bra039365	34	transport.misc	transport
VI	Bra018186	35	not assigned.unknown	not_assigned
VI	Bra035557	35	not assigned.unknown	not_assigned
VI	Bra008857	35	not assigned.unknown	not_assigned
VI	Bra034815	35	not assigned.unknown	not_assigned
VI	Bra011711	35	not assigned.unknown	not_assigned
VI	Bra005792	35	not assigned.unknown	not_assigned
VI	Bra014714	35	not assigned.unknown	not_assigned
VI	Bra000918	35	not assigned.unknown	not_assigned
VI	Bra036156	35	not assigned.unknown	not_assigned
VI	Bra012726	35	not assigned.unknown	not_assigned
VI	Bra008356	35	not assigned.unknown	not_assigned
VI	Bra007271	35	not assigned.unknown	not_assigned
VI	Bra015666	35	not assigned.unknown	not_assigned
VI	Bra002157	35	not assigned.unknown	not_assigned
VI	Bra029078	35	not assigned.unknown	not_assigned
VI	Bra028591	35	not assigned.unknown	not_assigned
VI	Bra030263	35	not assigned.unknown	not_assigned
VI	Bra000602	35	not assigned.unknown	not_assigned
VI	Bra031957	35	not assigned.unknown	not_assigned
VI	Bra010141	35	not assigned.unknown	not_assigned
VI	Bra024090	35	not assigned.unknown	not_assigned
VI	Bra036358	35	not assigned.unknown	not_assigned
VI	Bra020657	35	not assigned.unknown	not_assigned
VI	Bra024873	35	not assigned.unknown	not_assigned
VI	Bra029900	35	not assigned.unknown	not_assigned
VI	Bra019196	35	not assigned.unknown	not_assigned
VI	Bra011264	35	not assigned.unknown	not_assigned
VI	Bra009561	35	not assigned.unknown	not_assigned
VI	Bra034463	35	not assigned.unknown	not_assigned
VI	Bra002711	35	not assigned.unknown	not_assigned
VI	Bra012754	35	not assigned.unknown	not_assigned
VI	Bra037764	35	not assigned.unknown	not_assigned
VI	Bra005433	35	not assigned.unknown	not_assigned
VI	Bra022678	35	not assigned.unknown	not_assigned
VI	Bra020801	35	not assigned.unknown	not_assigned
VI	Bra037823	35	not assigned.unknown	not_assigned
VI	Bra021878	35	not assigned.unknown	not_assigned
VI	Bra018129	35	not assigned.unknown	not_assigned
VI	Bra010240	35	not assigned.unknown	not_assigned
VI	Bra036892	35	not assigned.unknown	not_assigned
VI	Bra035380	35	not assigned.unknown	not_assigned
VI	Bra023335	35	not assigned.unknown	not_assigned
VI	Bra018368	35	not assigned.unknown	not_assigned
VI	Bra033421	35	not assigned.unknown	not_assigned
VI	Bra034336	35	not assigned.unknown	not_assigned
VI	Bra016884	35	not assigned.unknown	not_assigned
VI	Bra024872	35	not assigned.unknown	not_assigned
VI	Bra025990	35	not assigned.unknown	not_assigned
VI	Bra027383	35	not assigned.unknown	not_assigned

VI	Bra010506	35	not assigned.unknown	not_assigned
VI	Bra035582	35	not assigned.unknown	not_assigned
VI	Bra027721	35	not assigned.unknown	not_assigned
VI	Bra034997	35	not assigned.unknown	not_assigned
VI	Bra017459	35	not assigned.unknown	not_assigned
VI	Bra020645	35	not assigned.unknown	not_assigned
VI	Bra040780	35	not assigned.unknown	not_assigned
VI	Bra025898	35	not assigned.unknown	not_assigned
VI	Bra030410	35	not assigned.unknown	not_assigned
VI	Bra031278	35	not assigned.unknown	not_assigned
VI	Bra021526	35	not assigned.unknown	not_assigned
VI	Bra007440	35	not assigned.unknown	not_assigned
VI	Bra023308	35	not assigned.unknown	not_assigned
VI	Bra015816	35	not assigned.unknown	not_assigned
VI	Bra025637	35	not assigned.unknown	not_assigned
VI	Bra001558	35	not assigned.unknown	not_assigned
VI	Bra016337	35	not assigned.unknown	not_assigned
VI	Bra019460	35	not assigned.unknown	not_assigned
VI	Bra010619	35	not assigned.unknown	not_assigned
VI	Bra020594	35	not assigned.unknown	not_assigned
VI	Bra026394	35	not assigned.unknown	not_assigned
VI	Bra018961	35	not assigned.unknown	not_assigned
VI	Bra029043	35	not assigned.unknown	not_assigned
VI	Bra030761	35	not assigned.unknown	not_assigned
VI	Bra032342	35	not assigned.unknown	not_assigned
VI	Bra027133	35	not assigned.unknown	not_assigned
VI	Bra022519	35	not assigned.unknown	not_assigned
VI	Bra039565	35	not assigned.unknown	not_assigned
VI	Bra010619	35	not assigned.unknown	not_assigned
VI	Bra020626	35	not assigned.unknown	not_assigned
VI	Bra036737	35	not assigned.unknown	not_assigned
VI	Bra016528	35	not assigned.unknown	not_assigned
VI	Bra023119	35	not assigned.unknown	not_assigned
VI	Bra006405	35	not assigned.unknown	not_assigned
VI	Bra007672	35	not assigned.unknown	not_assigned
VI	Bra020031	35	not assigned.unknown	not_assigned
VI	Bra002972	35	not assigned.unknown	not_assigned
VI	Bra018151	35	not assigned.unknown	not_assigned
VI	Bra008634	35	not assigned.unknown	not_assigned
VI	Bra031458	35	not assigned.unknown	not_assigned
VI	Bra038311	35	not assigned.unknown	not_assigned
VI	Bra039640	35	not assigned.unknown	not_assigned
VI	Bra013590	35	not assigned.unknown	not_assigned
VI	Bra015011	35	not assigned.unknown	not_assigned
VI	Bra009568	35	not assigned.unknown	not_assigned
VI	Bra008394	35	not assigned.unknown	not_assigned
VI	Bra008054	35	not assigned.unknown	not_assigned
VI	Bra021092	35	not assigned.unknown	not_assigned
VI	Bra028732	35	not assigned.unknown	not_assigned
VI	Bra001690	35	not assigned.unknown	not_assigned
VI	Bra019104	35	not assigned.unknown	not_assigned
VI	Bra005604	35	not assigned.unknown	not_assigned

VI	Bra028782	35	not assigned.unknown	not_assigned
VI	Bra024189	35	not assigned.unknown	not_assigned
VI	Bra034481	35	not assigned.unknown	not_assigned
VI	Bra011336	35	not assigned.unknown	not_assigned
VI	Bra009344	35	not assigned.unknown	not_assigned
VI	Bra014996	35	not assigned.unknown	not_assigned
VI	Bra032294	35	not assigned.unknown	not_assigned
VI	Bra017050	35	not assigned.unknown	not_assigned
VI	Bra004145	35	not assigned.unknown	not_assigned
VI	Bra020932	35	not assigned.unknown	not_assigned
VI	Bra030262	35	not assigned.unknown	not_assigned
VI	Bra001500	35	not assigned.unknown	not_assigned
VI	Bra030809	35	not assigned.unknown	not_assigned
VI	Bra023407	35	not assigned.unknown	not_assigned
VI	Bra026915	35	not assigned.unknown	not_assigned
VI	Bra029676	35	not assigned.unknown	not_assigned
VI	Bra033829	35	not assigned.unknown	not_assigned
VI	Bra002363	35	not assigned.unknown	not_assigned
VI	Bra005731	35	not assigned.unknown	not_assigned
VI	Bra032053	35	not assigned.unknown	not_assigned
VI	Bra014704	35	not assigned.unknown	not_assigned
VI	Bra023142	35	not assigned.unknown	not_assigned
VI	Bra038190	35	not assigned.unknown	not_assigned
VI	Bra038241	35	not assigned.unknown	not_assigned
VI	Bra004664	35	not assigned.unknown	not_assigned
VI	Bra013927	35	not assigned.unknown	not_assigned
VI	Bra034046	35	not assigned.unknown	not_assigned
VI	Bra008943	35	not assigned.unknown	not_assigned
VI	Bra015888	35	not assigned.unknown	not_assigned
VI	Bra023194	35	not assigned.unknown	not_assigned
VI	Bra013177	35	not assigned.unknown	not_assigned
VI	Bra032414	35	not assigned.unknown	not_assigned
VI	Bra013583	35	not assigned.unknown	not_assigned
VI	Bra006255	35	not assigned.unknown	not_assigned
VI	Bra029260	35	not assigned.unknown	not_assigned
VI	Bra019873	35	not assigned.unknown	not_assigned
VI	Bra039346	35	not assigned.unknown	not_assigned
VI	Bra040918	35	not assigned.unknown	not_assigned
VI	Bra022311	35	not assigned.unknown	not_assigned
VI	Bra002702	35	not assigned.unknown	not_assigned
VI	Bra022469	35	not assigned.unknown	not_assigned
VI	Bra032201	35	not assigned.unknown	not_assigned
VI	Bra000597	35	not assigned.unknown	not_assigned
VI	Bra016391	35	not assigned.unknown	not_assigned
VI	Bra013067	35	not assigned.unknown	not_assigned
VI	Bra022589	35	not assigned.unknown	not_assigned
VI	Bra018427	35	not assigned.unknown	not_assigned
VI	Bra021526	35	not assigned.unknown	not_assigned
VI	Bra038434	35	not assigned.unknown	not_assigned
VI	Bra032816	35	not assigned.unknown	not_assigned
VI	Bra029312	35	not assigned.unknown	not_assigned
VI	Bra010344	35	not assigned.unknown	not_assigned

VI	Bra008090	35	not assigned.unknown	not_assigned
VI	Bra023200	35	not assigned.unknown	not_assigned
VI	Bra008281	35	not assigned.unknown	not_assigned
VI	Bra014224	35	not assigned.unknown	not_assigned
VI	Bra000515	35	not assigned.unknown	not_assigned
VI	Bra022527	35	not assigned.unknown	not_assigned
VI	Bra007938	35	not assigned.unknown	not_assigned
VI	Bra030932	35	not assigned.unknown	not_assigned
VI	Bra040798	35	not assigned.unknown	not_assigned
VI	Bra027233	35	not assigned.unknown	not_assigned
VI	Bra020244	35	not assigned.unknown	not_assigned
VI	Bra038193	35	not assigned.unknown	not_assigned
VI	Bra041019	35	not assigned.unknown	not_assigned
VI	Bra008265	1	PS.lightreaction.photosystem II.LHC-II	Photosynthesis
VI	Bra004504	1	PS.lightreaction.photosystem I.PSI polypeptide subunits	Photosynthesis
VI	Bra021688	1	PS.lightreaction.ATP synthase	Photosynthesis
VI	Bra001923	10	cell wall.precursor synthesis.GAE	cell_wall
VI	Bra039163	10	cell wall.cellulose synthesis.COBRA	cell_wall
VI	Bra003432	10	cell wall.cell wall proteins.AGPs.AGP	cell_wall
VI	Bra012198	10	cell wall.degradation.cellulases and beta -1,4-glucanases	cell_wall
VI	Bra036281	10	cell wall.degradation.cellulases and beta -1,4-glucanases	cell_wall
VI	Bra036392	10	cell wall.degradation.mannan-xylose-arabinose-fucose	cell_wall
VI	Bra009398	10	cell wall.degradation.mannan-xylose-arabinose-fucose	cell_wall
VI	Bra017412	10	cell wall.degradation.pectate lyases and polygalacturonases	cell_wall
VI	Bra004789	10	cell wall.degradation.pectate lyases and polygalacturonases	cell_wall
VI	Bra004789	10	cell wall.degradation.pectate lyases and polygalacturonases	cell_wall
VI	Bra014620	10	cell wall.degradation.pectate lyases and polygalacturonases	cell_wall
VI	Bra040790	10	cell wall.degradation.pectate lyases and polygalacturonases	cell_wall
VI	Bra007626	10	cell wall.degradation.pectate lyases and polygalacturonases	cell_wall
VI	Bra001546	10	cell wall.pectin*esterases.PME	cell_wall
VI	Bra036279	10	cell wall.pectin*esterases.PME	cell_wall
VI	Bra001307	10	cell wall.pectin*esterases.acetyl esterase	cell_wall
VI	Bra013230	11	lipid metabolism.FA synthesis and FA elongation.ACP thioesterase	lipid_metabolism
VI	Bra005961	11	lipid metabolism.Phospholipid synthesis.diacylglycerol kinase	lipid_metabolism
VI	Bra040272	11	lipid metabolism."exotics" (steroids, squalene etc).sphingolipids	lipid_metabolism
VI	Bra006785	11	lipid metabolism.lipid degradation.lysophospholipases.glycerophosphodiester phosphodiesterase	lipid_metabolism
VI	Bra015626	13	amino acid metabolism.synthesis.aspartate family.methionine.homocysteine S-methyltransferase	amino_acid_metabolism
VI	Bra000338	13	amino acid metabolism.synthesis.aspartate family.lysine.dihydrodipicolinate reductase	amino_acid_metabolism
VI	Bra007628	13	amino acid metabolism.synthesis.aromatic aa.tryptophan	amino_acid_metabolism
VI	Bra020640	13	amino acid metabolism.synthesis.aromatic aa.tryptophan	amino_acid_metabolism
VI	Bra028736	13	amino acid metabolism.degradation.aspartate family.lysine	amino_acid_metabolism
VI	Bra020813	13	amino acid metabolism.degradation.aromatic aa.tyrosine	amino_acid_metabolism
VI	Bra011522	16	secondary metabolism.isoprenoids.non-mevalonate pathway.HDR	secondary_metabolism
VI	Bra011522	16	secondary metabolism.isoprenoids.non-mevalonate pathway.HDR	secondary_metabolism
VI	Bra006354	16	secondary metabolism.isoprenoids.mevalonate pathway.isopentenyl pyrophosphate:dimethyllallyl pyrophosphate isomerase	secondary_metabolism
VI	Bra006354	16	secondary metabolism.isoprenoids.mevalonate pathway.isopentenyl pyrophosphate:dimethyllallyl pyrophosphate isomerase	secondary_metabolism
VI	Bra002476	16	secondary metabolism.isoprenoids.terpenoids	secondary_metabolism
VI	Bra012232	16	secondary metabolism.phenylpropanoids.lignin biosynthesis.4CL	secondary_metabolism
VI	Bra015944	16	secondary metabolism.N misc.alkaloid-like	secondary_metabolism

VI	Bra021635	17	hormone metabolism.abscisic acid.signal transduction	hormone_metabolism
VI	Bra007720	17	hormone metabolism.auxin.signal transduction	hormone_metabolism
VI	Bra008615	17	hormone metabolism.auxin.signal transduction	hormone_metabolism
VI	Bra008105	17	hormone metabolism.auxin.signal transduction	hormone_metabolism
VI	Bra036830	17	hormone metabolism.auxin.induced-regulated-responsive-activated	hormone_metabolism
VI	Bra024882	17	hormone metabolism.auxin.induced-regulated-responsive-activated	hormone_metabolism
VI	Bra008209	17	hormone metabolism.auxin.induced-regulated-responsive-activated	hormone_metabolism
VI	Bra032097	17	hormone metabolism.auxin.induced-regulated-responsive-activated	hormone_metabolism
VI	Bra032097	17	hormone metabolism.auxin.induced-regulated-responsive-activated	hormone_metabolism
VI	Bra001038	17	hormone metabolism.auxin.induced-regulated-responsive-activated	hormone_metabolism
VI	Bra023406	17	hormone metabolism.auxin.induced-regulated-responsive-activated	hormone_metabolism
VI	Bra006319	17	hormone metabolism.auxin.induced-regulated-responsive-activated	hormone_metabolism
VI	Bra005293	17	hormone metabolism.auxin.induced-regulated-responsive-activated	hormone_metabolism
VI	Bra032779	17	hormone metabolism.brassinosteroid.synthesis-degradation.sterols.other	hormone_metabolism
VI	Bra034366	17	hormone metabolism.cytokinin.synthesis-degradation	hormone_metabolism
VI	Bra022920	17	hormone metabolism.ethylene.synthesis-degradation	hormone_metabolism
VI	Bra026856	17	hormone metabolism.ethylene.synthesis-degradation	hormone_metabolism
VI	Bra026456	17	hormone metabolism.ethylene.synthesis-degradation.1-aminocyclopropane-1-carboxylate synthase	hormone_metabolism
VI	Bra036057	17	hormone metabolism.ethylene.signal transduction	hormone_metabolism
VI	Bra037272	20	stress.biotic.signalling.MLO-like	stress
VI	Bra031161	20	stress.biotic.PR-proteins	stress
VI	Bra006556	20	stress.biotic.PR-proteins	stress
VI	Bra027599	20	stress.biotic.PR-proteins	stress
VI	Bra025494	20	stress.biotic.PR-proteins	stress
VI	Bra025494	20	stress.biotic.PR-proteins	stress
VI	Bra029034	20	stress.biotic.PR-proteins.plant defensins	stress
VI	Bra040525	20	stress.biotic.PR-proteins.plant defensins	stress
VI	Bra008084	20	stress.biotic.PR-proteins.proteinase inhibitors.trypsin inhibitor	stress
VI	Bra016072	20	stress.biotic.PR-proteins.proteinase inhibitors.trypsin inhibitor	stress
VI	Bra025942	20	stress.biotic.PR-proteins.proteinase inhibitors.trypsin inhibitor	stress
VI	Bra010049	20	stress.abiotic.heat	stress
VI	Bra018571	20	stress.abiotic.heat	stress
VI	Bra004301	20	stress.abiotic.heat	stress
VI	Bra001071	20	stress.abiotic.heat	stress
VI	Bra032044	20	stress.abiotic.heat	stress
VI	Bra033754	20	stress.abiotic.drought/salt	stress
VI	Bra005438	20	stress.abiotic.drought/salt	stress
VI	Bra033754	20	stress.abiotic.drought/salt	stress
VI	Bra039550	20	stress.abiotic.drought/salt	stress
VI	Bra039550	20	stress.abiotic.drought/salt	stress
VI	Bra034441	20	stress.abiotic.drought/salt	stress
VI	Bra034157	20	stress.abiotic.touch/wounding	stress
VI	Bra034157	20	stress.abiotic.touch/wounding	stress
VI	Bra003784	20	stress.abiotic.unspecified	stress
VI	Bra027979	20	stress.abiotic.unspecified	stress
VI	Bra040243	20	stress.abiotic.unspecified	stress
VI	Bra012635	20	stress.abiotic.unspecified	stress
VI	Bra012635	20	stress.abiotic.unspecified	stress
VI	Bra028424	20	stress.abiotic.unspecified	stress
VI	Bra028424	20	stress.abiotic.unspecified	stress
VI	Bra027979	20	stress.abiotic.unspecified	stress

VI	Bra000302	21	redox.ascorbate and glutathione.glutathione	redox
VI	Bra000302	21	redox.ascorbate and glutathione.glutathione	redox
VI	Bra008662	22	polyamine metabolism.synthesis.SAM decarboxylase	polyamine_metabolism
VI	Bra003204	23	nucleotide metabolism.synthesis.purine.AIR synthase	nucleotide_metabolism
VI	Bra016120	23	nucleotide metabolism.salvage.phosphoribosyltransferases.hgprt	nucleotide_metabolism
VI	Bra002295	26	misc.beta 1,3 glucan hydrolases.glucan endo-1,3-beta-glucosidase	misc_otherPhosphate
VI	Bra001918	26	misc.beta 1,3 glucan hydrolases.glucan endo-1,3-beta-glucosidase	misc_otherPhosphate
VI	Bra011910	27	RNA.processing.splicing	RNA
VI	Bra018446	27	RNA.processing.splicing	RNA
VI	Bra018446	27	RNA.processing.splicing	RNA
VI	Bra040940	27	RNA.processing.splicing	RNA
VI	Bra013431	27	RNA.processing.splicing	RNA
VI	Bra029177	27	RNA.processing.splicing	RNA
VI	Bra029275	27	RNA.processing.RNA helicase	RNA
VI	Bra023227	27	RNA.processing.RNA helicase	RNA
VI	Bra010776	27	RNA.regulation of transcription.ARF, Auxin Response Factor family	RNA
VI	Bra023153	27	RNA.regulation of transcription.C2H2 zinc finger family	RNA
VI	Bra038996	27	RNA.regulation of transcription.C2H2 zinc finger family	RNA
VI	Bra038996	27	RNA.regulation of transcription.C2H2 zinc finger family	RNA
VI	Bra018535	27	RNA.regulation of transcription.C2H2 zinc finger family	RNA
VI	Bra012034	27	RNA.regulation of transcription.C2H2 zinc finger family	RNA
VI	Bra010334	27	RNA.regulation of transcription.C3H zinc finger family	RNA
VI	Bra027900	27	RNA.regulation of transcription.C3H zinc finger family	RNA
VI	Bra005130	27	RNA.regulation of transcription.C3H zinc finger family	RNA
VI	Bra010334	27	RNA.regulation of transcription.C3H zinc finger family	RNA
VI	Bra032395	27	RNA.regulation of transcription.CCAAT box binding factor family, HAP2	RNA
VI	Bra005124	27	RNA.regulation of transcription.G2-like transcription factor family, GARP	RNA
VI	Bra005124	27	RNA.regulation of transcription.G2-like transcription factor family, GARP	RNA
VI	Bra036679	27	RNA.regulation of transcription.G2-like transcription factor family, GARP	RNA
VI	Bra002108	27	RNA.regulation of transcription.HB,Homeobox transcription factor family	RNA
VI	Bra033492	27	RNA.regulation of transcription.MADS box transcription factor family	RNA
VI	Bra033492	27	RNA.regulation of transcription.MADS box transcription factor family	RNA
VI	Bra020242	27	RNA.regulation of transcription.MADS box transcription factor family	RNA
VI	Bra025529	27	RNA.regulation of transcription.MYB domain transcription factor family	RNA
VI	Bra005482	27	RNA.regulation of transcription.MYB domain transcription factor family	RNA
VI	Bra035604	27	RNA.regulation of transcription.MYB domain transcription factor family	RNA
VI	Bra038968	27	RNA.regulation of transcription.MYB domain transcription factor family	RNA
VI	Bra007975	27	RNA.regulation of transcription.AP2/EREBP, APETALA2/Ethylene-responsive element binding protein family	RNA
VI	Bra027614	27	RNA.regulation of transcription.AP2/EREBP, APETALA2/Ethylene-responsive element binding protein family	RNA
VI	Bra027614	27	RNA.regulation of transcription.AP2/EREBP, APETALA2/Ethylene-responsive element binding protein family	RNA
VI	Bra010383	27	RNA.regulation of transcription.AP2/EREBP, APETALA2/Ethylene-responsive element binding protein family	RNA
VI	Bra012123	27	RNA.regulation of transcription.AP2/EREBP, APETALA2/Ethylene-responsive element binding protein family	RNA
VI	Bra028824	27	RNA.regulation of transcription.Trihelix, Triple-Helix transcription factor family	RNA
VI	Bra015716	27	RNA.regulation of transcription.Trihelix, Triple-Helix transcription factor family	RNA
VI	Bra015716	27	RNA.regulation of transcription.Trihelix, Triple-Helix transcription factor family	RNA
VI	Bra023112	27	RNA.regulation of transcription.WRKY domain transcription factor family	RNA
VI	Bra023112	27	RNA.regulation of transcription.WRKY domain transcription factor family	RNA
VI	Bra030314	27	RNA.regulation of transcription.bZIP transcription factor family	RNA

VI	Bra008192	27	RNA.regulation of transcription.bZIP transcription factor family	RNA
VI	Bra023172	27	RNA.regulation of transcription.Argonaute	RNA
VI	Bra005508	27	RNA.regulation of transcription.Aux/IAA family	RNA
VI	Bra004831	27	RNA.regulation of transcription.Bromodomain proteins	RNA
VI	Bra024250	27	RNA.regulation of transcription.Chromatin Remodeling Factors	RNA
VI	Bra004441	27	RNA.regulation of transcription.Chromatin Remodeling Factors	RNA
VI	Bra007814	27	RNA.regulation of transcription.Chromatin Remodeling Factors	RNA
VI	Bra038573	27	RNA.regulation of transcription.Chromatin Remodeling Factors	RNA
VI	Bra038573	27	RNA.regulation of transcription.Chromatin Remodeling Factors	RNA
VI	Bra031402	27	RNA.regulation of transcription.GeBP like	RNA
VI	Bra001643	27	RNA.regulation of transcription.ARR	RNA
VI	Bra009399	27	RNA.regulation of transcription.Histone acetyltransferases	RNA
VI	Bra009399	27	RNA.regulation of transcription.Histone acetyltransferases	RNA
VI	Bra027644	27	RNA.regulation of transcription.JUMONJI family	RNA
VI	Bra020318	27	RNA.regulation of transcription.Methyl binding domain proteins	RNA
VI	Bra000560	27	RNA.regulation of transcription.bHLH,Basic Helix-Loop-Helix family	RNA
VI	Bra017520	27	RNA.regulation of transcription.bHLH,Basic Helix-Loop-Helix family	RNA
VI	Bra021219	27	RNA.regulation of transcription.bHLH,Basic Helix-Loop-Helix family	RNA
VI	Bra025226	27	RNA.regulation of transcription.bHLH,Basic Helix-Loop-Helix family	RNA
VI	Bra000979	27	RNA.regulation of transcription.bHLH,Basic Helix-Loop-Helix family	RNA
VI	Bra037478	27	RNA.regulation of transcription.bHLH,Basic Helix-Loop-Helix family	RNA
VI	Bra003260	27	RNA.regulation of transcription.bHLH,Basic Helix-Loop-Helix family	RNA
VI	Bra013238	27	RNA.regulation of transcription.putative transcription regulator	RNA
VI	Bra028349	27	RNA.regulation of transcription.putative transcription regulator	RNA
VI	Bra000806	27	RNA.regulation of transcription.putative transcription regulator	RNA
VI	Bra035940	27	RNA.regulation of transcription.putative transcription regulator	RNA
VI	Bra020249	27	RNA.regulation of transcription.putative transcription regulator	RNA
VI	Bra029002	27	RNA.regulation of transcription.putative transcription regulator	RNA
VI	Bra009470	27	RNA.regulation of transcription.putative transcription regulator	RNA
VI	Bra000593	27	RNA.regulation of transcription.putative transcription regulator	RNA
VI	Bra021721	27	RNA.regulation of transcription.SET-domain transcriptional regulator family	RNA
VI	Bra002516	27	RNA.regulation of transcription.C2C2(Zn) CO-like, Constans-like zinc finger family	RNA
VI	Bra029309	27	RNA.regulation of transcription.Zn-finger(CCHC)	RNA
VI	Bra028701	27	RNA.regulation of transcription.Zn-finger(CCHC)	RNA
VI	Bra029309	27	RNA.regulation of transcription.Zn-finger(CCHC)	RNA
VI	Bra040603	27	RNA.regulation of transcription.Zn-finger(CCHC)	RNA
VI	Bra010136	27	RNA.regulation of transcription.C2C2(Zn) DOF zinc finger family	RNA
VI	Bra010548	27	RNA.regulation of transcription.C2C2(Zn) DOF zinc finger family	RNA
VI	Bra037401	27	RNA.regulation of transcription.C2C2(Zn) DOF zinc finger family	RNA
VI	Bra011552	27	RNA.regulation of transcription.C2C2(Zn) GATA transcription factor family	RNA
VI	Bra040288	27	RNA.regulation of transcription.C2C2(Zn) GATA transcription factor family	RNA
VI	Bra040288	27	RNA.regulation of transcription.C2C2(Zn) GATA transcription factor family	RNA
VI	Bra019227	27	RNA.regulation of transcription.C2C2(Zn) GATA transcription factor family	RNA
VI	Bra019227	27	RNA.regulation of transcription.C2C2(Zn) GATA transcription factor family	RNA
VI	Bra038486	27	RNA.regulation of transcription.unclassified	RNA
VI	Bra017832	27	RNA.regulation of transcription.unclassified	RNA
VI	Bra036689	27	RNA.regulation of transcription.unclassified	RNA
VI	Bra004826	27	RNA.regulation of transcription.unclassified	RNA
VI	Bra038619	27	RNA.regulation of transcription.unclassified	RNA
VI	Bra000005	27	RNA.regulation of transcription.unclassified	RNA
VI	Bra023016	27	RNA.regulation of transcription.unclassified	RNA
VI	Bra023016	27	RNA.regulation of transcription.unclassified	RNA

VI	Bra031691	27	RNA.regulation of transcription.unclassified	RNA
VI	Bra036286	27	RNA.regulation of transcription.unclassified	RNA
VI	Bra001059	29	protein.aa activation.tyrosine-tRNA ligase	protein
VI	Bra024018	29	protein.aa activation.aspartate-tRNA ligase	protein
VI	Bra008658	29	protein.aa activation.phenylalanine-tRNA ligase	protein
VI	Bra029197	29	protein.aa activation.bifunctional aminoacyl-tRNA synthetase	protein
VI	Bra033351	29	protein.synthesis.ribosomal protein.eukaryotic.60S subunit.L22	protein
VI	Bra039698	29	protein.synthesis.initiation	protein
VI	Bra006126	29	protein.synthesis.initiation	protein
VI	Bra025203	29	protein.synthesis.initiation	protein
VI	Bra007832	29	protein.synthesis.initiation	protein
VI	Bra025203	29	protein.synthesis.initiation	protein
VI	Bra032059	29	protein.synthesis.initiation	protein
VI	Bra000034	29	protein.synthesis.ribosomal RNA	protein
VI	Bra004005	29	protein.targeting.nucleus	protein
VI	Bra030666	29	protein.targeting.chloroplast	protein
VI	Bra018582	29	protein.targeting.secretory pathway.vacuole	protein
VI	Bra007203	29	protein.targeting.unknown	protein
VI	Bra002541	29	protein.postranslational modification.kinase.receptor like cytoplasmatic kinase VII	protein
VI	Bra006890	29	protein.postranslational modification.kinase.receptor like cytoplasmatic kinase VII	protein
VI	Bra016342	29	protein.postranslational modification.kinase.receptor like cytoplasmatic kinase VII	protein
VI	Bra014711	29	protein.postranslational modification.kinase.receptor like cytoplasmatic kinase VII	protein
VI	Bra008387	29	protein.postranslational modification.kinase.receptor like cytoplasmatic kinase VII	protein
VI	Bra033176	29	protein.postranslational modification.kinase.receptor like cytoplasmatic kinase VII	protein
VI	Bra018971	29	protein.postranslational modification.kinase.receptor like cytoplasmatic kinase VII	protein
VI	Bra018971	29	protein.postranslational modification.kinase.receptor like cytoplasmatic kinase VII	protein
VI	Bra015053	29	protein.postranslational modification.kinase.receptor like cytoplasmatic kinase VII	protein
VI	Bra034709	29	protein.postranslational modification.kinase.receptor like cytoplasmatic kinase VII	protein
VI	Bra002974	29	protein.postranslational modification.kinase.receptor like cytoplasmatic kinase VII	protein
VI	Bra033170	29	protein.degradation.subtilases	protein
VI	Bra006153	29	protein.degradation.aspartate protease	protein
VI	Bra020219	29	protein.degradation.aspartate protease	protein
VI	Bra006153	29	protein.degradation.aspartate protease	protein
VI	Bra016629	29	protein.degradation.ubiquitin	protein
VI	Bra016618	29	protein.degradation.ubiquitin.ubiquitin protease	protein
VI	Bra028566	29	protein.degradation.ubiquitin.ubiquitin protease	protein
VI	Bra004696	29	protein.degradation.ubiquitin.ubiquitin protease	protein
VI	Bra003330	29	protein.degradation.ubiquitin.ubiquitin protease	protein
VI	Bra000258	29	protein.degradation.ubiquitin.ubiquitin protease	protein
VI	Bra005348	29	protein.degradation.ubiquitin.ubiquitin	protein
VI	Bra005348	29	protein.degradation.ubiquitin.ubiquitin	protein
VI	Bra006038	29	protein.degradation.ubiquitin.proteasom	protein
VI	Bra028755	29	protein.degradation.ubiquitin.E3.APC	protein
VI	Bra021160	29	protein.degradation.ubiquitin.E3.RING	protein
VI	Bra004373	29	protein.degradation.ubiquitin.E3.RING	protein
VI	Bra019242	29	protein.degradation.ubiquitin.E3.RING	protein
VI	Bra013267	29	protein.degradation.ubiquitin.E3.RING	protein
VI	Bra022754	29	protein.degradation.ubiquitin.E3.RING	protein
VI	Bra014684	29	protein.degradation.ubiquitin.E3.RING	protein
VI	Bra003830	29	protein.degradation.ubiquitin.E3.RING	protein
VI	Bra007810	29	protein.degradation.ubiquitin.E3.RING	protein
VI	Bra029250	29	protein.degradation.ubiquitin.E3.RING	protein

VI	Bra033244	29	protein.degradation.ubiquitin.E3.RING	protein
VI	Bra017569	29	protein.degradation.ubiquitin.E3.SCF.cullin	protein
VI	Bra001142	29	protein.degradation.ubiquitin.E3.SCF.FBOX	protein
VI	Bra028158	29	protein.degradation.ubiquitin.E3.SCF.FBOX	protein
VI	Bra010649	29	protein.degradation.ubiquitin.E3.SCF.FBOX	protein
VI	Bra012527	29	protein.degradation.ubiquitin.E3.SCF.FBOX	protein
VI	Bra028686	29	protein.degradation.ubiquitin.E3.SCF.FBOX	protein
VI	Bra025562	29	protein.degradation.ubiquitin.E3.SCF.FBOX	protein
VI	Bra013995	29	protein.degradation.ubiquitin.E3.SCF.FBOX	protein
VI	Bra030837	29	protein.degradation.ubiquitin.E3.SCF.FBOX	protein
VI	Bra031360	29	protein.degradation.ubiquitin.E3.SCF.FBOX	protein
VI	Bra017245	29	protein.degradation.ubiquitin.E3.SCF.FBOX	protein
VI	Bra038831	29	protein.degradation.ubiquitin.E3.SCF.FBOX	protein
VI	Bra025046	29	protein.degradation.cysteine protease	protein
VI	Bra009333	29	protein.degradation.serine protease	protein
VI	Bra003063	29	protein.degradation.serine protease	protein
VI	Bra014520	29	protein.degradation.metalloprotease	protein
VI	Bra014520	29	protein.degradation.metalloprotease	protein
VI	Bra001738	29	protein.degradation.metalloprotease	protein
VI	Bra019140	29	protein.degradation.AAA type	protein
VI	Bra003561	29	protein.degradation.AAA type	protein
VI	Bra007040	30	signalling.in sugar and nutrient physiology	signalling
VI	Bra005591	30	signalling.in sugar and nutrient physiology	signalling
VI	Bra036873	30	signalling.receptor kinases.leucine rich repeat XI	signalling
VI	Bra001399	30	signalling.receptor kinases.leucine rich repeat XI	signalling
VI	Bra033984	30	signalling.receptor kinases.DUF 26	signalling
VI	Bra000746	30	signalling.receptor kinases.DUF 26	signalling
VI	Bra040315	30	signalling.receptor kinases.DUF 26	signalling
VI	Bra015746	30	signalling.receptor kinases.DUF 26	signalling
VI	Bra002546	30	signalling.receptor kinases.misc	signalling
VI	Bra030416	30	signalling.receptor kinases.misc	signalling
VI	Bra031318	30	signalling.receptor kinases.misc	signalling
VI	Bra006368	30	signalling.phosphinositides.inositol-1,3,4-trisphosphate 5/6-kinase	signalling
VI	Bra006368	30	signalling.phosphinositides.inositol-1,3,4-trisphosphate 5/6-kinase	signalling
VI	Bra009723	31	cell.division.plastid	cell
VI	Bra032136	31	cell.cycle.peptidylprolyl isomerase	cell
VI	Bra014849	31	cell.cycle.peptidylprolyl isomerase	cell
VI	Bra036411	4	glycolysis.cytosolic branch.pyruvate kinase (PK)	glycolysis
VI	Bra036411	4	glycolysis.cytosolic branch.pyruvate kinase (PK)	glycolysis
VI	Bra010637	4	glycolysis.unclear/dually targeted.phosphofructokinase (PFK)	glycolysis
VI	Bra035715	8	TCA / org. transformation.TCA.pyruvate DH.E1	TCA
VI	Bra006107	8	TCA / org. transformation.other organic acid transformaitons.malic	TCA
VI	Bra006107	8	TCA / org. transformation.other organic acid transformaitons.malic	TCA
VI	Bra037347	8	TCA / org. transformation.other organic acid transformaitons.malic	TCA
VII	Bra018112	3	minor CHO metabolism.others	minor_CHOmetabolism
VII	Bra007417	6	gluconeogenese/ glyoxylate cycle.citrate synthase	glyoxylate_cycle
VII	Bra031351	9	mitochondrial electron transport / ATP synthesis.alternative oxidase	ATP_synthesis
VII	Bra012969	9	mitochondrial electron transport / ATP synthesis.cytochrome c oxidase	ATP_synthesis
VII	Bra036225	10	cell wall.modification	cell_wall
VII	Bra026630	10	cell wall.modification	cell_wall
VII	Bra024089	10	cell wall.modification	cell_wall
VII	Bra036303	11	lipid metabolism.Phospholipid synthesis	lipid_metabolism

VII	Bra036788	11	lipid metabolism.lipid transfer proteins etc	lipid_metabolism
VII	Bra015782	11	lipid metabolism."exotics" (steroids, squalene etc)	lipid_metabolism
VII	Bra002982	11	lipid metabolism."exotics" (steroids, squalene etc)	lipid_metabolism
VII	Bra016627	13	amino acid metabolism	amino_acid_metabolism
VII	Bra009773	15	metal handling.binding, chelation and storage	metal_handling
VII	Bra009773	15	metal handling.binding, chelation and storage	metal_handling
VII	Bra001309	15	metal handling.binding, chelation and storage	metal_handling
VII	Bra009595	15	metal handling.binding, chelation and storage	metal_handling
VII	Bra003234	15	metal handling.binding, chelation and storage	metal_handling
VII	Bra003234	15	metal handling.binding, chelation and storage	metal_handling
VII	Bra027254	15	metal handling.binding, chelation and storage	metal_handling
VII	Bra028875	15	metal handling.binding, chelation and storage	metal_handling
VII	Bra033733	16	secondary metabolism.phenylpropanoids	secondary_metabolism
VII	Bra015335	18	Co-factor and vitamine metabolism	Co-factor_vitamine_metabolism
VII	Bra015335	18	Co-factor and vitamine metabolism	Co-factor_vitamine_metabolism
VII	Bra010732	20	stress.biotic	stress
VII	Bra018810	20	stress.biotic	stress
VII	Bra018810	20	stress.biotic	stress
VII	Bra037412	20	stress.biotic	stress
VII	Bra003740	20	stress.biotic	stress
VII	Bra015809	20	stress.biotic	stress
VII	Bra028445	21	redox.thioredoxin	redox
VII	Bra027469	21	redox.thioredoxin	redox
VII	Bra031940	23	nucleotide metabolism.degradation	nucleotide_metabolism
VII	Bra011821	26	misc.cytochrome P450	misc_otherPhosphate
VII	Bra010332	26	misc.acid and other phosphatases	misc_otherPhosphate
VII	Bra020949	26	misc.myrosinases-lectin-jacalin	misc_otherPhosphate
VII	Bra012366	26	misc.invertase/pectin methylesterase inhibitor family protein	misc_otherPhosphate
VII	Bra007821	26	misc.plastocyanin-like	misc_otherPhosphate
VII	Bra007821	26	misc.plastocyanin-like	misc_otherPhosphate
VII	Bra005241	26	misc.UDP glucosyl and glucoronyl transferases	misc_otherPhosphate
VII	Bra012324	26	misc.UDP glucosyl and glucoronyl transferases	misc_otherPhosphate
VII	Bra027039	26	misc.protease inhibitor/seed storage/lipid transfer protein (LTP) family protein	misc_otherPhosphate
VII	Bra027039	26	misc.protease inhibitor/seed storage/lipid transfer protein (LTP) family protein	misc_otherPhosphate
VII	Bra009580	26	misc.short chain dehydrogenase/reductase (SDR)	misc_otherPhosphate
VII	Bra017092	26	misc.GCN5-related N-acetyltransferase	misc_otherPhosphate
VII	Bra037954	26	misc.GDSL-motif lipase	misc_otherPhosphate
VII	Bra037954	26	misc.GDSL-motif lipase	misc_otherPhosphate
VII	Bra030155	26	misc.GDSL-motif lipase	misc_otherPhosphate
VII	Bra037651	26	misc.gluco-, galacto- and mannosidases	misc_otherPhosphate
VII	Bra039825	26	misc.gluco-, galacto- and mannosidases	misc_otherPhosphate
VII	Bra039825	26	misc.gluco-, galacto- and mannosidases	misc_otherPhosphate
VII	Bra008377	26	misc.acyl transferases	misc_otherPhosphate
VII	Bra003680	26	misc.O-methyl transferases	misc_otherPhosphate
VII	Bra018399	26	misc.nitrilases, *nitrile lyases, berberine bridge enzymes, reticuline oxidases, troponine reductases	misc_otherPhosphate
VII	Bra018399	26	misc.nitrilases, *nitrile lyases, berberine bridge enzymes, reticuline oxidases, troponine reductases	misc_otherPhosphate
VII	Bra035006	26	misc.nitrilases, *nitrile lyases, berberine bridge enzymes, reticuline oxidases, troponine reductases	misc_otherPhosphate
VII	Bra012899	26	misc.nitrilases, *nitrile lyases, berberine bridge enzymes, reticuline oxidases, troponine reductases	misc_otherPhosphate

VII	Bra012899	26	misc.nitrilases, *nitrile lyases, berberine bridge enzymes, reticuline oxidases, troponine reductases	misc_otherPhosphate
VII	Bra031277	27	RNA.transcription	RNA
VII	Bra037216	27	RNA.regulation of transcription	RNA
VII	Bra037216	27	RNA.regulation of transcription	RNA
VII	Bra012047	27	RNA.RNA binding	RNA
VII	Bra012047	27	RNA.RNA binding	RNA
VII	Bra003136	27	RNA.RNA binding	RNA
VII	Bra030265	27	RNA.RNA binding	RNA
VII	Bra025205	27	RNA.RNA binding	RNA
VII	Bra030284	27	RNA.RNA binding	RNA
VII	Bra031210	27	RNA.RNA binding	RNA
VII	Bra031210	27	RNA.RNA binding	RNA
VII	Bra036851	28	DNA.unspecified	DNA
VII	Bra038866	28	DNA.unspecified	DNA
VII	Bra037033	29	protein.postranslational modification	protein
VII	Bra013340	29	protein.postranslational modification	protein
VII	Bra002661	29	protein.postranslational modification	protein
VII	Bra002661	29	protein.postranslational modification	protein
VII	Bra009354	29	protein.postranslational modification	protein
VII	Bra009569	29	protein.postranslational modification	protein
VII	Bra028865	29	protein.postranslational modification	protein
VII	Bra029095	29	protein.postranslational modification	protein
VII	Bra028912	29	protein.postranslational modification	protein
VII	Bra025209	29	protein.postranslational modification	protein
VII	Bra029837	29	protein.degradation	protein
VII	Bra006895	29	protein.folding	protein
VII	Bra006895	29	protein.folding	protein
VII	Bra035591	30	signalling.in sugar and nutrient physiology	signalling
VII	Bra004991	30	signalling.light	signalling
VII	Bra004991	30	signalling.light	signalling
VII	Bra017035	30	signalling.light	signalling
VII	Bra004711	30	signalling.light	signalling
VII	Bra004711	30	signalling.light	signalling
VII	Bra001886	30	signalling.light	signalling
VII	Bra001886	30	signalling.light	signalling
VII	Bra023798	30	signalling.light	signalling
VII	Bra033911	30	signalling.light	signalling
VII	Bra000165	30	signalling.light	signalling
VII	Bra000165	30	signalling.light	signalling
VII	Bra032945	30	signalling.light	signalling
VII	Bra032945	30	signalling.light	signalling
VII	Bra028144	30	signalling.calcium	signalling
VII	Bra031157	30	signalling.calcium	signalling
VII	Bra036140	30	signalling.calcium	signalling
VII	Bra033898	30	signalling.calcium	signalling
VII	Bra013539	30	signalling.calcium	signalling
VII	Bra039845	30	signalling.calcium	signalling
VII	Bra028784	30	signalling.G-proteins	signalling
VII	Bra003739	30	signalling.G-proteins	signalling
VII	Bra014355	30	signalling.G-proteins	signalling
VII	Bra038676	30	signalling.G-proteins	signalling

VII	Bra025931	30	signalling.G-proteins	signalling
VII	Bra024384	30	signalling.14-3-3 proteins	signalling
VII	Bra037560	31	cell.organisation	cell
VII	Bra020572	31	cell.organisation	cell
VII	Bra008619	31	cell.vesicle transport	cell
VII	Bra036287	31	cell.vesicle transport	cell
VII	Bra015548	33	development.unspecified	development
VII	Bra003190	33	development.unspecified	development
VII	Bra012052	34	transport.peptides and oligopeptides	transport
VII	Bra018096	34	transport.peptides and oligopeptides	transport
VII	Bra016986	34	transport.potassium	transport
VII	Bra002001	34	transport.sugars	transport
VII	Bra015673	34	transport.amino acids	transport
VII	Bra009122	34	transport.amino acids	transport
VII	Bra037814	34	transport.misc	transport
VII	Bra007887	35	not assigned.unknown	not_assigned
VII	Bra007150	35	not assigned.unknown	not_assigned
VII	Bra026701	35	not assigned.unknown	not_assigned
VII	Bra011767	35	not assigned.unknown	not_assigned
VII	Bra004554	35	not assigned.unknown	not_assigned
VII	Bra019235	35	not assigned.unknown	not_assigned
VII	Bra021220	35	not assigned.unknown	not_assigned
VII	Bra021222	35	not assigned.unknown	not_assigned
VII	Bra007905	35	not assigned.unknown	not_assigned
VII	Bra025707	35	not assigned.unknown	not_assigned
VII	Bra015813	35	not assigned.unknown	not_assigned
VII	Bra019262	35	not assigned.unknown	not_assigned
VII	Bra011196	35	not assigned.unknown	not_assigned
VII	Bra033929	35	not assigned.unknown	not_assigned
VII	Bra023241	35	not assigned.unknown	not_assigned
VII	Bra020971	35	not assigned.unknown	not_assigned
VII	Bra011521	35	not assigned.unknown	not_assigned
VII	Bra035419	35	not assigned.unknown	not_assigned
VII	Bra017619	35	not assigned.unknown	not_assigned
VII	Bra033648	35	not assigned.unknown	not_assigned
VII	Bra019261	35	not assigned.unknown	not_assigned
VII	Bra040737	35	not assigned.unknown	not_assigned
VII	Bra013091	35	not assigned.unknown	not_assigned
VII	Bra013091	35	not assigned.unknown	not_assigned
VII	Bra036168	35	not assigned.unknown	not_assigned
VII	Bra000274	35	not assigned.unknown	not_assigned
VII	Bra025385	35	not assigned.unknown	not_assigned
VII	Bra020090	35	not assigned.unknown	not_assigned
VII	Bra031967	35	not assigned.unknown	not_assigned
VII	Bra037039	35	not assigned.unknown	not_assigned
VII	Bra004812	35	not assigned.unknown	not_assigned
VII	Bra019069	35	not assigned.unknown	not_assigned
VII	Bra018486	35	not assigned.unknown	not_assigned
VII	Bra000651	35	not assigned.unknown	not_assigned
VII	Bra030753	35	not assigned.unknown	not_assigned
VII	Bra036730	35	not assigned.unknown	not_assigned
VII	Bra037731	35	not assigned.unknown	not_assigned

VII	Bra040182	35	not assigned.unknown	not_assigned
VII	Bra031873	35	not assigned.unknown	not_assigned
VII	Bra026702	35	not assigned.unknown	not_assigned
VII	Bra009790	35	not assigned.unknown	not_assigned
VII	Bra009790	35	not assigned.unknown	not_assigned
VII	Bra001367	35	not assigned.unknown	not_assigned
VII	Bra031046	35	not assigned.unknown	not_assigned
VII	Bra033870	35	not assigned.unknown	not_assigned
VII	Bra020540	35	not assigned.unknown	not_assigned
VII	Bra020540	35	not assigned.unknown	not_assigned
VII	Bra020615	35	not assigned.unknown	not_assigned
VII	Bra035898	35	not assigned.unknown	not_assigned
VII	Bra019601	35	not assigned.unknown	not_assigned
VII	Bra032721	35	not assigned.unknown	not_assigned
VII	Bra039083	35	not assigned.unknown	not_assigned
VII	Bra013746	35	not assigned.unknown	not_assigned
VII	Bra016055	35	not assigned.unknown	not_assigned
VII	Bra041048	35	not assigned.unknown	not_assigned
VII	Bra002975	35	not assigned.unknown	not_assigned
VII	Bra036021	35	not assigned.unknown	not_assigned
VII	Bra019466	35	not assigned.unknown	not_assigned
VII	Bra019828	35	not assigned.unknown	not_assigned
VII	Bra023324	35	not assigned.unknown	not_assigned
VII	Bra029409	35	not assigned.unknown	not_assigned
VII	Bra016985	35	not assigned.unknown	not_assigned
VII	Bra015808	35	not assigned.unknown	not_assigned
VII	Bra004519	35	not assigned.unknown	not_assigned
VII	Bra040903	35	not assigned.unknown	not_assigned
VII	Bra040903	35	not assigned.unknown	not_assigned
VII	Bra010199	35	not assigned.unknown	not_assigned
VII	Bra005152	35	not assigned.unknown	not_assigned
VII	Bra040060	35	not assigned.unknown	not_assigned
VII	Bra011339	35	not assigned.unknown	not_assigned
VII	Bra032790	35	not assigned.unknown	not_assigned
VII	Bra004066	35	not assigned.unknown	not_assigned
VII	Bra029848	35	not assigned.unknown	not_assigned
VII	Bra033462	35	not assigned.unknown	not_assigned
VII	Bra031968	35	not assigned.unknown	not_assigned
VII	Bra028906	1	PS.lightreaction.photosystem II.LHC-II	Photosynthesis
VII	Bra033590	1	PS.calvin cycle.aldolase	Photosynthesis
VII	Bra016757	10	cell wall.precursor synthesis.UGE	cell_wall
VII	Bra019264	10	cell wall.degradation.pectate lyases and polygalacturonases	cell_wall
VII	Bra029614	10	cell wall.degradation.pectate lyases and polygalacturonases	cell_wall
VII	Bra014550	10	cell wall.pectin*esterases.PME	cell_wall
VII	Bra000321	11	lipid metabolism.FA synthesis and FA elongation.ACP desaturase	lipid_metabolism
VII	Bra027263	11	lipid metabolism.lipid degradation.beta-oxidation.multifunctional	lipid_metabolism
VII	Bra007610	13	amino acid metabolism.degradation.aromatic aa.tyrosine	amino_acid_metabolism
VII	Bra007610	13	amino acid metabolism.degradation.aromatic aa.tyrosine	amino_acid_metabolism
VII	Bra020813	13	amino acid metabolism.degradation.aromatic aa.tyrosine	amino_acid_metabolism
VII	Bra002468	16	secondary metabolism.isoprenoids.non-mevalonate pathway.HDS	secondary_metabolism
VII	Bra011049	16	secondary metabolism.N misc.alkaloid-like	secondary_metabolism
VII	Bra018623	16	secondary metabolism.N misc.alkaloid-like	secondary_metabolism

VII	Bra003938	17	hormone metabolism.auxin.signal transduction	hormone_metabolism
VII	Bra003044	17	hormone metabolism.auxin.induced-regulated-responsive-activated	hormone_metabolism
VII	Bra026044	17	hormone metabolism.auxin.induced-regulated-responsive-activated	hormone_metabolism
VII	Bra016508	17	hormone metabolism.brassinosteroid.signal transduction.BZR	hormone_metabolism
VII	Bra035640	17	hormone metabolism.cytokinin.synthesis-degradation	hormone_metabolism
VII	Bra035640	17	hormone metabolism.cytokinin.synthesis-degradation	hormone_metabolism
VII	Bra021672	17	hormone metabolism.ethylene.synthesis-degradation	hormone_metabolism
VII	Bra013480	17	hormone metabolism.ethylene.induced-regulated-responsive-activated	hormone_metabolism
VII	Bra038796	17	hormone metabolism.ethylene.induced-regulated-responsive-activated	hormone_metabolism
VII	Bra012788	17	hormone metabolism.jasmonate.synthesis-degradation.allene oxidase synthase	hormone_metabolism
VII	Bra026230	2	major CHO metabolism.degradation.starch.starch cleavage	major_CHOmetabolism
VII	Bra039414	20	stress.biotic.PR-proteins.plant defensins	stress
VII	Bra036137	20	stress.biotic.PR-proteins.proteinase inhibitors.trypsin inhibitor	stress
VII	Bra016073	20	stress.biotic.PR-proteins.proteinase inhibitors.trypsin inhibitor	stress
VII	Bra016073	20	stress.biotic.PR-proteins.proteinase inhibitors.trypsin inhibitor	stress
VII	Bra002461	20	stress.abiotic.heat	stress
VII	Bra010049	20	stress.abiotic.heat	stress
VII	Bra011735	20	stress.abiotic.heat	stress
VII	Bra011185	20	stress.abiotic.heat	stress
VII	Bra013774	20	stress.abiotic.heat	stress
VII	Bra013774	20	stress.abiotic.heat	stress
VII	Bra013026	20	stress.abiotic.heat	stress
VII	Bra032002	20	stress.abiotic.cold	stress
VII	Bra013374	20	stress.abiotic.drought/salt	stress
VII	Bra014401	20	stress.abiotic.unspecified	stress
VII	Bra004295	20	stress.abiotic.unspecified	stress
VII	Bra028600	20	stress.abiotic.unspecified	stress
VII	Bra005853	20	stress.abiotic.unspecified	stress
VII	Bra034800	20	stress.abiotic.unspecified	stress
VII	Bra021440	20	stress.abiotic.unspecified	stress
VII	Bra021440	20	stress.abiotic.unspecified	stress
VII	Bra004395	23	nucleotide metabolism.synthesis.pyrimidine.carbamoyl phosphate synthetase	nucleotide_metabolism
VII	Bra035260	23	nucleotide metabolism.phosphotransfer and pyrophosphatases.nucleoside diphosphate kinase	nucleotide_metabolism
VII	Bra024238	26	misc.gluco-, galacto- and mannosidases.beta-galactosidase	misc_otherPhosphate
VII	Bra024624	27	RNA.processing.splicing	RNA
VII	Bra026570	27	RNA.processing.ribonucleases	RNA
VII	Bra037151	27	RNA.regulation of transcription.C2H2 zinc finger family	RNA
VII	Bra040220	27	RNA.regulation of transcription.C3H zinc finger family	RNA
VII	Bra017473	27	RNA.regulation of transcription.CCAAT box binding factor family, HAP3	RNA
VII	Bra018204	27	RNA.regulation of transcription.G2-like transcription factor family, GARP	RNA
VII	Bra018204	27	RNA.regulation of transcription.G2-like transcription factor family, GARP	RNA
VII	Bra039340	27	RNA.regulation of transcription.G2-like transcription factor family, GARP	RNA
VII	Bra006051	27	RNA.regulation of transcription.MADS box transcription factor family	RNA
VII	Bra006051	27	RNA.regulation of transcription.MADS box transcription factor family	RNA
VII	Bra039503	27	RNA.regulation of transcription.MYB-related transcription factor family	RNA
VII	Bra009338	27	RNA.regulation of transcription.TCP transcription factor family	RNA
VII	Bra026509	27	RNA.regulation of transcription.AP2/EREBP, APETALA2/Ethylene-responsive element binding protein family	RNA
VII	Bra016367	27	RNA.regulation of transcription.AP2/EREBP, APETALA2/Ethylene-responsive element binding protein family	RNA
VII	Bra034866	27	RNA.regulation of transcription.Trihelix, Triple-Helix transcription factor family	RNA
VII	Bra011282	27	RNA.regulation of transcription.WRKY domain transcription factor family	RNA

VII	Bra032174	27	RNA.regulation of transcription.WRKY domain transcription factor family	RNA
VII	Bra016959	27	RNA.regulation of transcription.bZIP transcription factor family	RNA
VII	Bra003320	27	RNA.regulation of transcription.bZIP transcription factor family	RNA
VII	Bra003320	27	RNA.regulation of transcription.bZIP transcription factor family	RNA
VII	Bra030248	27	RNA.regulation of transcription.AtSR Transcription Factor family	RNA
VII	Bra023958	27	RNA.regulation of transcription.Aux/IAA family	RNA
VII	Bra035742	27	RNA.regulation of transcription.JUMONJI family	RNA
VII	Bra035742	27	RNA.regulation of transcription.JUMONJI family	RNA
VII	Bra004489	27	RNA.regulation of transcription.bHLH,Basic Helix-Loop-Helix family	RNA
VII	Bra011540	27	RNA.regulation of transcription.bHLH,Basic Helix-Loop-Helix family	RNA
VII	Bra012435	27	RNA.regulation of transcription.bHLH,Basic Helix-Loop-Helix family	RNA
VII	Bra016299	27	RNA.regulation of transcription.bHLH,Basic Helix-Loop-Helix family	RNA
VII	Bra012964	27	RNA.regulation of transcription.Psudo ARR transcription factor family	RNA
VII	Bra004940	27	RNA.regulation of transcription.putative transcription regulator	RNA
VII	Bra020709	27	RNA.regulation of transcription.C2C2(Zn) CO-like, Constans-like zinc finger family	RNA
VII	Bra037499	27	RNA.regulation of transcription.C2C2(Zn) CO-like, Constans-like zinc finger family	RNA
VII	Bra037499	27	RNA.regulation of transcription.C2C2(Zn) CO-like, Constans-like zinc finger family	RNA
VII	Bra001264	27	RNA.regulation of transcription.C2C2(Zn) CO-like, Constans-like zinc finger family	RNA
VII	Bra001822	27	RNA.regulation of transcription.C2C2(Zn) DOF zinc finger family	RNA
VII	Bra001925	27	RNA.regulation of transcription.C2C2(Zn) GATA transcription factor family	RNA
VII	Bra007070	27	RNA.regulation of transcription.unclassified	RNA
VII	Bra007070	27	RNA.regulation of transcription.unclassified	RNA
VII	Bra038619	27	RNA.regulation of transcription.unclassified	RNA
VII	Bra006084	28	DNA.synthesis/chromatin structure.histone	DNA
VII	Bra007023	28	DNA.synthesis/chromatin structure.histone	DNA
VII	Bra000481	28	DNA.synthesis/chromatin structure.histone	DNA
VII	Bra018691	28	DNA.synthesis/chromatin structure.histone	DNA
VII	Bra011664	29	protein.synthesis.ribosomal protein.prokaryotic.chloroplast.50S subunit.L2	protein
VII	Bra002779	29	protein.synthesis.ribosomal protein.prokaryotic.unknown organellar.30S subunit.S16	protein
VII	Bra023052	29	protein.synthesis.ribosomal protein.eukaryotic.40S subunit.S14	protein
VII	Bra002045	29	protein.synthesis.ribosomal protein.eukaryotic.40S subunit.S14	protein
VII	Bra005887	29	protein.synthesis.ribosomal protein.eukaryotic.40S subunit.S17	protein
VII	Bra035939	29	protein.synthesis.ribosomal protein.eukaryotic.40S subunit.S19	protein
VII	Bra038226	29	protein.synthesis.ribosomal protein.eukaryotic.40S subunit.S20	protein
VII	Bra011551	29	protein.synthesis.ribosomal protein.eukaryotic.40S subunit.S3A	protein
VII	Bra017204	29	protein.synthesis.ribosomal protein.eukaryotic.60S subunit.L12	protein
VII	Bra009940	29	protein.synthesis.ribosomal protein.eukaryotic.60S subunit.L18	protein
VII	Bra036611	29	protein.synthesis.ribosomal protein.eukaryotic.60S subunit.L18	protein
VII	Bra024577	29	protein.synthesis.ribosomal protein.eukaryotic.60S subunit.L27A	protein
VII	Bra018487	29	protein.synthesis.ribosomal protein.eukaryotic.60S subunit.L7A	protein
VII	Bra024770	29	protein.synthesis.ribosomal protein.eukaryotic.60S subunit.P0	protein
VII	Bra008928	29	protein.synthesis.initiation	protein
VII	Bra014633	29	protein.synthesis.initiation	protein
VII	Bra018242	29	protein.synthesis.elongation	protein
VII	Bra010782	29	protein.synthesis.elongation	protein
VII	Bra030765	29	protein.targeting.nucleus	protein
VII	Bra040927	29	protein.targeting.mitochondria	protein
VII	Bra018197	29	protein.targeting.chloroplast	protein
VII	Bra040958	29	protein.targeting.secretory pathway.unspecified	protein
VII	Bra007433	29	protein.postranslational modification.kinase.receptor like cytoplasmatic kinase VII	protein
VII	Bra021105	29	protein.degradation.ubiquitin.E2	protein
VII	Bra006790	29	protein.degradation.ubiquitin.ubiquitin protease	protein

VII	Bra033420	29	protein.degradation.ubiquitin.ubiquitin	protein
VII	Bra033732	29	protein.degradation.ubiquitin.E3.RING	protein
VII	Bra003450	29	protein.degradation.ubiquitin.E3.RING	protein
VII	Bra035785	29	protein.degradation.ubiquitin.E3.RING	protein
VII	Bra007494	29	protein.degradation.ubiquitin.E3.SCF.FBOX	protein
VII	Bra019893	29	protein.degradation.ubiquitin.E3.SCF.FBOX	protein
VII	Bra025177	29	protein.degradation.ubiquitin.E3.SCF.FBOX	protein
VII	Bra041154	29	protein.degradation.ubiquitin.E3.SCF.FBOX	protein
VII	Bra020764	29	protein.degradation.ubiquitin.E3.BTB/POZ Cullin3.BTB/POZ	protein
VII	Bra040939	29	protein.degradation.serine protease	protein
VII	Bra001359	29	protein.degradation.serine protease	protein
VII	Bra001359	29	protein.degradation.serine protease	protein
VII	Bra032681	29	protein.degradation.serine protease	protein
VII	Bra041014	3	minor CHO metabolism.myo-inositol.myo inositol oxygenases	minor_CHOmetabolism
VII	Bra000908	30	signalling.in sugar and nutrient physiology	signalling
VII	Bra019737	30	signalling.receptor kinases.leucine rich repeat XI	signalling
VII	Bra003549	30	signalling.receptor kinases.leucine rich repeat XI	signalling
VII	Bra019345	30	signalling.receptor kinases.DUF 26	signalling
VII	Bra009723	31	cell.division.plastid	cell
VII	Bra037607	31	cell.cell death.plants	cell
VII	Bra019307	34	transport.Major Intrinsic Proteins.PIP	transport
VII	Bra019307	34	transport.Major Intrinsic Proteins.PIP	transport
VII	Bra001626	34	transport.Major Intrinsic Proteins.TIP	transport
VII	Bra003906	34	transport.sugars.sucrose	transport
VII	Bra030775	7	OPP.oxidative PP.G6PD	OPP
VII	Bra030775	7	OPP.oxidative PP.G6PD	OPP
VII	Bra019748	7	OPP.non-reductive PP.transaldolase	OPP
VII	Bra024217	9	mitochondrial electron transport / ATP synthesis.NADH-DH.type II.external	ATP_synthesis
Cluster	BraID	Bin	annotation	pathway
IX	Bra001747	13	amino acid metabolism.synthesis.serine-glycine-cysteine group.serine.phosphoglycerate dehydrogenase	amino_acid_metabolism
IX	Bra029140	13	amino acid metabolism.synthesis.aspartate family.lysine.dihydrodipicolinate reductase	amino_acid_metabolism
IX	Bra025383	13	amino acid metabolism.synthesis.aromatic aa.phenylalanine and tyrosine.chorismate mutase	amino_acid_metabolism
IX	Bra011140	13	amino acid metabolism.synthesis.aspartate family.threonine.threonine synthase	amino_acid_metabolism
IX	Bra012371	13	amino acid metabolism.synthesis.central amino acid metabolism.alanine.alanine aminotransferase	amino_acid_metabolism
IX	Bra016353	13	amino acid metabolism.synthesis.central amino acid metabolism.alanine.alanine aminotransferase	amino_acid_metabolism
IX	Bra014184	9	mitochondrial electron transport / ATP synthesis.cytochrome c	ATP_synthesis
IX	Bra030676	9	mitochondrial electron transport / ATP synthesis.NADH-DH.type II.internal matrix	ATP_synthesis
IX	Bra006835	24	Biodegradation of Xenobiotics.lactoylglutathione lyase	Biodegradation_Xenobiotics
IX	Bra018149	31	cell.organisation	cell
IX	Bra013772	31	cell.organisation	cell
IX	Bra006525	31	cell.organisation	cell
IX	Bra007655	31	cell.cycle.peptidylprolyl isomerase	cell
IX	Bra000401	31	cell.organisation	cell
IX	Bra031315	31	cell.cycle	cell
IX	Bra031315	31	cell.cycle	cell
IX	Bra028271	31	cell.organisation	cell
IX	Bra028271	31	cell.organisation	cell
IX	Bra004932	31	cell.organisation	cell

IX	Bra025156	31	cell.organisation	cell
IX	Bra004932	31	cell.organisation	cell
IX	Bra008130	31	cell.cycle.peptidylprolyl isomerase	cell
IX	Bra008130	31	cell.cycle.peptidylprolyl isomerase	cell
IX	Bra021797	10	cell wall.cellulose synthesis.cellulose synthase	cell_wall
IX	Bra027148	10	cell wall.degradation.pectate lyases and polygalacturonases	cell_wall
IX	Bra024848	10	cell wall.modification	cell_wall
IX	Bra019253	10	cell wall.cellulose synthesis.cellulose synthase	cell_wall
IX	Bra018372	18	Co-factor and vitamine metabolism.thiamine	Co-factor_vitamine_metabolism
IX	Bra022742	18	Co-factor and vitamine metabolism.thiamine	Co-factor_vitamine_metabolism
IX	Bra022742	18	Co-factor and vitamine metabolism.thiamine	Co-factor_vitamine_metabolism
IX	Bra031496	18	Co-factor and vitamine metabolism.folate & vitamine K.DHNA Phytyltransferase	Co-factor_vitamine_metabolism
IX	Bra029007	18	Co-factor and vitamine metabolism.thiamine	Co-factor_vitamine_metabolism
IX	Bra011573	33	development.unspecified	development
IX	Bra004159	33	development.unspecified	development
IX	Bra008880	33	development.unspecified	development
IX	Bra040403	33	development.unspecified	development
IX	Bra037385	33	development.unspecified	development
IX	Bra027932	33	development.unspecified	development
IX	Bra028411	28	DNA.repair	DNA
IX	Bra016776	28	DNA.repair	DNA
IX	Bra002264	28	DNA.repair	DNA
IX	Bra026426	4	glycolysis.cytosolic branch.aldolase	glycolysis
IX	Bra026426	4	glycolysis.cytosolic branch.aldolase	glycolysis
IX	Bra016184	4	glycolysis.plastid branch.phosphoglucomutase (PGM)	glycolysis
IX	Bra009397	6	gluconeogenesis.Malate DH	glyoxylate_cycle
IX	Bra024530	17	hormone metabolism.gibberelin.induced-regulated-responsive-activated	hormone_metabolism
IX	Bra003526	17	hormone metabolism.jasmonate.synthesis-degradation.lipoxygenase	hormone_metabolism
IX	Bra003526	17	hormone metabolism.jasmonate.synthesis-degradation.lipoxygenase	hormone_metabolism
IX	Bra004057	17	hormone metabolism.jasmonate.synthesis-degradation.lipoxygenase	hormone_metabolism
IX	Bra022535	17	hormone metabolism.jasmonate.synthesis-degradation.lipoxygenase	hormone_metabolism
IX	Bra034897	17	hormone metabolism.auxin.induced-regulated-responsive-activated	hormone_metabolism
IX	Bra008162	17	hormone metabolism.gibberelin.induced-regulated-responsive-activated	hormone_metabolism
IX	Bra008162	17	hormone metabolism.gibberelin.induced-regulated-responsive-activated	hormone_metabolism
IX	Bra009358	17	hormone metabolism.ethylene.synthesis-degradation	hormone_metabolism
IX	Bra001345	11	lipid metabolism.lipid transfer proteins etc	lipid_metabolism
IX	Bra027203	11	lipid metabolism.FA desaturation.desaturase	lipid_metabolism
IX	Bra020322	11	lipid metabolism.lipid transfer proteins etc	lipid_metabolism
IX	Bra014005	2	major CHO metabolism.synthesis.sucrose.FBPase	major_CHOmetabolism
IX	Bra014005	2	major CHO metabolism.synthesis.sucrose.FBPase	major_CHOmetabolism
IX	Bra026230	2	major CHO metabolism.degradation.starch.starch cleavage	major_CHOmetabolism
IX	Bra037953	15	metal handling.acquisition	metal_handling
IX	Bra040643	15	metal handling.binding, chelation and storage	metal_handling
IX	Bra029134	3	minor CHO metabolism.others	minor_CHOmetabolism
IX	Bra018941	26	misc.myrosinases-lectin-jacalin	misc_otherPhosphate
IX	Bra018941	26	misc.myrosinases-lectin-jacalin	misc_otherPhosphate
IX	Bra020376	26	misc.nitrilases, *nitrile lyases, berberine bridge enzymes, reticuline oxidases, troponine reductases	misc_otherPhosphate
IX	Bra018942	26	misc.myrosinases-lectin-jacalin	misc_otherPhosphate
IX	Bra016683	26	misc.acid and other phosphatases	misc_otherPhosphate
IX	Bra007698	26	misc.gluco-, galacto- and mannosidases	misc_otherPhosphate

IX	Bra018405	26	misc.nitrilases, *nitrile lyases, berberine bridge enzymes, reticuline oxidases, troponine reductases	misc_otherPhosphate
IX	Bra030683	26	misc.nitrilases, *nitrile lyases, berberine bridge enzymes, reticuline oxidases, troponine reductases	misc_otherPhosphate
IX	Bra030683	26	misc.nitrilases, *nitrile lyases, berberine bridge enzymes, reticuline oxidases, troponine reductases	misc_otherPhosphate
IX	Bra026402	26	misc.GDSL-motif lipase	misc_otherPhosphate
IX	Bra010104	26	misc.rhodanese	misc_otherPhosphate
IX	Bra016889	26	misc.rhodanese	misc_otherPhosphate
IX	Bra013013	26	misc.rhodanese	misc_otherPhosphate
IX	Bra012492	26	misc.acid and other phosphatases	misc_otherPhosphate
IX	Bra006756	26	misc.gluco-, galacto- and mannosidases	misc_otherPhosphate
IX	Bra031290	26	misc.UDP glucosyl and glucoronyl transferases	misc_otherPhosphate
IX	Bra027168	26	misc.GDSL-motif lipase	misc_otherPhosphate
IX	Bra039971	26	misc.nitrilases, *nitrile lyases, berberine bridge enzymes, reticuline oxidases, troponine reductases	misc_otherPhosphate
IX	Bra015038	35	not assigned.unknown	not_assigned
IX	Bra015038	35	not assigned.unknown	not_assigned
IX	Bra013804	35	not assigned.unknown	not_assigned
IX	Bra012282	35	not assigned.unknown	not_assigned
IX	Bra030268	35	not assigned.unknown	not_assigned
IX	Bra032150	35	not assigned.unknown	not_assigned
IX	Bra032150	35	not assigned.unknown	not_assigned
IX	Bra014130	35	not assigned.unknown	not_assigned
IX	Bra018723	35	not assigned.unknown	not_assigned
IX	Bra032252	35	not assigned.unknown	not_assigned
IX	Bra025039	35	not assigned.unknown	not_assigned
IX	Bra025526	35	not assigned.unknown	not_assigned
IX	Bra027662	35	not assigned.unknown	not_assigned
IX	Bra037832	35	not assigned.unknown	not_assigned
IX	Bra016991	35	not assigned.unknown	not_assigned
IX	Bra005855	35	not assigned.unknown	not_assigned
IX	Bra016436	35	not assigned.unknown	not_assigned
IX	Bra027680	35	not assigned.unknown	not_assigned
IX	Bra000901	35	not assigned.unknown	not_assigned
IX	Bra034898	35	not assigned.unknown	not_assigned
IX	Bra010126	35	not assigned.unknown	not_assigned
IX	Bra010126	35	not assigned.unknown	not_assigned
IX	Bra010818	35	not assigned.unknown	not_assigned
IX	Bra026325	35	not assigned.unknown	not_assigned
IX	Bra023273	35	not assigned.unknown	not_assigned
IX	Bra029285	35	not assigned.unknown	not_assigned
IX	Bra005211	35	not assigned.unknown	not_assigned
IX	Bra011918	35	not assigned.unknown	not_assigned
IX	Bra015897	35	not assigned.unknown	not_assigned
IX	Bra015897	35	not assigned.unknown	not_assigned
IX	Bra037462	35	not assigned.unknown	not_assigned
IX	Bra005964	35	not assigned.unknown	not_assigned
IX	Bra005964	35	not assigned.unknown	not_assigned
IX	Bra038904	35	not assigned.unknown	not_assigned
IX	Bra014139	35	not assigned.unknown	not_assigned
IX	Bra017838	35	not assigned.unknown	not_assigned
IX	Bra035213	35	not assigned.unknown	not_assigned

IX	Bra012022	35	not assigned.unknown	not_assigned
IX	Bra012722	35	not assigned.unknown	not_assigned
IX	Bra037997	35	not assigned.unknown	not_assigned
IX	Bra020809	35	not assigned.unknown	not_assigned
IX	Bra030380	35	not assigned.unknown	not_assigned
IX	Bra008166	35	not assigned.unknown	not_assigned
IX	Bra033642	35	not assigned.unknown	not_assigned
IX	Bra020448	35	not assigned.unknown	not_assigned
IX	Bra009299	35	not assigned.unknown	not_assigned
IX	Bra001281	35	not assigned.unknown	not_assigned
IX	Bra009299	35	not assigned.unknown	not_assigned
IX	Bra037380	35	not assigned.unknown	not_assigned
IX	Bra026733	35	not assigned.unknown	not_assigned
IX	Bra025664	35	not assigned.unknown	not_assigned
IX	Bra031197	35	not assigned.unknown	not_assigned
IX	Bra034050	35	not assigned.unknown	not_assigned
IX	Bra040103	35	not assigned.unknown	not_assigned
IX	Bra031902	35	not assigned.unknown	not_assigned
IX	Bra031784	35	not assigned.unknown	not_assigned
IX	Bra026087	35	not assigned.unknown	not_assigned
IX	Bra021447	35	not assigned.unknown	not_assigned
IX	Bra015164	35	not assigned.unknown	not_assigned
IX	Bra010643	35	not assigned.unknown	not_assigned
IX	Bra010643	35	not assigned.unknown	not_assigned
IX	Bra031662	35	not assigned.unknown	not_assigned
IX	Bra015132	35	not assigned.unknown	not_assigned
IX	Bra032674	35	not assigned.unknown	not_assigned
IX	Bra037177	35	not assigned.unknown	not_assigned
IX	Bra008036	35	not assigned.unknown	not_assigned
IX	Bra017529	35	not assigned.unknown	not_assigned
IX	Bra024952	23	nucleotide metabolism.salvage.NUDIX hydrolases	nucleotide_metabolism
IX	Bra019796	23	nucleotide metabolism.salvage.NUDIX hydrolases	nucleotide_metabolism
IX	Bra000144	1	PS.calvin cycle.rubisco interacting	Photosynthesis
IX	Bra000144	1	PS.calvin cycle.rubisco interacting	Photosynthesis
IX	Bra031937	1	PS.lightreaction.photosystem I.PSI polypeptide subunits	Photosynthesis
IX	Bra027204	1	PS.lightreaction.cyclic electron flow-chlororespiration	Photosynthesis
IX	Bra021172	1	PS.lightreaction.other electron carrier (ox/red).ferredoxin	Photosynthesis
IX	Bra032939	1	PS.calvin cycle.GAP	Photosynthesis
IX	Bra028906	1	PS.lightreaction.photosystem II.LHC-II	Photosynthesis
IX	Bra033412	1	PS.lightreaction.photosystem II.PSII polypeptide subunits	Photosynthesis
IX	Bra003624	1	PS.lightreaction.photosystem II.PSII polypeptide subunits	Photosynthesis
IX	Bra010350	1	PS.lightreaction.photosystem I.PSI polypeptide subunits	Photosynthesis
IX	Bra035533	1	PS.lightreaction.photosystem II.PSII polypeptide subunits	Photosynthesis
IX	Bra030722	1	PS.lightreaction.photosystem I.PSI polypeptide subunits	Photosynthesis
IX	Bra010774	1	PS.lightreaction.photosystem I.PSI polypeptide subunits	Photosynthesis
IX	Bra032384	1	PS.lightreaction.photosystem I.PSI polypeptide subunits	Photosynthesis
IX	Bra018415	1	PS.lightreaction.other electron carrier (ox/red).ferredoxin	Photosynthesis
IX	Bra032384	1	PS.lightreaction.photosystem I.PSI polypeptide subunits	Photosynthesis
IX	Bra037104	1	PS.lightreaction.photosystem I.PSI polypeptide subunits	Photosynthesis
IX	Bra010807	1	PS.lightreaction.photosystem II.LHC-II	Photosynthesis
IX	Bra030181	1	PS.lightreaction.photosystem II.LHC-II	Photosynthesis
IX	Bra034911	1	PS.lightreaction.photosystem I.PSI polypeptide subunits	Photosynthesis

IX	Bra037913	1	PS.lightreaction.photosystem II.LHC-II	Photosynthesis
IX	Bra037913	1	PS.lightreaction.photosystem II.LHC-II	Photosynthesis
IX	Bra027083	1	PS.lightreaction.photosystem II.LHC-II	Photosynthesis
IX	Bra027083	1	PS.lightreaction.photosystem II.LHC-II	Photosynthesis
IX	Bra038584	1	PS.lightreaction.photosystem II.LHC-II	Photosynthesis
IX	Bra031937	1	PS.lightreaction.photosystem I.PSI polypeptide subunits	Photosynthesis
IX	Bra000567	1	PS.lightreaction.photosystem II.PSII polypeptide subunits	Photosynthesis
IX	Bra013517	1	PS.lightreaction.photosystem II.PSII polypeptide subunits	Photosynthesis
IX	Bra015520	1	PS.lightreaction.photosystem II.PSII polypeptide subunits	Photosynthesis
IX	Bra031624	1	PS.lightreaction.photosystem I.PSI polypeptide subunits	Photosynthesis
IX	Bra031624	1	PS.lightreaction.photosystem I.PSI polypeptide subunits	Photosynthesis
IX	Bra018955	1	PS.lightreaction.photosystem I.PSI polypeptide subunits	Photosynthesis
IX	Bra031813	1	PS.lightreaction.photosystem II.PSII polypeptide subunits	Photosynthesis
IX	Bra016522	1	PS.lightreaction.photosystem II.LHC-II	Photosynthesis
IX	Bra033861	1	PS.calvin cycle.PRK	Photosynthesis
IX	Bra015530	1	PS.lightreaction.photosystem II.PSII polypeptide subunits	Photosynthesis
IX	Bra021556	1	PS.photorespiration.glycolate oxydase	Photosynthesis
IX	Bra021555	1	PS.photorespiration.glycolate oxydase	Photosynthesis
IX	Bra027338	1	PS.photorespiration.glycolate oxydase	Photosynthesis
IX	Bra012057	1	PS.lightreaction.other electron carrier (ox/red).ferredoxin reductase	Photosynthesis
IX	Bra012057	1	PS.lightreaction.other electron carrier (ox/red).ferredoxin reductase	Photosynthesis
IX	Bra009244	1	PS.calvin cycle.RPE	Photosynthesis
IX	Bra035349	1	PS.photorespiration.phosphoglycolate phosphatase	Photosynthesis
IX	Bra013653	1	PS.lightreaction.cyclic electron flow-chlororespiration	Photosynthesis
IX	Bra026948	1	PS.calvin cycle.GAP	Photosynthesis
IX	Bra000687	1	PS.lightreaction.ATP synthase.delta chain	Photosynthesis
IX	Bra018629	1	PS.lightreaction.photosystem I.PSI polypeptide subunits	Photosynthesis
IX	Bra019797	1	PS.calvin cycle.GAP	Photosynthesis
IX	Bra019797	1	PS.calvin cycle.GAP	Photosynthesis
IX	Bra026948	1	PS.calvin cycle.GAP	Photosynthesis
IX	Bra032672	1	PS.lightreaction.photosystem I.PSI polypeptide subunits	Photosynthesis
IX	Bra011884	1	PS.calvin cycle.aldolase	Photosynthesis
IX	Bra033589	1	PS.calvin cycle.aldolase	Photosynthesis
IX	Bra010717	1	PS.calvin cycle.aldolase	Photosynthesis
IX	Bra033589	1	PS.calvin cycle.aldolase	Photosynthesis
IX	Bra011884	1	PS.calvin cycle.aldolase	Photosynthesis
IX	Bra036950	1	PS.lightreaction.photosystem II.PSII polypeptide subunits	Photosynthesis
IX	Bra012083	1	PS.lightreaction.photosystem II.PSII polypeptide subunits	Photosynthesis
IX	Bra012083	1	PS.lightreaction.photosystem II.PSII polypeptide subunits	Photosynthesis
IX	Bra018954	1	PS.lightreaction.photosystem I.PSI polypeptide subunits	Photosynthesis
IX	Bra018954	1	PS.lightreaction.photosystem I.PSI polypeptide subunits	Photosynthesis
IX	Bra023176	1	PS.lightreaction.photosystem I.PSI polypeptide subunits	Photosynthesis
IX	Bra029563	1	PS.lightreaction.photosystem II.PSII polypeptide subunits	Photosynthesis
IX	Bra029563	1	PS.lightreaction.photosystem II.PSII polypeptide subunits	Photosynthesis
IX	Bra036911	1	PS.lightreaction.other electron carrier (ox/red).ferredoxin	Photosynthesis
IX	Bra027188	1	PS.lightreaction.photosystem I.PSI polypeptide subunits	Photosynthesis
IX	Bra037761	1	PS.lightreaction.photosystem I.PSI polypeptide subunits	Photosynthesis
IX	Bra005037	1	PS.lightreaction.photosystem II.PSII polypeptide subunits	Photosynthesis
IX	Bra007678	1	PS.calvin cycle	Photosynthesis
IX	Bra005347	1	PS.photorespiration.glycine cleavage.H protein	Photosynthesis
IX	Bra012962	1	PS.calvin cycle.RPE	Photosynthesis
IX	Bra012962	1	PS.calvin cycle.RPE	Photosynthesis

IX	Bra012963	1	PS.calvin cycle.RPE	Photosynthesis
IX	Bra001550	1	PS.photorespiration.glycolate oxydase	Photosynthesis
IX	Bra001551	1	PS.photorespiration.glycolate oxydase	Photosynthesis
IX	Bra005793	1	PS.photorespiration.glycolate oxydase	Photosynthesis
IX	Bra005793	1	PS.photorespiration.glycolate oxydase	Photosynthesis
IX	Bra021555	1	PS.photorespiration.glycolate oxydase	Photosynthesis
IX	Bra033861	1	PS.calvin cycle.PRK	Photosynthesis
IX	Bra007041	1	PS.calvin cycle.FBPase	Photosynthesis
IX	Bra007041	1	PS.calvin cycle.FBPase	Photosynthesis
IX	Bra010717	1	PS.calvin cycle.aldolase	Photosynthesis
IX	Bra004989	1	PS.lightreaction.photosystem II.LHC-II	Photosynthesis
IX	Bra000837	1	PS.lightreaction.cytochrome b6/f.iron sulfur subunit	Photosynthesis
IX	Bra004989	1	PS.lightreaction.photosystem II.LHC-II	Photosynthesis
IX	Bra034200	1	PS.lightreaction.cytochrome b6/f.iron sulfur subunit	Photosynthesis
IX	Bra012602	1	PS.photorespiration.glycolate oxydase	Photosynthesis
IX	Bra004275	1	PS.photorespiration.hydroxypyruvate reductase	Photosynthesis
IX	Bra004275	1	PS.photorespiration.hydroxypyruvate reductase	Photosynthesis
IX	Bra004055	1	PS.photorespiration.hydroxypyruvate reductase	Photosynthesis
IX	Bra004055	1	PS.photorespiration.hydroxypyruvate reductase	Photosynthesis
IX	Bra037207	1	PS.photorespiration.aminotransferases peroxisomal	Photosynthesis
IX	Bra015788	1	PS.lightreaction.other electron carrier (ox/red).plastocyanin	Photosynthesis
IX	Bra005037	1	PS.lightreaction.photosystem II.PSII polypeptide subunits	Photosynthesis
IX	Bra005347	1	PS.photorespiration.glycine cleavage.H protein	Photosynthesis
IX	Bra021172	1	PS.lightreaction.other electron carrier (ox/red).ferredoxin	Photosynthesis
IX	Bra027180	1	PS.lightreaction.other electron carrier (ox/red).ferredoxin	Photosynthesis
IX	Bra027180	1	PS.lightreaction.other electron carrier (ox/red).ferredoxin	Photosynthesis
IX	Bra028406	1	PS.calvin cycle.rubisco small subunit	Photosynthesis
IX	Bra028406	1	PS.calvin cycle.rubisco small subunit	Photosynthesis
IX	Bra034024	1	PS.calvin cycle.rubisco small subunit	Photosynthesis
IX	Bra021556	1	PS.photorespiration.glycolate oxydase	Photosynthesis
IX	Bra027338	1	PS.photorespiration.glycolate oxydase	Photosynthesis
IX	Bra035349	1	PS.photorespiration.phosphoglycolate phosphatase	Photosynthesis
IX	Bra017055	1	PS.calvin cycle.rubisco interacting	Photosynthesis
IX	Bra040482	1	PS.lightreaction.photosystem I.PSI polypeptide subunits	Photosynthesis
IX	Bra040482	1	PS.lightreaction.photosystem I.PSI polypeptide subunits	Photosynthesis
IX	Bra040517	1	PS.lightreaction.photosystem II.PSII polypeptide subunits	Photosynthesis
IX	Bra013148	1	PS.photorespiration.aminotransferases peroxisomal	Photosynthesis
IX	Bra013148	1	PS.photorespiration.aminotransferases peroxisomal	Photosynthesis
IX	Bra013495	29	protein.glycosylation	protein
IX	Bra014712	29	protein.synthesis.ribosomal protein.unknown.unknown	protein
IX	Bra014493	29	protein.folding	protein
IX	Bra017258	29	protein.synthesis.ribosomal protein.eukaryotic.40S subunit.S14	protein
IX	Bra033753	29	protein.synthesis.elongation	protein
IX	Bra033753	29	protein.synthesis.elongation	protein
IX	Bra015187	29	protein.degradation	protein
IX	Bra021450	29	protein.degradation.serine protease	protein
IX	Bra035916	29	protein.folding	protein
IX	Bra028001	29	protein.synthesis.release	protein
IX	Bra004247	29	protein.degradation.metalloprotease	protein
IX	Bra004247	29	protein.degradation.metalloprotease	protein
IX	Bra011719	29	protein.assembly and cofactor ligation	protein
IX	Bra038361	29	protein.synthesis.ribosomal protein.prokaryotic.unknown organellar.50S subunit.L7/L12	protein

IX	Bra038361	29	protein.synthesis.ribosomal protein.prokaryotic.unknown organellar.50S subunit.L7/L12	protein
IX	Bra017596	29	protein.folding	protein
IX	Bra017596	29	protein.folding	protein
IX	Bra029818	21	redox.heme	redox
IX	Bra000663	21	redox.ascorbate and glutathione.ascorbate	redox
IX	Bra029818	21	redox.heme	redox
IX	Bra025303	21	redox.ascorbate and glutathione.ascorbate	redox
IX	Bra025303	21	redox.ascorbate and glutathione.ascorbate	redox
IX	Bra007937	27	RNA.regulation of transcription.C2C2(Zn) CO-like, Constans-like zinc finger family	RNA
IX	Bra022574	27	RNA.regulation of transcription.bZIP transcription factor family	RNA
IX	Bra022574	27	RNA.regulation of transcription.bZIP transcription factor family	RNA
IX	Bra037312	27	RNA.regulation of transcription.bHLH,Basic Helix-Loop-Helix family	RNA
IX	Bra030635	27	RNA.regulation of transcription.C2C2(Zn) CO-like, Constans-like zinc finger family	RNA
IX	Bra003005	27	RNA.regulation of transcription.unclassified	RNA
IX	Bra030796	27	RNA.regulation of transcription.unclassified	RNA
IX	Bra031484	27	RNA.regulation of transcription.putative transcription regulator	RNA
IX	Bra017201	27	RNA.RNA binding	RNA
IX	Bra018565	27	RNA.regulation of transcription.unclassified	RNA
IX	Bra005405	27	RNA.regulation of transcription.unclassified	RNA
IX	Bra037226	27	RNA.regulation of transcription.bHLH,Basic Helix-Loop-Helix family	RNA
IX	Bra028186	27	RNA.regulation of transcription.unclassified	RNA
IX	Bra028186	27	RNA.regulation of transcription.unclassified	RNA
IX	Bra020030	27	RNA.regulation of transcription.unclassified	RNA
IX	Bra020030	27	RNA.regulation of transcription.unclassified	RNA
IX	Bra037042	16	secondary metabolism.isoprenoids.tocopherol biosynthesis.tocopherol cyclase	secondary_metabolism
IX	Bra002383	16	secondary metabolism.N misc.alkaloid-like	secondary_metabolism
IX	Bra009101	16	secondary metabolism.flavonoids.chalcones	secondary_metabolism
IX	Bra020020	16	secondary metabolism.flavonoids.dihydroflavonols	secondary_metabolism
IX	Bra020020	16	secondary metabolism.flavonoids.dihydroflavonols	secondary_metabolism
IX	Bra003009	16	secondary metabolism.phenylpropanoids.lignin biosynthesis.COMT	secondary_metabolism
IX	Bra008569	16	secondary metabolism.isoprenoids.carotenoids.phytoene synthase	secondary_metabolism
IX	Bra032666	30	signalling.light.COP9 signalosome	signalling
IX	Bra004444	30	signalling.light	signalling
IX	Bra016060	20	stress.abiotic.heat	stress
IX	Bra033070	20	stress.biotic	stress
IX	Bra004770	20	stress.biotic.PR-proteins.proteinase inhibitors.trypsin inhibitor	stress
IX	Bra038170	20	stress.abiotic.heat	stress
IX	Bra038170	20	stress.abiotic.heat	stress
IX	Bra006567	20	stress.abiotic.unspecified	stress
IX	Bra006567	20	stress.abiotic.unspecified	stress
IX	Bra002370	20	stress.abiotic.heat	stress
IX	Bra003874	20	stress.abiotic.unspecified	stress
IX	Bra003874	20	stress.abiotic.unspecified	stress
IX	Bra002316	20	stress.abiotic.unspecified	stress
IX	Bra002316	20	stress.abiotic.unspecified	stress
IX	Bra020113	20	stress.abiotic.unspecified	stress
IX	Bra020115	20	stress.abiotic.unspecified	stress
IX	Bra020113	20	stress.abiotic.unspecified	stress
IX	Bra020115	20	stress.abiotic.unspecified	stress
IX	Bra039113	8	TCA / org. transformation.carbonic anhydrases	TCA
IX	Bra039113	8	TCA / org. transformation.carbonic anhydrases	TCA
IX	Bra040520	8	TCA / org. transformation.carbonic anhydrases	TCA

IX	Bra007912	8	TCA / org. transformation.carbonic anhydrases	TCA
IX	Bra019192	19	tetrapyrrole synthesis.magnesium protoporphyrin IX methyltransferase	tetrapyrrole_synthesis
IX	Bra006878	19	tetrapyrrole synthesis.chlorophyll synthase	tetrapyrrole_synthesis
IX	Bra019190	19	tetrapyrrole synthesis.magnesium protoporphyrin IX methyltransferase	tetrapyrrole_synthesis
IX	Bra039813	34	transport.metal	transport
IX	Bra007035	34	transport.peptides and oligopeptides	transport
IX	Bra018096	34	transport.peptides and oligopeptides	transport
VIII	Bra006280	13	amino acid metabolism.synthesis.serine-glycine-cysteine group.cysteine	amino_acid_metabolism
VIII	Bra006280	13	amino acid metabolism.synthesis.serine-glycine-cysteine group.cysteine	amino_acid_metabolism
VIII	Bra025636	13	amino acid metabolism.synthesis.serine-glycine-cysteine group.cysteine	amino_acid_metabolism
VIII	Bra008720	13	amino acid metabolism.synthesis.serine-glycine-cysteine group.cysteine	amino_acid_metabolism
VIII	Bra001761	13	amino acid metabolism.synthesis.branched chain group.common.branched-chain amino acid aminotransferase	amino_acid_metabolism
VIII	Bra000914	13	amino acid metabolism.synthesis.aspartate family.methionine	amino_acid_metabolism
VIII	Bra017671	9	mitochondrial electron transport / ATP synthesis	ATP_synthesis
VIII	Bra011035	9	mitochondrial electron transport / ATP synthesis.NADH-DH.complex I	ATP_synthesis
VIII	Bra015815	31	cell.organisation	cell
VIII	Bra011099	31	cell.organisation	cell
VIII	Bra020277	31	cell.organisation	cell
VIII	Bra033737	31	cell.organisation	cell
VIII	Bra014865	31	cell.organisation	cell
VIII	Bra019493	31	cell.organisation	cell
VIII	Bra037560	31	cell.organisation	cell
VIII	Bra002568	31	cell.vesicle transport	cell
VIII	Bra021403	10	cell wall.pectin*esterases.PME	cell_wall
VIII	Bra038442	10	cell wall.modification	cell_wall
VIII	Bra038442	10	cell wall.modification	cell_wall
VIII	Bra013164	10	cell wall.modification	cell_wall
VIII	Bra035852	10	cell wall.degradation.pectate lyases and polygalacturonases	cell_wall
VIII	Bra023083	10	cell wall.modification	cell_wall
VIII	Bra001037	10	cell wall.cell wall proteins.RGP	cell_wall
VIII	Bra001037	10	cell wall.cell wall proteins.RGP	cell_wall
VIII	Bra008683	10	cell wall.cell wall proteins.RGP	cell_wall
VIII	Bra026264	10	cell wall.precursor synthesis.UGD	cell_wall
VIII	Bra028234	10	cell wall.precursor synthesis.UGD	cell_wall
VIII	Bra028234	10	cell wall.precursor synthesis.UGD	cell_wall
VIII	Bra028430	10	cell wall.precursor synthesis.UGD	cell_wall
VIII	Bra012613	10	cell wall.degradation.pectate lyases and polygalacturonases	cell_wall
VIII	Bra015635	33	development.unspecified	development
VIII	Bra025481	33	development.unspecified	development
VIII	Bra002403	33	development.unspecified	development
VIII	Bra002403	33	development.unspecified	development
VIII	Bra018454	33	development.unspecified	development
VIII	Bra014029	33	development.unspecified	development
VIII	Bra014029	33	development.unspecified	development
VIII	Bra026729	33	development.unspecified	development
VIII	Bra018532	33	development.unspecified	development
VIII	Bra026904	4	glycolysis.cytosolic branch.glyceraldehyde 3-phosphate dehydrogenase (GAP-DH)	glycolysis
VIII	Bra040213	4	glycolysis.cytosolic branch.glyceraldehyde 3-phosphate dehydrogenase (GAP-DH)	glycolysis
VIII	Bra016729	4	glycolysis.cytosolic branch.glyceraldehyde 3-phosphate dehydrogenase (GAP-DH)	glycolysis
VIII	Bra019722	4	glycolysis.cytosolic branch.glyceraldehyde 3-phosphate dehydrogenase (GAP-DH)	glycolysis
VIII	Bra016729	4	glycolysis.cytosolic branch.glyceraldehyde 3-phosphate dehydrogenase (GAP-DH)	glycolysis

VIII	Bra019722	4	glycolysis.cytosolic branch.glyceraldehyde 3-phosphate dehydrogenase (GAP-DH)	glycolysis
VIII	Bra040146	4	glycolysis.cytosolic branch.glyceraldehyde 3-phosphate dehydrogenase (GAP-DH)	glycolysis
VIII	Bra040213	4	glycolysis.cytosolic branch.glyceraldehyde 3-phosphate dehydrogenase (GAP-DH)	glycolysis
VIII	Bra004720	6	gluconeogenese/ glyoxylate cycle.citrate synthase	glyoxylate_cycle
VIII	Bra034655	17	hormone metabolism.auxin.induced-regulated-responsive-activated	hormone_metabolism
VIII	Bra011561	17	hormone metabolism.auxin.induced-regulated-responsive-activated	hormone_metabolism
VIII	Bra013115	17	hormone metabolism.gibberelin.induced-regulated-responsive-activated	hormone_metabolism
VIII	Bra002216	17	hormone metabolism.auxin.induced-regulated-responsive-activated	hormone_metabolism
VIII	Bra037506	17	hormone metabolism.jasmonate.induced-regulated-responsive-activated	hormone_metabolism
VIII	Bra004725	17	hormone metabolism.ethylene.induced-regulated-responsive-activated	hormone_metabolism
VIII	Bra017113	11	lipid metabolism.lipid transfer proteins etc	lipid_metabolism
VIII	Bra017113	11	lipid metabolism.lipid transfer proteins etc	lipid_metabolism
VIII	Bra005099	11	lipid metabolism.lipid transfer proteins etc	lipid_metabolism
VIII	Bra005099	11	lipid metabolism.lipid transfer proteins etc	lipid_metabolism
VIII	Bra005813	15	metal handling.binding, chelation and storage	metal_handling
VIII	Bra002217	15	metal handling	metal_handling
VIII	Bra012409	26	misc.glutathione S transferases	misc_otherPhosphate
VIII	Bra019747	26	misc.oxidases - copper, flavone etc.	misc_otherPhosphate
VIII	Bra027357	26	misc.GDSL-motif lipase	misc_otherPhosphate
VIII	Bra035235	26	misc.peroxidases	misc_otherPhosphate
VIII	Bra035235	26	misc.peroxidases	misc_otherPhosphate
VIII	Bra027040	26	misc.protease inhibitor/seed storage/lipid transfer protein (LTP) family protein	misc_otherPhosphate
VIII	Bra000775	26	misc.protease inhibitor/seed storage/lipid transfer protein (LTP) family protein	misc_otherPhosphate
VIII	Bra010602	26	misc.GCN5-related N-acetyltransferase	misc_otherPhosphate
VIII	Bra021101	26	misc.myrosinases-lectin-jacalin	misc_otherPhosphate
VIII	Bra035271	26	misc.UDP glucosyl and glucoronyl transferases	misc_otherPhosphate
VIII	Bra035271	26	misc.UDP glucosyl and glucoronyl transferases	misc_otherPhosphate
VIII	Bra004477	26	misc.short chain dehydrogenase/reductase (SDR)	misc_otherPhosphate
VIII	Bra027019	26	misc.invertase/pectin methylesterase inhibitor family protein	misc_otherPhosphate
VIII	Bra027717	26	misc.plastocyanin-like	misc_otherPhosphate
VIII	Bra027717	26	misc.plastocyanin-like	misc_otherPhosphate
VIII	Bra010598	26	misc.cytochrome P450	misc_otherPhosphate
VIII	Bra000737	26	misc.gluco-, galacto- and mannosidases	misc_otherPhosphate
VIII	Bra021292	26	misc.gluco-, galacto- and mannosidases	misc_otherPhosphate
VIII	Bra009313	26	misc.beta 1,3 glucan hydrolases	misc_otherPhosphate
VIII	Bra003273	26	misc.beta 1,3 glucan hydrolases.glucan endo-1,3-beta-glucosidase	misc_otherPhosphate
VIII	Bra003273	26	misc.beta 1,3 glucan hydrolases.glucan endo-1,3-beta-glucosidase	misc_otherPhosphate
VIII	Bra013634	26	misc.cytochrome P450	misc_otherPhosphate
VIII	Bra000544	35	not assigned.unknown	not_assigned
VIII	Bra026482	35	not assigned.unknown	not_assigned
VIII	Bra020252	35	not assigned.unknown	not_assigned
VIII	Bra031885	35	not assigned.unknown	not_assigned
VIII	Bra025747	35	not assigned.unknown	not_assigned
VIII	Bra025747	35	not assigned.unknown	not_assigned
VIII	Bra013763	35	not assigned.unknown	not_assigned
VIII	Bra037131	35	not assigned.unknown	not_assigned
VIII	Bra037808	35	not assigned.unknown	not_assigned
VIII	Bra004063	35	not assigned.unknown	not_assigned
VIII	Bra022699	35	not assigned.unknown	not_assigned
VIII	Bra004178	35	not assigned.unknown	not_assigned
VIII	Bra011349	35	not assigned.unknown	not_assigned
VIII	Bra001653	35	not assigned.unknown	not_assigned

VIII	Bra005668	35	not assigned.unknown	not_assigned
VIII	Bra014903	35	not assigned.unknown	not_assigned
VIII	Bra001685	35	not assigned.unknown	not_assigned
VIII	Bra003394	35	not assigned.unknown	not_assigned
VIII	Bra030769	35	not assigned.unknown	not_assigned
VIII	Bra014300	35	not assigned.unknown	not_assigned
VIII	Bra020090	35	not assigned.unknown	not_assigned
VIII	Bra013176	35	not assigned.unknown	not_assigned
VIII	Bra006014	1	PS.lightreaction.ATP synthase.beta subunit	Photosynthesis
VIII	Bra014499	29	protein.degradation.ubiquitin.E3.RING	protein
VIII	Bra015142	29	protein.degradation.ubiquitin.E3.RING	protein
VIII	Bra010263	29	protein.postranslational modification	protein
VIII	Bra000376	29	protein.degradation.autophagy	protein
VIII	Bra026582	29	protein.degradation.ubiquitin.E2	protein
VIII	Bra011680	29	protein.degradation.ubiquitin.E2	protein
VIII	Bra011680	29	protein.degradation.ubiquitin.E2	protein
VIII	Bra032062	29	protein.targeting.secretory pathway.unspecified	protein
VIII	Bra012376	29	protein.targeting.secretory pathway.unspecified	protein
VIII	Bra016344	29	protein.targeting.secretory pathway.unspecified	protein
VIII	Bra009542	29	protein.degradation.ubiquitin.ubiquitin	protein
VIII	Bra012710	29	protein.degradation.autophagy	protein
VIII	Bra012710	29	protein.degradation.autophagy	protein
VIII	Bra038481	29	protein.degradation.autophagy	protein
VIII	Bra027688	29	protein.degradation.ubiquitin.E2	protein
VIII	Bra022517	29	protein.targeting.secretory pathway.unspecified	protein
VIII	Bra006775	29	protein.degradation.ubiquitin.proteasom	protein
VIII	Bra026492	29	protein.degradation.ubiquitin.proteasom	protein
VIII	Bra026938	29	protein.degradation.ubiquitin.proteasom	protein
VIII	Bra029375	29	protein.degradation.ubiquitin.proteasom	protein
VIII	Bra009693	29	protein.degradation.ubiquitin.proteasom	protein
VIII	Bra020253	29	protein.synthesis.elongation	protein
VIII	Bra024986	29	protein.synthesis.ribosomal protein.eukaryotic.60S subunit.L3	protein
VIII	Bra018242	29	protein.synthesis.elongation	protein
VIII	Bra020251	29	protein.synthesis.elongation	protein
VIII	Bra020251	29	protein.synthesis.elongation	protein
VIII	Bra006661	29	protein.synthesis.elongation	protein
VIII	Bra018669	29	protein.synthesis.elongation	protein
VIII	Bra031601	29	protein.synthesis.elongation	protein
VIII	Bra002483	29	protein.synthesis.elongation	protein
VIII	Bra018669	29	protein.synthesis.elongation	protein
VIII	Bra031602	29	protein.synthesis.elongation	protein
VIII	Bra031605	29	protein.synthesis.elongation	protein
VIII	Bra001958	21	redox.heme	redox
VIII	Bra001958	21	redox.heme	redox
VIII	Bra017693	21	redox.dismutases and catalases	redox
VIII	Bra017693	21	redox.dismutases and catalases	redox
VIII	Bra025865	27	RNA.regulation of transcription.unclassified	RNA
VIII	Bra029155	27	RNA.regulation of transcription.MADS box transcription factor family	RNA
VIII	Bra031819	27	RNA.regulation of transcription.HB,Homeobox transcription factor family	RNA
VIII	Bra031819	27	RNA.regulation of transcription.HB,Homeobox transcription factor family	RNA
VIII	Bra000638	27	RNA.regulation of transcription.HB,Homeobox transcription factor family	RNA

VIII	Bra021401	27	RNA.regulation of transcription.AP2/EREBP, APETALA2/Ethylene-responsive element binding protein family	RNA
VIII	Bra025259	27	RNA.regulation of transcription.putative transcription regulator	RNA
VIII	Bra001640	27	RNA.regulation of transcription.AP2/EREBP, APETALA2/Ethylene-responsive element binding protein family	RNA
VIII	Bra035157	27	RNA.regulation of transcription.C2H2 zinc finger family	RNA
VIII	Bra009022	27	RNA.regulation of transcription.bHLH,Basic Helix-Loop-Helix family	RNA
VIII	Bra003356	27	RNA.regulation of transcription.MADS box transcription factor family	RNA
VIII	Bra020622	27	RNA.regulation of transcription.G2-like transcription factor family, GARP	RNA
VIII	Bra020622	27	RNA.regulation of transcription.G2-like transcription factor family, GARP	RNA
VIII	Bra001421	27	RNA.regulation of transcription.unclassified	RNA
VIII	Bra026635	27	RNA.regulation of transcription.ARR	RNA
VIII	Bra023242	27	RNA.regulation of transcription.unclassified	RNA
VIII	Bra019919	27	RNA.processing	RNA
VIII	Bra001900	27	RNA.regulation of transcription.Aux/IAA family	RNA
VIII	Bra033696	14	S-assimilation.ATPS	S-assimilation
VIII	Bra015853	16	secondary metabolism.flavonoids.isoflavones.isoflavone reductase	secondary_metabolism
VIII	Bra035066	30	signalling.light	signalling
VIII	Bra029500	30	signalling.receptor kinases.DUF 26	signalling
VIII	Bra009607	30	signalling.light	signalling
VIII	Bra020013	30	signalling.light	signalling
VIII	Bra012584	30	signalling.light	signalling
VIII	Bra012584	30	signalling.light	signalling
VIII	Bra038737	30	signalling.receptor kinases.leucine rich repeat XI	signalling
VIII	Bra038737	30	signalling.receptor kinases.leucine rich repeat XI	signalling
VIII	Bra000269	30	signalling.light	signalling
VIII	Bra040188	30	signalling.light	signalling
VIII	Bra040188	30	signalling.light	signalling
VIII	Bra000349	30	signalling.calcium	signalling
VIII	Bra028409	30	signalling.14-3-3 proteins	signalling
VIII	Bra035741	30	signalling.receptor kinases.leucine rich repeat XI	signalling
VIII	Bra012975	30	signalling.receptor kinases.leucine rich repeat XI	signalling
VIII	Bra028068	30	signalling.14-3-3 proteins	signalling
VIII	Bra040899	30	signalling.receptor kinases.leucine rich repeat XI	signalling
VIII	Bra000101	20	stress.biotic	stress
VIII	Bra010194	20	stress.abiotic.touch/wounding	stress
VIII	Bra010194	20	stress.abiotic.touch/wounding	stress
VIII	Bra018121	20	stress.abiotic.touch/wounding	stress
VIII	Bra014852	20	stress.abiotic.cold	stress
VIII	Bra014852	20	stress.abiotic.cold	stress
VIII	Bra002950	20	stress.abiotic.unspecified	stress
VIII	Bra016179	20	stress.abiotic.unspecified	stress
VIII	Bra021497	20	stress.biotic.PR-proteins	stress
VIII	Bra013717	20	stress.biotic	stress
VIII	Bra026653	20	stress.abiotic.unspecified	stress
VIII	Bra008025	20	stress.biotic.PR-proteins.proteinase inhibitors.trypsin inhibitor	stress
VIII	Bra008025	20	stress.biotic.PR-proteins.proteinase inhibitors.trypsin inhibitor	stress
VIII	Bra016510	20	stress.biotic	stress
VIII	Bra016511	20	stress.biotic	stress
VIII	Bra021941	34	transport.amino acids	transport
VIII	Bra036253	34	transport.p- and v-ATPases.H+-transporting two-sector ATPase	transport
VIII	Bra016752	34	transport.p- and v-ATPases	transport

VIII	Bra018952	34	transport.peptides and oligopeptides	transport
X	Bra030733	31	cell.vesicle transport	cell
X	Bra037782	10	cell wall.degradation.mannan-xylose-arabinose-fucose	cell_wall
X	Bra004892	10	cell wall.pectin*esterases.PME	cell_wall
X	Bra008663	33	development.unspecified	development
X	Bra035365	28	DNA.synthesis/chromatin structure	DNA
X	Bra035365	28	DNA.synthesis/chromatin structure	DNA
X	Bra003691	28	DNA.synthesis/chromatin structure	DNA
X	Bra031991	17	hormone metabolism.gibberelin.signal transduction	hormone_metabolism
X	Bra005246	26	misc.UDP glucosyl and glucoronyl transferases	misc_otherPhosphate
X	Bra005246	26	misc.UDP glucosyl and glucoronyl transferases	misc_otherPhosphate
X	Bra027806	26	misc.myrosinases-lectin-jacalin	misc_otherPhosphate
X	Bra025280	26	misc.cytochrome P450	misc_otherPhosphate
X	Bra015096	26	misc.gluco-, galacto- and mannosidases	misc_otherPhosphate
X	Bra001116	26	misc.oxidases - copper, flavone etc.	misc_otherPhosphate
X	Bra015656	12	N-metabolism.nitrate metabolism.NR	N-metabolism
X	Bra027300	35	not assigned.unknown	not_assigned
X	Bra021653	35	not assigned.unknown	not_assigned
X	Bra029054	35	not assigned.unknown	not_assigned
X	Bra038406	35	not assigned.unknown	not_assigned
X	Bra027077	35	not assigned.unknown	not_assigned
X	Bra008655	35	not assigned.unknown	not_assigned
X	Bra006682	35	not assigned.unknown	not_assigned
X	Bra037166	35	not assigned.unknown	not_assigned
X	Bra037166	35	not assigned.unknown	not_assigned
X	Bra041122	1	PS.lightreaction.photosystem I.PSI polypeptide subunits	Photosynthesis
X	Bra041122	1	PS.lightreaction.photosystem I.PSI polypeptide subunits	Photosynthesis
X	Bra021605	29	protein.degradation.ubiquitin.ubiquitin	protein
X	Bra015193	29	protein.degradation.ubiquitin.E3.SCF.FBOX	protein
X	Bra022635	29	protein.degradation.metalloprotease	protein
X	Bra023577	29	protein.targeting.chloroplast	protein
X	Bra007396	29	protein.synthesis.initiation	protein
X	Bra008608	29	protein.targeting.chloroplast	protein
X	Bra000501	29	protein.synthesis.ribosomal protein.eukaryotic.60S subunit.P2	protein
X	Bra032101	29	protein.synthesis.ribosomal protein.eukaryotic.40S subunit.SA	protein
X	Bra031540	21	redox.glutaredoxins	redox
X	Bra034639	27	RNA.regulation of transcription.bZIP transcription factor family	RNA
X	Bra030816	27	RNA.regulation of transcription.putative transcription regulator	RNA
X	Bra030816	27	RNA.regulation of transcription.putative transcription regulator	RNA
X	Bra040785	27	RNA.regulation of transcription.putative transcription regulator	RNA
X	Bra040785	27	RNA.regulation of transcription.putative transcription regulator	RNA
X	Bra013352	30	signalling.light	signalling
X	Bra028924	20	stress.abiotic.heat	stress
X	Bra028924	20	stress.abiotic.heat	stress
X	Bra030083	20	stress.abiotic.unspecified	stress
X	Bra026525	20	stress.abiotic.heat	stress
X	Bra015329	34	transport.misc	transport
X	Bra007603	34	transport.Major Intrinsic Proteins.PIP	transport
X	Bra026229	34	transport.misc	transport
X	Bra026229	34	transport.misc	transport
X	Bra010245	34	transport.unspecified cations	transport
