# Supplementary material for: Genome-wide transcriptome analysis reveals molecular pathways involved in leafy head formation of Chinese cabbage (Brassica rapa)
Source: Hortic Res. 2019 Dec 1;6:130. doi: 10.1038/s41438-019-0212-9 (PMC6885048; doi:10.1038/s41438-019-0212-9)
Supplement: Supplementary file 1 — Supplemental Figure S2 [file 41438_2019_212_MOESM1_ESM.docx]

# Supplementary Information

Figure S1. Cluster analysis by “ward” method for both biological repeats of the the three genotypes and four developmental stages.

Figure S2. The numbers of genes with differences in transcript abundance between different developmental stages/time points in CC-Z16 or PC-024 belonging to MapMan functional categories.

Figure S3. Barplots of the values of the 17 module eigengenes in CC-A03, CC-Z16 and PC-024.

Table S1. ANOVA test association for WGCNA gene clusters with genotype or phase or both genotype and phase effects.

Table S2. Gene lists for nine modules with genotype, phase or genotype by phase effect.

Table S3. Number of genes in Mapman categories of hierarchical clusters with genotype (V and VII) or genotype by phase (VIII, IX and X) effects.

Figure S4. Morphological characterization and leaf cellular organization of heading Chinese cabbage CC-022 with cylindrical head shape at seedling (S) and rosette (R) stage.

Table S4. The top 20 highest connected genes in cluster V & VII, VIII & X and IX based on Pearson correlation with coefficient of value 0.9.

Figure S5. Transcript abundance measured by qRT-PCR analysis of *BrTUB3-1*, *BrTUB3-2*, *BrTUB3-3*, *BrTUB1* and *BrTUB4* genes in heading chinese cabbage (CC-A03, CC-Z16 and Chiifu) and non-heading pak-choi (PC-024, PC-184 and PC-su) during plants development.

Table S5. Array design for the two-color Agilent customer *B. rapa* microarray (green/red) used in this study.

Table S6. Primers used for Quantitative Real-Time PC.

# Acknowledgments

Financial support for this research was provided by the Dutch Royal Academy of Sciences China Exchange Program (Grant No. 530-4CDP08), the China International Postdoctoral Exchange Fellowship Program and the International Cooperation Project in the Science and Technology Support Program of Hebei (Grant No. 17396315D and 2019YX023A). We thank staff from Unifarm, Wageningen University and Research and the Institute of Vegetables and Flowers Chinese Academy of Agricultural Sciences, for taking care of the plants.

# Conflict of interests

Xiaoxue Sun designed the experiment, performed the data analysis and contributed to manuscript writing. Ram Kumar Basnet performed the data analysis. Zhichun Yan did phenotyping and harvested plant materials. Johan Bucher did the microarray experiment. Chengcheng Cai helped in the data analysis. Jianjun Zhao was involved in discussions. Guusje Bonnema supervised the whole experiment and wrote the manuscript. All authors approved the final manuscript.

# Supplemental figures and tables

**Figure S1.** Cluster analysis by “ward” method for both biological repeats of the the three genotypes (two heading Chinese cabbage CC-A03, CC-ZA6 and one non-heading pak-choi PC-024) and four developmental stages (S-seedling, R-rosette, F-folding and H-heading). The two biological repeats were represented by “.1” and “.2”.

**Figure S2.** The numbers of genes with differences in transcript abundance between different developmental stages/time points in CC-Z16 or PC-024 belonging to MapMan functional categories. Genes with significant differences in transcript abundance were based on p-value ≤ 0.01 and an absolute value of log_2_Ratio ≥ 1.5. Week 2 corresponds to seedling stage of both CC-Z16 and PC-024; week 4 to rosette stage, week 6 to folding stage and week 7 to heading stage in CC-Z16, while weeks 4, 6 and 7 are all rosette stages for PC-024.

**Figure S3.** Barplots of the values of the 17 module eigengenes in CC-A03, CC-Z16 and PC-024. Two biological repeats for CC-A03, CC-Z16 and PC-024 were indicated by “.1” and “.2”. The most left 1th to 8th bar represent CC-A03 seedling (week 2), rosette (week 5), folding (week8) and heading (week 11) stages. 9^th^ to 16^th^ bars represent CC-Z16 seedling (week 2), rosette (week 4), folding (week 7) and heading (week 8) stages. 17^th^ to 24^th^ bar represent PC-024 seedling (week 2) and rosette (R1: week 4, R2: week 5 and R3: week 7) stages. 25^th^ to 36^th^ bar represent modules eigengenes for average of two biological repeats.

**Table S1.** ANOVA test association for WGCNA gene clusters with genotype or phase or both genotype and phase effects.

| Cluster | p-value_genotype | p-value_phase | Probe NO. |
| --- | --- | --- | --- |
| MEblack | 2.70E-07 | 0.652574619 | 1446 |
| MEdarkmagenta | 0.013269828 | 0.804125755 | 42 |
| MEdarkolivegreen | 0.002644206 | 0.743355948 | 48 |
| MEgrey | 0.022733117 | 0.195451571 | 119 |
| MElightyellow | 1.29E-05 | 0.682068436 | 104 |
| MEmidnightblue | 0.024825112 | 0.057093745 | 486 |
| MEsaddlebrown | 0.004320065 | 0.956819425 | 60 |
| MEsienna3 | 0.015020576 | 0.21755285 | 32 |
| MEdarkturquoise | 0.549452006 | 4.00E-10 | 85 |
| MEgreenyellow | 0.059924347 | 0 | 146 |
| MElightgreen | 0.16627194 | 1.43E-06 | 258 |
| MEsalmon | 0.796956832 | 1.90E-09 | 247 |
| MEdarkgrey | 0 | 0.003667838 | 82 |
| MEdarkred | 9.71E-05 | 0.003216904 | 159 |
| MElightcyan | 0.001162931 | 0.039830504 | 110 |
| MEsteelblue | 0.008535943 | 7.76E-07 | 522 |
| MEwhite | 0.011703878 | 0.01100325 | 426 |

*The association is significant at the 0.05 level in ANOVA analysis. The first part (light green) indicates genotype affected clusters; second part (green) indicates phase affected clusters; third part indicates genotype and phase affected clusters.*

**Table S3.** Number of genes in Mapman categories of hierarchical clusters with genotype (V and VII) or genotype by phase (VIII, IX and X) effects

| Categories | Cluster V & VII | Cluster VIII | Cluster IX | Cluster X |
| --- | --- | --- | --- | --- |
| protein | 98 | 33 | 17 | 8 |
| RNA | 81 | 18 | 16 | 5 |
| misc_otherPhosphate | 43 | 22 | 18 | 6 |
| signalling | 43 | 17 | 2 | 1 |
| stress | 34 | 15 | 16 | 4 |
| cell | 19 | 8 | 14 | 1 |
| cell_wall | 18 | 14 | 4 | 2 |
| transport | 15 | 4 | 3 | 5 |
| hormone_metabolism | 14 | 6 | 9 | 1 |
| aminoacid_metabolism | 13 | 6 | 6 | - |
| DNA | 13 |  | 3 | 3 |
| lipid_metabolism | 13 | 4 | 3 | - |
| metal_handling | 13 | 2 | 2 | - |
| secondary_metabolism | 8 | 1 | 7 | - |
| redox | 7 | 4 | 5 | 1 |
| development | 6 | 9 | 6 | 1 |
| Photosynthesis | 5 | 1 | 110 | 2 |
| ATP_synthesis | 4 | 2 | 2 | - |
| nucleotide_metabolism | 4 | - | 2 | - |
| N-metabolism | 3 | - | - | 1 |
| OPP | 3 | - | - | - |
| C1-metabolism | 2 | - | - | - |
| Cofactor_vitamine | 2 | - | 5 | - |
| minorCHOmetabolism | 2 | - | 1 | - |
| fermentation | 1 | - | - | - |
| TCA | 1 | - | 4 | - |
| tetrapyrrole_synthesis | 1 | - | 3 | - |
| glyoxylate_cycle | 1 | 1 | 1 | - |
| majorCHOmetabolism | 1 | - | 3 | - |
| Total number of genes | 468 | 197 | 262 | 41 |

**Figure S4.** Morphological characterization and leaf cellular organization of heading Chinese cabbage CC-022 with cylindrical head shape at seedling (S) and rosette (R) stage. The heading Chinese cabbage CC-022 was sown in 17cm pots in the greenhouse (Unifarm, Wageningen University & Research, 51^◦^59′11′′N latitude, 05^◦^39′52′′E longitude) in June 2016 for leaf observation.

**Table S4.** The top 20 highest connected genes in cluster V & VII, VIII & X and IX based on Pearson correlation with coefficient of value 0.9

|  | Ranking | All_Nodes | Annotation | Pathways | TAIR ID |
| --- | --- | --- | --- | --- | --- |
| Genotype affected cluster V and VII | 1 | Bra006775 | protein | protein.degradation.ubiquitin.proteasom | AT5G58290.1 |
|  | 2 | Bra038630 | not_assigned | not assigned.unknown | AT5G63440.2 |
|  | 3 | Bra003186 | nucleotide_metabolism | nucleotide metabolism.synthesis.pyrimidine.orotate phosphoribosyltransferase | AT3G54470.1 |
|  | 4 | Bra018184 | cell | cell.organisation | AT5G62700.1 |
|  | 5 | Bra007154 | lipid_metabolism | lipid metabolism.''exotics'' (steroids, squalene etc).trans-2-enoyl-CoA reductase (NADPH) | AT3G55360.1 |
|  | 6 | Bra020572 | cell | cell.organisation | AT4G14960.1 |
|  | 7 | Bra034554 | protein | protein.synthesis.initiation | AT4G33250.1 |
|  | 8 | Bra036611 | protein | protein.synthesis.ribosomal protein.eukaryotic.60S subunit.L18 | AT5G27850.1 |
|  | 9 | Bra006338 | protein | protein.folding | AT3G02530.1 |
|  | 10 | Bra014334 | protein | protein.synthesis.ribosomal protein.eukaryotic.60S subunit.L7 | AT2G01250.1 |
|  | 11 | Bra034717 | not_assigned | not assigned.unknown | AT5G03495.1 |
|  | 12 | Bra002054 | cell | cell.vesicle transport | AT2G17380.1 |
|  | 13 | Bra028864 | not_assigned | not assigned.unknown | AT5G02770.1 |
|  | 14 | Bra001788 | RNA | RNA.processing.ribonucleases | AT3G20390.1 |
|  | 15 | Bra024481 | protein | protein.synthesis.ribosomal protein.prokaryotic.chloroplast.50S subunit.L2 | AT4G36130.1 |
|  | 16 | Bra015007 | not_assigned | not assigned.unknown | AT3G24160.1 |
|  | 17 | Bra015153 | protein | protein.synthesis.initiation | AT2G04520.1 |
|  | 18 | Bra017461 | protein | protein.synthesis.ribosomal protein.eukaryotic.60S subunit.L7 | AT2G01250.1 |
|  | 19 | Bra001921 | amino_acid_metabolism | amino acid metabolism.degradation.aspartate family.methionine | AT3G23810.1 |
|  | 20 | Bra013406 | RNA | RNA.regulation of transcription.HSF,Heat-shock transcription factor family | AT4G19630.1 |

|  | Ranking | All_Nodes | Annotation | Pathways | TAIR ID |
| --- | --- | --- | --- | --- | --- |
| Genotype by phase affected cluster VIII | 1 | Bra000349 | signalling | signalling.calcium | AT2G44310.1 |
|  | 2 | Bra028430 | cell_wall | cell wall.precursor synthesis.UGD | AT5G39320.1 |
|  | 3 | Bra014865 | cell | cell.organisation | AT3G53750.1 |
|  | 4 | Bra001037 | cell_wall | cell wall.cell wall proteins.RGP | AT3G02230.1 |
|  | 5 | Bra028068 | signalling | signalling.14-3-3 proteins | AT1G35160.1 |
|  | 6 | Bra008683 | cell_wall | cell wall.cell wall proteins.RGP | AT5G15650.1 |
|  | 7 | Bra012376 | protein | protein.targeting.secretory pathway.unspecified | AT1G23490.1 |
|  | 8 | Bra016344 | protein | protein.targeting.secretory pathway.unspecified | AT2G47170.1 |
|  | 9 | Bra016752 | transport | transport.p- and v-ATPases | AT1G12840.1 |
|  | 10 | Bra026938 | protein | protein.degradation.ubiquitin.proteasom | AT3G26340.1 |
|  | 11 | Bra040146 | glycolysis | glycolysis.cytosolic branch.glyceraldehyde 3-phosphate dehydrogenase (GAP-DH) | AT1G13440.1 |
|  | 12 | Bra040213 | glycolysis | glycolysis.cytosolic branch.glyceraldehyde 3-phosphate dehydrogenase (GAP-DH) | AT1G13440.1 |
|  | 13 | Bra009693 | protein | protein.degradation.ubiquitin.proteasom | AT5G23540.1 |
|  | 14 | Bra026904 | glycolysis | glycolysis.cytosolic branch.glyceraldehyde 3-phosphate dehydrogenase (GAP-DH) | AT3G04120.1 |
|  | 15 | Bra028234 | cell_wall | cell wall.precursor synthesis.UGD | AT5G39320.1 |
|  | 16 | Bra033737 | cell | cell.organisation | AT5G44340.1 |
|  | 17 | Bra006014 | Photosynthesis | PS.lightreaction.ATP synthase.beta subunit | AT5G08680.1 |
|  | 18 | Bra029375 | protein | protein.degradation.ubiquitin.proteasom | AT5G23540.1 |
|  | 19 | Bra016729 | glycolysis | glycolysis.cytosolic branch.glyceraldehyde 3-phosphate dehydrogenase (GAP-DH) | AT3G04120.1 |
|  | 20 | Bra021292 | misc_otherPhosphate | misc.gluco-, galacto- and mannosidases | AT3G18080.1 |

|  | Ranking | All_Nodes | Annotation | Pathways | TAIR ID |
| --- | --- | --- | --- | --- | --- |
| Genotype by phase affected cluster IX and X | 1 | Bra009244 | Photosynthesis | PS.calvin cycle.RPE | AT5G61410.1 |
|  | 2 | Bra035349 | Photosynthesis | PS.photorespiration.phosphoglycolate phosphatase | AT5G36700.1 |
|  | 3 | Bra020809 | not_assigned | not assigned.unknown | AT4G35250.1 |
|  | 4 | Bra008166 | not_assigned | not assigned.unknown | AT1G74730.1 |
|  | 5 | Bra021555 | Photosynthesis | PS.photorespiration.glycolate oxydase | AT3G14415.1 |
|  | 6 | Bra031197 | not_assigned | not assigned.unknown | AT2G21530.1 |
|  | 7 | Bra015897 | not_assigned | not assigned.unknown | AT1G74730.1 |
|  | 8 | Bra011918 | not_assigned | not assigned.unknown | AT1G55480.1 |
|  | 9 | Bra013517 | Photosynthesis | PS.lightreaction.photosystem II.PSII polypeptide subunits | AT4G21280.1 |
|  | 10 | Bra018955 | Photosynthesis | PS.lightreaction.photosystem I.PSI polypeptide subunits | AT1G52230.1 |
|  | 11 | Bra031624 | Photosynthesis | PS.lightreaction.photosystem I.PSI polypeptide subunits | AT1G08380.1 |
|  | 12 | Bra037761 | Photosynthesis | PS.lightreaction.photosystem I.PSI polypeptide subunits | AT5G64040.1 |
|  | 13 | Bra021556 | Photosynthesis | PS.photorespiration.glycolate oxydase | AT3G14420.1 |
|  | 14 | Bra000663 | redox | redox.ascorbate and glutathione.ascorbate | AT4G09010.1 |
|  | 15 | Bra000687 | Photosynthesis | PS.lightreaction.ATP synthase.delta chain | AT4G09650.1 |
|  | 16 | Bra005211 | not_assigned | not assigned.unknown | AT2G37240.1 |
|  | 17 | Bra016522 | Photosynthesis | PS.lightreaction.photosystem II.LHC-II | AT1G19150.1 |
|  | 18 | Bra018415 | Photosynthesis | PS.lightreaction.other electron carrier (ox/red).ferredoxin | AT1G10960.1 |
|  | 19 | Bra013013 | misc_otherPhosphate | misc.rhodanese | AT5G23060.1 |
|  | 20 | Bra016353 | amino_acid_metabolism | amino acid metabolism.synthesis.central amino acid metabolism.alanine.alanine aminotransferase | AT1G23310.1 |

**Figure S5.** Transcript abundance measured by qRT-PCR analysis of *BrTUB3-1*, *BrTUB3-2*, *BrTUB3-3*, *BrTUB1* and *BrTUB4* genes in heading chinese cabbage (CC-A03, CC-Z16 and Chiifu) and non-heading pak-choi (PC-024, PC-184 and PC-su) during plants development. CC-A03, CC-Z16, PC-024 and PC-184 were grown in 2014 in a greenhouse. CC-Chiifu and PC-su were grown in 2015 in a field in Beijing, China. Green histogram represents CC-A03; Blue histogram represents CC-Z16; Pink histogram represents PC-024; Yellow histogram represents PC-184; Grey histogram represents CC-Chiifu and purple histogram represents PC-su.

**Table S5.** Array design for the two-color Agilent customer *B. rapa* microarray (green/red) used in this study. (A) Sample information for array materials: tissue used was harvested from central young emerging leaves. (B) Schematic diagram for the two-color array design loop. Cy3 dye is green, Cy5 dye is red.

| A. | T1 | | T2 | T3 | T4 |
| --- | --- | --- | --- | --- | --- |
| Phase | Seedling | | Rosette | Folding | Heading |
| CC-A03 | Week 2 | | Week 5 | Week 8 | Week 11 |
| CC-Z16 | Week 2 | | Week 4 | Week 6 | Week 7 |
|  | Seedling | Rosette-1 | | Rosette-2 | Rosette-3 |
| PC-024 | Week 2 | | Week 4 | Week 6 | Week 7 |

**Table S6.** Primers used for Quantitative Real-Time PC

| *B. rapa* gene | | BRAD ID | | Forward 5’—3’ | | Reverse 5’—3’ | |
| --- | --- | --- | --- | --- | --- | --- | --- |
| *BrTUB3-1* | *Bra018184* | | TTTTGCTCCGCTCACCTCAA | | TTGTGCTCATTTTGCCACGG | |  |
| *BrTUB3-2* | *Bra010114* | | AACGCTACGCTGTCTGTTCA | | AGAGTTGAGCTGACCAGGGA | |  |
| *BrTUB3-3* | *Bra019493* | | TCAAGGGTTTCAGGTGTGCC | | TGAACAGACAAGGTGGCGTT | |  |
| *BrTUB1* | *Bra015815* | | TCCACCAGCTCGTTGAGAAC | | TAGAGAGCAAGTCACGCCAC | |  |
| *BrTUB4* | *Bra033737* | | ACGACATCTGCTTCCGTACC | | GAAGTGGAGCCTGGGGAAAG | |  |
